# Molecule characterization of chemosensory and metabolism-related genes in the proboscis of *Athetis lepigone*

**DOI:** 10.3389/fphys.2023.1287353

**Published:** 2023-12-22

**Authors:** Cai-Hong Tian, Xiao-Guang Liu, Cun-Yi Xu, Jian-Rong Huang, Jun-Feng Fu, Gen-Song Wang, Jun-Yi Zhang, Guo-Ping Li, Xin-Ming Yin, Hong-Qiang Feng

**Affiliations:** ^1^ Henan Key Laboratory of Crop Pest Control, MOA Key Regional Crop Integrated Pest Management (IPM) Laboratory in Southern Part of Northern China, International Joint Research Laboratory for Crop Protection of Henan, Entomological Radar Station Zero of Henan Province for Field Scientific Observation and Research, Institute of Plant Protection, Henan Academy of Agricultural Sciences, Zhengzhou, China; ^2^ Henan International Laboratory for Green Pest Control, Henan Engineering Laboratory of Pest Biological Control, College of Plant Protection, Henan Agricultural University, Zhengzhou, China; ^3^ Yuzhou Plant Protection and Quarantine Station, Yuzhou, China

**Keywords:** *Athetis lepigone*, scanning electron microscopy, proboscis transcriptome, chemosensory genes, metabolism-related genes, quantitative real-time PCR

## Abstract

**Introduction:** The moth species *Athetis lepigone* (Möschler) (Lepidoptera: Noctuidae), which has recently been identified as a pest of summer maize (*Zea mays* L.) in China, has demonstrated a rapid proliferation with in the Huang-Huai-Hai Plain region since its initial discovery in Hebei Province in 2005. It has become a prevalent pest of corn crops, and its ability to adapt quickly to its surroundings is currently being investigated. One of the key characteristics of its siphoning mouthparts is not only the feeding apparatus itself but also the chemosensory organs that enable the detection of chemical signals from the surrounding environment. However, there is a lack of comprehensive research on the genes responsible for chemosensory and metabolic mechanisms in the proboscises of male and female *A. lepigone* adults.

**Methods:** In this study, we utilized transcriptome analysis to identify a total of fifty chemosensory genes from six distinct families, including 19 odorant-binding proteins (OBPs), 22 chemosensory proteins (CSPs), one co-receptor (Orco), six odorant receptors (ORs), four ionotropic receptors (IRs), and two sensory neuron membrane proteins (SNMPs) in the proboscis. Notably, seven OBPs, two CSPs, and one OR were discovered for the first time. Additionally, fourteen genes related to metabolism, including cytochrome P450 (CYPs) and carboxylesterases (CXEs), were also identified. Furthermore, a qualitative analysis was conducted on the relative transcript levels of eight related genes. The expression of 21 annotated chemosensory and metabolic genes was compared between *A. lepigone* adults and larvae using qRT-PCR, revealing tissue specificity. The majority of genes exhibited predominant expression in the antennae and proboscis during the adult stage, while showing slight expression in the combination of sixth-instar larval head oral appendages (maxilla, labium, and antenna) and pheromone gland-ovipositors of female adults.

**Results/discussion:** Our study points to a new pest control strategies that these newly discovered genes have the potential to serve as targets for enhancing future pest control, including mating disruption and the use of food attractants. And it would be advantageous to ascertain the distribution of chemosensory gene expression and gain insights into the functionalities of these genes, thereby establishing a novel theoretical framework for the advancement of eco-friendly pesticides and efficient pest management strategies in the future.

## 1 Introduction

*Athetis lepigone* (Möschler) (Lepidoptera: Noctuidae), a newly discovered summer maize (*Zea mays* L.) pest in China, has been widespread in Eurasia and other Asian countries since it was first reported in Liaoning Province (Chen, 1999). Subsequently, the larvae of this notorious species spread quickly in the Huang-Huai-Hai River Plain region because of their polyphagous and root-damaging nature (Jiang et al., 2008; Wang et al., 2011; Zhu et al., 2012). In the summer maize fields of Hebei, one of the most seriously affected provinces, the *A. lepigone* larvae often chisel in and feed on young prop roots and stems, resulting in lodging, wilting, and eventually death of the maize seedlings. Seed-dressing with systemic insecticides, such as combination of 20% cyantraniliprole and 20% thiamethoxam, is commonly employed on an annual basis during the sowing period to mitigate the presence of root-eating pests. Although there has been a notable decrease in corn damage caused by *A. lepigone* larvae in recent years, the population of adult pests remains high. The average annual count of trapped moths from 2019 to 2020 has consistently hovered around 14,966.5, surpassing the numbers observed for *Helicoverpa armigera* and *Ostrinia furnacalis* in the central and southern regions of Hebei Province by factors of 5.75 and 7.05, respectively (Dong et al., 2021). The cause for the abundance of moths despite a limited number of larval populations is still not understood. In addition, the migratory nature of this species and its seasonal migration patterns in northern China were previously unknown (Fu et al., 2014). However, In July 2016, our laboratory utilized a vertical-looking radar (VLR) and a vertical-pointing searchlight trap to observe the migratory behavior of second-generation moths at Yuanyang, Xinxiang City, located in the southern region of Northern China (Huang et al., 2018). It was discovered that these moths reached high altitudes during their migration. Consequently, there is a significant risk of *A. lepigone* outbreak on summer maize fields when suitable environmental conditions are present (Liang et al., 2021). Therefore, it is necessary to continue to popularize and apply ecological regulation technologies against *A. lepigone* to control its damage. Moreover, alternative strategies for controlling *A. lepigone* infestations are urgently required.

In *A. lepigone* adult individuals, siphoning mouthparts serve as distinctive feeding organs, characterized by extended proboscis tubes that are interconnected by the outer jaw leaves. The distal end of the proboscis tube features a fissure that facilitates the inhalation of liquid, constituting approximately 5%–20% of the length of the total proboscis tube. The detection of chemical cues in the surrounding environment is accomplished through the olfactory system which relies on specialized antennal sensilla structures and minute components located on the maxillary palps (Tian et al., 2015; Li et al., 2022). Scanning electron microscopy (SEM) analysis has revealed that the olfactory chemosensilla are priedominantly localized on the maxillary palps and proboscis, while the gustatory chemosensilla are primarily found on the palps, maxillary epipharynx, and galea structures (Albert, 1980; Kent and Hildebrand, 1987; Asaoka and Shibuya, 1995). Typically, most adult lepidopterans possess elongated proboscis and primarily feed on nectar derived from host plants. Sensory sensilla neurons located on the proboscis and maxillary palps of both larvae and adults play a crucial role in detecting volatile and nonvolatile sensory cues. These neurons directly interact with odors or saccharides, facilitating activities such as locating host plants, feeding, egg-laying and mating, or identifying suitable oviposition sites (Shankar et al., 2016; Guo P. et al., 2018; Sun et al., 2022; Guo et al., 2023). This sensory characteristic frequently employed in the surveillance and management of varies of crop pests. It is evident that certain volatiles emitted by host foods play a pivotal role in the selection of host plants by herbivorous pests. Furthermore, botanical attractants, which are volatile food-based substances derived from preferred plants, can serve as significant elements in green pest control (Cai et al., 2018). So, exploiting the olfactory system to manage populations through the entrapment of *A. lepigone* adult insects might represent a highly effective alternative strategy for environmentally friendly control (Venthur and Zhou, 2018).

Numerous insect species possess sensory systems that are highly specialized in detecting odor information and typically use plant-produced chemicals as precursors for their own chemical signals, a phenomenon that has evolved over an extended period of time and also influences various crucial behavioral activities. Previous research has demonstrated that insects possess the ability to effectively convert diverse chemical signals through the utilization of various functional proteins expressed in senseory organs, including the antennae, proboscis, and maxillae (Chen et al., 2022). Furthermore, there have been preliminary investigations into the sensory structures in the proboscis of *A. lepigone* have also been preliminarily studied (Hu et al., 2021). However, studies on the chemosensory systems of this species have predominantly focused on the adult antennae, resulting in a limited understanding of the chemosensory receptors present in the proboscis. This lack of knowledge impedes efforts to control the population of *A. lepigone*. In the proboscis and labial palps of adult *H. armigera*, a total of eighty-four chemosensory genes, including fourteen novel genes were identified (Guo et al., 2018b). Zhang et al. (2016), Zhang et al. (2017a) conducted a study using *A. lepigone* antennal transcriptomic data and identified eighty candidate chemosensory receptors, consisting of sixty-one transcripts encoding odorant receptors (ORs) and nineteen for ionotropic receptors (IRs), as well as twenty-eight odorant-binding proteins (OBPs), twenty chemosensory protein genes (CSPs), and twenty carboxylesterase genes (CXEs) (Zhang et al., 2016; Zhang et al., 2017a). However, the specific types of sensilla present in the proboscis and the distribution of chemosensory and metabolism-related genes in the proboscis of *A. lepigone* adults remain unknown. Additionally, the investigation of the chemosensory and metabolism-related genes expressed in the proboscis and pheromone gland-ovipositor, similar to the antennae, was of particular significance.

As a newly discovered pest, although more and more few genetic resources are available for *A. lepigone*. There is a lack of comprehensive research on the genes responsible for chemosensory and metabolic mechanisms in the proboscises of male and female *A. lepigone* adults. In order to validate the aforementioned findings, we initially conducted an observation and analysis of the ultrastructure of the proboscis of *A. lepigone* adults through electron microscope scanning. Additionally, we employed the illumine sequencing platform to sequence the transcripts, subsequently investigating and thoroughly characterizing the genes associated with chemosensation and metabolism. The differential expression of potential genes was verified using quantitative real-time PCR (qRT-PCR). Furthermore, we utilized qRT-PCR to further analyze the expression levels of annotated genes in various tissues, such as the sixth-instar larval head oral appendages (LA; maxilla, labium, and antenna), adult male proboscis (MP), adult female proboscis (FP), adult male antennae (MA), adult female antennae (FA), and pheromone gland-ovipositor in female adults (OV), in order to elucidate tissue specificity.

## 2 Materials and methods

### 2.1 Insect-rearing, RNA extraction, and scanning electron microscope observation

Wild-type *A*. *lepigone* larvae were collected from summer corn fields in Xun County (35°40′N, 114°21′E) of northern Henan Province, China. No endangered or protected species were included in the field collection. The larvae were reared on an artificial diet, and the adults were placed in an etamine-covered plastic cup and fed with a 5% honey solution. The hatched eggs were transferred to a box and fed with the same artificial diet. All insect cultures were kept at 26°C ± 0.5°C and 70%–80% relative humidity, with a photoperiod of 14/10 h light/dark (Tian et al., 2012; Tian et al., 2020).

Newly emerged adults were collected (11th generations), and the proboscis from a total of 20 male and female adults were scanned using a Scanning electron microscopy (SEM). The methodology employed in this study followed the procedures outlined by Tian et al. (2021). Briefly, the proboscis was cut from the heads of both male and female adults, subsequently subjected to ultrasonic washing with isotonic saline, and finally fixed in a 2.5% glutaraldehyde solution overnight. The samples were then dehydrated using a series of graded ethanol concentrations (70%, 80%, 85%, 90%, and 95% for 15 min each, followed by two rounds of 100% ethanol for 20 min each). Subsequently, the samples were placed on filter paper and allowed to naturally air dry overnight on an Ultra Clean Ben (HS-840; AIRTECH) surface. The dried samples were exposed at multiple angles and coated using a sputter coater. The treated samples were observed and imaged using a HITACHI SU3500 SEM (Hitachi, Japan) at 15.00 kV under full vacuum. The proboscises were highly sclerotized, and the structures were not significantly affected.

Proboscis samples from 50 male and 50 female adults from the same 11th generations were collected separately and immediately stored in liquid nitrogen until RNA extraction. RNA was isolated from the proboscis following the procedure described by Tian et al. (2020), and RNA quality was verified using a 2100 Bioanalyzer (Agilent Technologies, CA, USA) with a minimum RNA integration value of 6. Total RNA was isolated from the proboscis of male (OD260/280 = 1.9) and female adults (OD260/280 = 2.0) using an SV Total RNA Isolation System (Promega, USA). For different samples, three biological replicates were conducted, with three technical replicates/biological replicates. The purified total RNA was used for transcriptome sequencing and qRT- PCR.

### 2.2 cDNA library synthesis and illumina sequencing

cDNA was synthesized according to the manufacturer’s protocol provided by Illumina (Illumina, San Diego, CA, USA). Briefly, 20 µg of total RNA from the proboscis of male and female *A*. *lepigone* was digested using DNase I (Sigma, USA), and then the mRNA was purified using oligo (dT) magnetic beads and broken into small fragments (100–400 bp). The mRNA fragments were used as templates and cDNA was generated using reverse transcription (Invitrogen, Carlsbad, CA, USA) with random hexamer primers. The quantity and quality of both male and female proboscis libraries were analyzed using an Agilent 2100 Bioanalyzer and an ABI Step OnePlus Real-Time PCR System. Both male and female proboscis cDNA libraries with 200-bp insert sizes were sequenced using an Illumina HiSeq2000™ (Illumina) at the Beijing Genome Institute (Shenzhen, China).

### 2.3 Sequence assembly and annotation

Firstly, the reads underwent a cleaning process involving the removal of adaptors, followed by the elimination of low-quality and disorganized read ends. In order to facilitate assembly, the containing ambiguous bases and base calls were trimmed, and any latent primer or adapter sequences specific to male and female *A*. *lepigone* were removed using Trinity software. The output of Trinity software was then clustered using default parameters from the TGICL (Grabherr et al., 2011). The resulting transcripts were assembled and clustered using Chrysalis clusters software, and the longest sequence within each cluster was retained and designated as a unigene. The unigene database had combined cluster sequences and singletons. Unigenes were aligned against the non-redundant (nr) and Swiss-Prot protein (Swiss-Prot) databases (Wilkins et al., 1999). Blast2GO software was used to annotate the genes with a cut-off e-value of 10^–5^ (BLASTx tool) (Conesa et al., 2005). Protein-coding region analysis was conducted using the ORF finder online server (http://www.ncbi.nlm.nih.gov/gorf/gorf.html) based on BLAST results. The protein sequences were uploaded to the SignaIP 4.1 server to predict signal peptides (Petersen et al., 2011). TMHMM Server (version 2.0) was used to predict the domains of transmembrane helices in proteins (Krogh et al., 2001). The amino acid (aa) sequences of the ORFs were used to compute pI/Mw (Bjellqvist et al., 1994).

### 2.4 Phylogenetic analysis

The amino acid sequences of ORs, IRs, OBPs, CSPs, CXEs, and CYPs were separately input into Clustal Omega (Sievers et al., 2011) for alignment and edited using GeneDoc software (http://nrbsc.org/gfx/genedoc/index.html).

The orthologous genes were identified using the best reciprocal BLAST hits. Amino acid sequences of orthologous genes were obtained by searching the website (http://www.ncbi.nlm.nih.gov/protein/) using the subject ids of the unigene annotations. Phylogenetic trees were constructed based on the lepidopteran insect dataset using Clustal Omega (https://www.ebi.ac.uk/Tools/msa/clustalo/) and edited using online software (https://itol.embl.de/tree/223881124421491674564422). One thousand bootstrap replicates were run to assess node support.

### 2.5 Gene nomenclature

Based on the unified nomenclature system and traditional conventions, the candidate unigenes were named with four-letter species abbreviations, including an initial uppercase letter of the genus and three lower-case letters of the species (e.g., *H. armigera* = Harm and *Spodoptera exigua* = Sexi) (Tian et al., 2015). Candidate genes were numbered from one onward (e.g., Alep PBP genes, ORF length (aa) = 167, named AlepPBP1).

### 2.6 Different gene expression

The relative gene expression of female and male adult proboscises was obtained using the Fragments Per Kilobase of transcripts per million mapped reads (FPKM) method (Mortazavi et al., 2008). Statistical comparisons between different samples were performed using IDEG6 software (Romualdi et al., 2003). The variations in different gene expression were calculated for the sex differences (i.e., female adults and male adults [ALMA/ALFA]).

### 2.7 Gene expression analysis via qRT-PCR

The present study employed to conduct expression profiling of candidate chemosensory and metabolism-related genes. Tissues, containing oral appendages (maxilla, labium, and antenna) of the sixth-instar larvae (LA), male adult proboscis (MP), female adult proboscis (FP), male adult antennae (MA), female adult antennae (FA), and pheromone gland-ovipositor of female adults (OV) from pupae aged one to 3 days, were collected separately. Three biological replicates were obtained for each sample, and total RNA extraction was performed following previously established protocols. The first-strand cDNA was synthesized from 2 µg total RNA using a PrimeScript RT reagent Kit with gDNA Eraser (TaKaRa, Tokyo, Japan), and SYBR Premix Ex Taq™ (Tli RNase H Plus; TaKaRa) was used to perform quantitative real-time PCR according to the manufacturer’s protocols. Primers were designed using Primer3 (http://simgene.com/Primer3). All qRT-PCR primer pairs are listed in Supplementary Table S1. All the primer pairs were used to perform gradient PCR and optimized for an annealing temperature of 60°C. All the PCR products were separated using 1.5% agarose gel electrophoresis and confirmed by sequencing (Biological Engineering [Shanghai] Co., Ltd.). The stability and consistency of amplification curves (S-shaped) and CT values for the *β*-actin gene across different tissues were carefully assessed, as per the methodology described by Zhang et al. (2019).

Data is represented as mean ± SEM. All data were analyzed using one-way analysis of variance (ANOVA) and compared using Duncan’s New Multiple Range Method. All statistical analyses were performed using the IBM SPSS Statistics software (version 22.0; SPSS Inc., Chicago, IL, USA). Transcript levels were analyzed using the 2^−ΔΔCt^ method (Livak and Schmittgen, 2001).

## 3 Results

### 3.1 Morphology and sensilla types on *A. lepigone* proboscis

**FIGURE 1:**
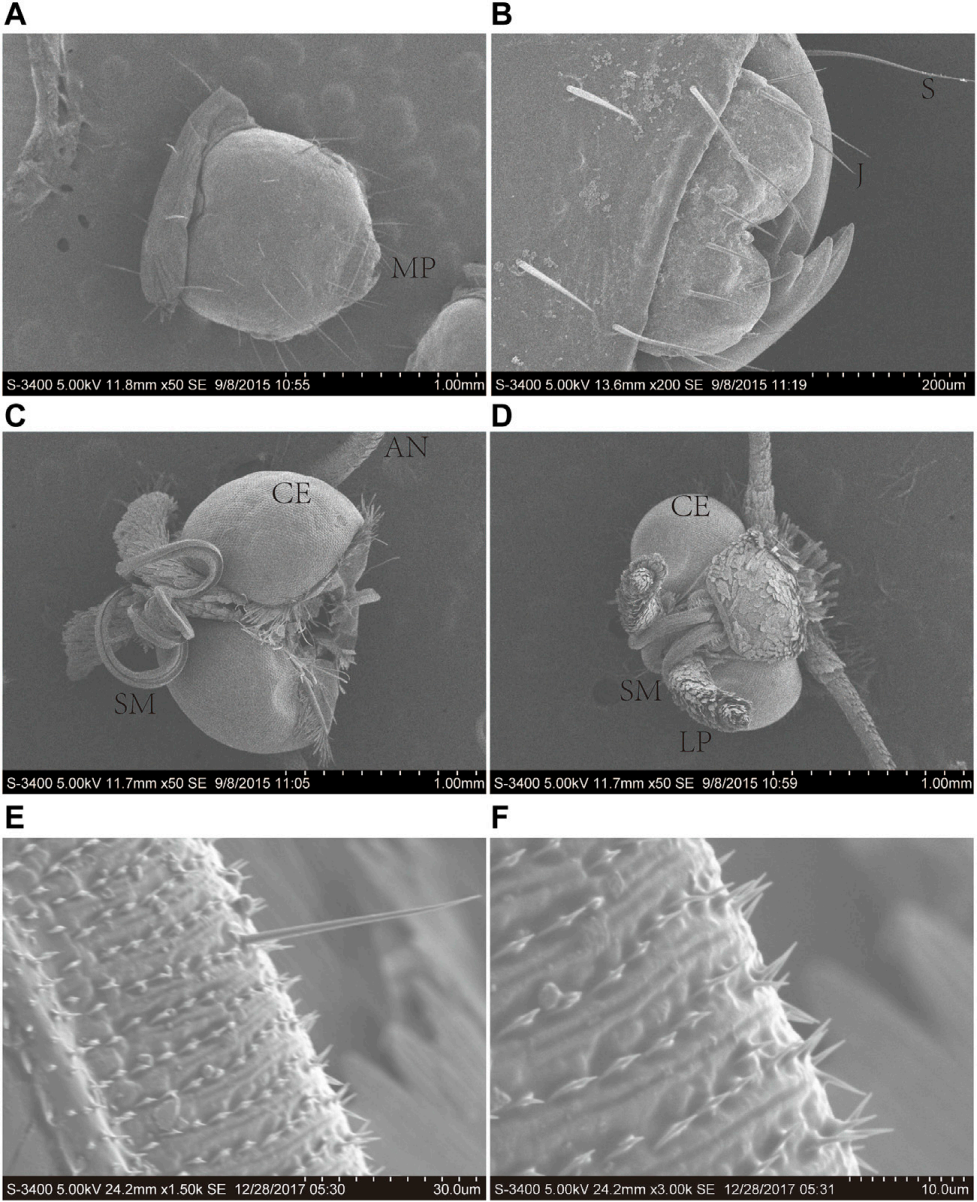
Scanning electron micrographs of the head of the sixth-instar larva, male, and female head of *A. lepigone.* **(A)** The sixth instar larval head and oral appendages. **(B)** The sixth instar larval head oral appendages. **(C)** The head appendages of male *A. lepigone*. **(D)** The head appendages of female *A. lepigone.* **(E)** The proboscis of male *A. lepigone*. **(F)** The proboscis of female *A. lepigone*. MP: moth part; S: sensilla trichodea; J: jaw; CE: compound eyes; AN: antenna; SM: siphoning mouthparts; LP: labial palpi.

The proboscis of *A. lepigone* adults consisted of two elongated maxillary galeae (Figures 1A, B), and both males and females exhibited similar morphological characteristics. The average lengths of the proboscises in males and females were 3.31 ± 2.25 and 3.27 ± 2.32 mm, respectively (Figures 1C, D). The proboscis exhibite “spines” and numerous sensilla in the tip region (Figures 1E, F). In the proboscis and mouthparts of *A. lepigone* larvae, the majority of sensilla were identified as trichoid (Ts), styloconica (STs), and chaeticum (Cs) based on their shape (Figures 1A, B). The sensillum types present in the proboscises of male adults *A. lepigone* were identified as trichoid and chaeticum, while in female *A. lepigone* adults, the sensillum types were basiconica and styloconicum. Additionally, the majority of sensilla were found to be distributed anteriorly along approximately one-third of the proboscis tubes. The number of sensilla gradually decreased from the base to distal end of the distribution (Figures 1E, F).

### 3.2 In silico summary of the transcriptome

RNA samples extracted from the male and female proboscis of *A. lepigone* were subjected to sequencing using the Illumina HiSeq™ 2000 platform. Following the removal of adaptor sequences, contaminating reads, and trimming, a total of 52,438,848 and 52,139,649 clean reads were obtained from the male and female proboscis samples, respectively, resulting in the generation on fo high-quality contigs; All the raw reads of male and female proboscis data were collected and stored in the Genome Sequence Archive (Chen et al., 2021) at the National Genomics Data Center (GSA: CRA012317) (CNCB-NGDC Members and Partners, 2022). These contigs underwent further assembly through paired-end joining and gap-filling, resulting in the formation of 122, 277 unigenes. In the male samples, 61,156 unigenes were generated from each assembly read, while in the female samples, 61,121 unigenes produced frome the assembly read. Following clustering, a total of 13,180 distinct clusters with 47,941 singletons were obtained for males, and 13,327 distinct clusters with 47,829 singletons were obtained for females. The assembly process yielded a dataset of 38,235,440-nt with a mean length of 723 nt (N_50_ = 1083 nt). The length distributions revealed that 12,137 unigenes (22.95% of all unigenes) were longer than 1,000 nt (Table 1).

**TABLE 1:** Overview of *A*. *lepigone* proboscis transcriptome assembly.

	Sample	Total number	Total length (nt)	Mean length (nt)	N50	Total consensus sequences	Distinct clusters	Distinct singletons
Contig	Male	112,579	34,159,125	303	464	-	-	-
	Female	118,889	34,711,899	292	439	-	-	-
Unigene	Male	61,121	34,845,610	570	891	61,121	13,180	47,941
	Female	61,156	33,479,526	547	847	61,156	13,327	47,829
	All	52,879	38,235,440	723	1083	52,879	15,641	37,238

**FIGURE 2:**
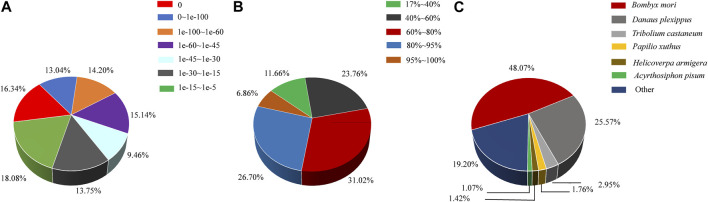
Characteristics of homology search of assembled unigenes against the nr database. **(A)** E-value distribution of *A. lepigone* proboscis unigenes. **(B)** Similarity distribution annotation of unigenes in the NR database. **(C)** Species distribution; the first hits of each unigene were used for analysis.

All transcripts were annotated using the NCBI BLASTX software through a homology-based search against the GenBank nr and trEMBL databases. It was observed that 41.29% of the mapped unigenes exhibited strong homology (top hits:e-value <1.0e-45), while the remaining 58.71% of the unigenes fell within the range of 1.0e-5 to 1.0e-45 (Figure 2A). In terms of similarity distribution, 33.56% of the unigenes displayed a similarity greater than 80%, while 31.02% fell within the range of 60%–80%, and 35.42% fell within the range of 17%–60 (Figure 2B). The distribution of species indicated that 48.07% of the unigenes had the highest similarity with sequences from the lepidopteran species *Bombyx mori*, followed by the lepidopteran species *Danaus plexippus* (25.57%), the coleopteran species *Tribolium castaneum* (2.95%), the lepidopteran species *Papilio xuthus* (1.76%), the lepidopteran species *H. armigera* (1.42%), and the hemipteran species *Acyrthosiphon pisum* (1.07%) (Figure 2C).

**FIGURE 3:**
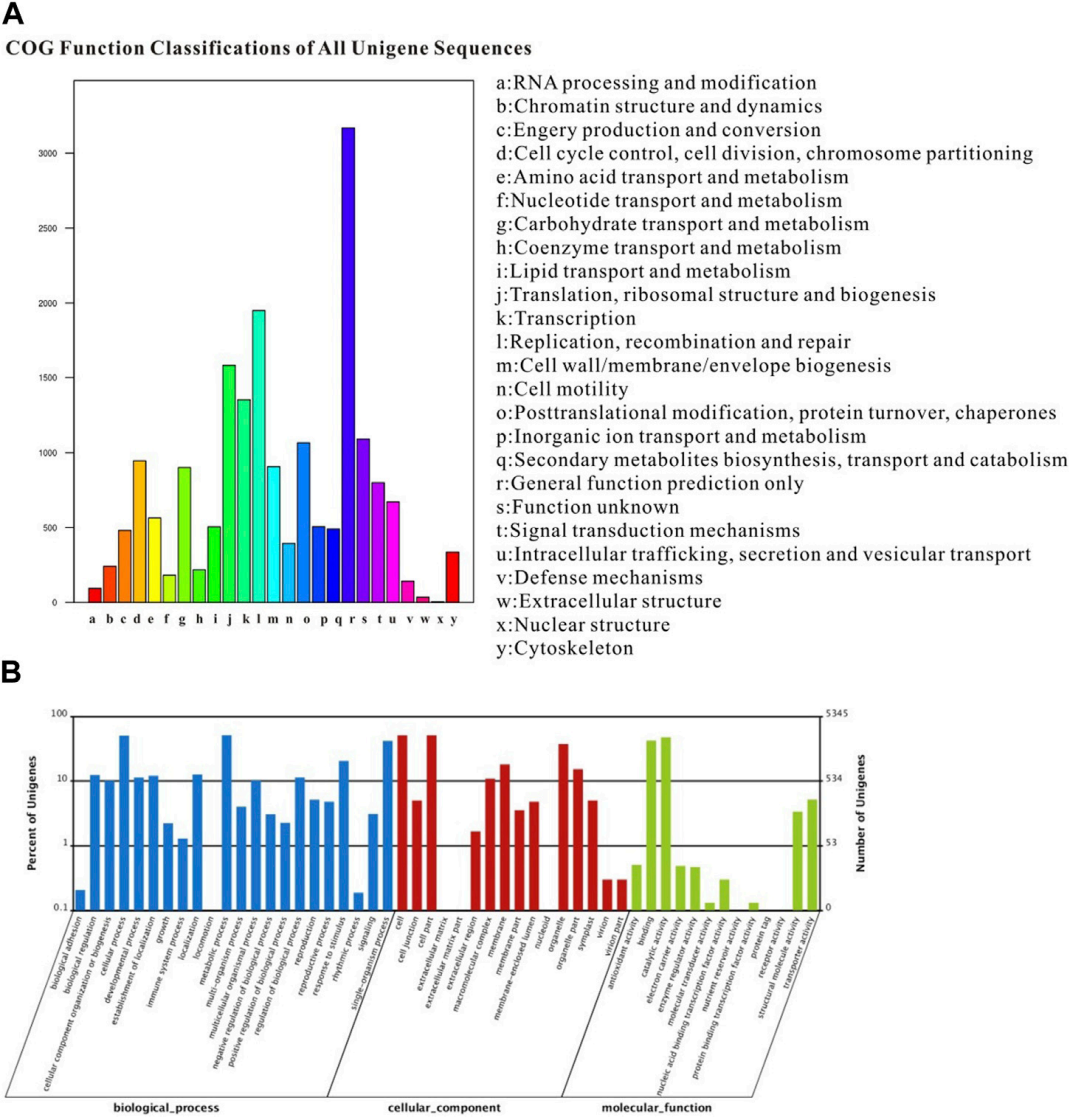
Annotation summary of *A. lepigone* proboscis unigenes by COG and GO classification. **(A)** COG categories of *A. lepigone* proboscis unigenes. **(B)** Gene ontology analysis of *A. lepigone* proboscis unigenes.

The COG classification assigned 18,619 unigenes to 25 COG categories, with the largest category being “general function prediction” (3168 genes; 17.01%). Additionally, 490 genes expressed in the proboscis were associated with the “secondary metabolite biosynthesis, transport, and catabolism” category (Figure 3A). The GO annotation analysis revealed that out of the 52,879 unigenes, a total of 30,717 were categorized under “biological process”, “cellular component” and “molecular function” encompassing 51 subcategories. The category of “biological process” exhibited the highest gene count in the proboscis, totaling 2729 genes. In the category of “cellular component,” category of “cell” and category of “cell part” were the most the most prevalent, with both having 2721 genes. Additionally, other genes were linked to subcategories such as “organelle,” “membrane,” “symplast,” and “extracellular matrix.” Regarding the “molecular function” terms, 2254 genes were categorized under “binding,” while 1093 genes were associated with “response to stimulus”. The remaining genes were assigned to subcategories subcategories including “metabolic process,” “cellular process,” “catalytic activity,” “biological regulation,” “signaling,” and “locomotion” (Figure 3B).

### 3.3 Odorant-binding protein family

The “classic” OBP group, which comprises the included general odorant-binding protein (GOBP) and pheromone-binding protein (PBP), was found to consist of six conserved cysteines. On the other hand, the “minus-C” group exhibited only four cysteines, while the “plus-C″ group contained more than six cysteines (Wang et al., 2017). In our investigation, we identified a total of 19 OBPs, including 1 PBP and 7 novel OBPs, in addition to 12 previously reported genes (Supplementary Table S2). Notably, none of these genes displayed any sexual specificity. The complete open reading frame (ORF) of AlepOBP19, which were previously lacking a complete sequence in previous studies, was also discovered.

Based on the presence of according to the conserved cysteines in the amino acid sequence, it can be determined that AlepOBP6, AlepOBP7, AlepOBP16, AlepOBP18, AlepOBP21, AlepOBP23, AlepGOBP1, and AlepGOBP2 belong to the Classic OBP subfamily, as they possess six conserved cysteines at the corresponding positions (Supplementary Figure S1A). On the other hand, AlepOBP24-30 and other genes, such as AlepPBP1, AlepOBP9, AlepOBP12, and AlepOBP19 are classified under the Minus-C OBP subfamily due to the absence of conserved cysteines in the C2 and C5 sites (Supplementary Figure S1B).

**FIGURE 4:**
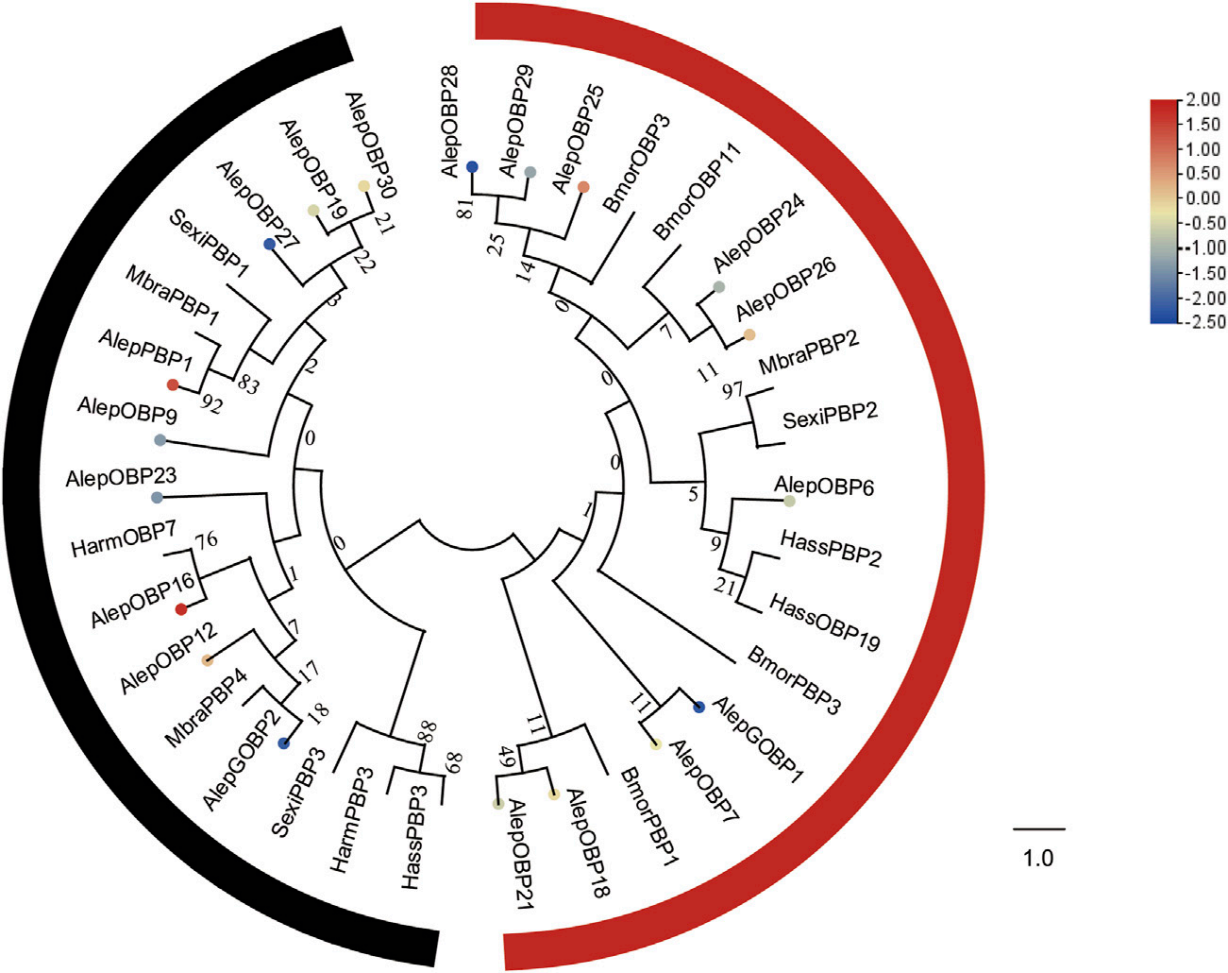
Candidate OBPs of *A. lepigone* proboscis. Phylogenetic tree of OBP genes from *A. lepigone* proboscis with relative lepidopteran OBPs (Alep, this study). The neighbour-joining tree was constructed using MEGA (1000 bootstrap replicates). The tree was rooted by the conservative OBPs. The OBPs identified in phylogenetic tree were shown in round dot with different colour, which the fold changes for AlepOBP genes from transcriptome sequence analysis from AMA/AFA comparison. Scale bar at the top right indicates the degree of expression, and the color range is from blue (low expression) to red (high expression).

To analyze the evolutionary relationships, a phylogenetic tree was constructed using the sequences of these seven newly identified OBPs (AlepOBP24, 25, 26, 27, 28, 29, and 30), along with 12 previously reported OBPs, and additional OBPs from *H. armigera*, *H. assulta*, *B. mori*, *Mamestra brassicae*, and *S. exigua*. AlepOBP28 and AlepOBP29, AlepOBP24 and AlepOBP26, AlepOBP18 and AlepOBP21, and AlepOBP19 and AlepOBP27 exhibited comparable genetic distances from each other. Conversely, AlepOBP6, AlepOBP7, AlepOBP18, AlepOBP21, AlepOBP24, AlepOBP25, AlepOBP26, AlepOBP28, and AlepOBP29 formed a distinct cluster. Additionally, AlepOBP12, AlepOBP16, AlepOBP19, AlepOBP23, AlepOBP27 and AlepOBP30 formed separate clusters. AlepGOBP1 and AlepGOBP2 demonstrated distinct genetic relationships, both of which clustered with AlepPBP1 and other lepidopteran insect genes, including BmorPBP1, SexiPBP1-3, MbraPBP1-2, and HarmPBP3 (Figure 4). Among the identified OBPs, five genes, namely, PBP1, OBP12, OBP16, OBP25, and OBP26, exhibited upregulation in the analysis of differentially expressed genes (DEGs). Furthermore, the analysis of DEGs detected 14 downregulated genes (Figure 4).

### 3.4 Chemosensory proteins

A total of twenty-two chemosensory proteins (CSPs) were annotated in *A. lepigone* through the analysis of proboscis transcriptome data. This includes the identification of two novel genes in addition to the previously reported twenty CSPs. Furthermore, this study also discovered the ORFs of AlepCSP5 and AlepCSP14, which were detected previously identified without complete sequences by Zhang et al. (2016). Among these genes, only AlepCSP2 exhibited female specificity, as indicated in Supplementary Table S2.

**FIGURE 5:**
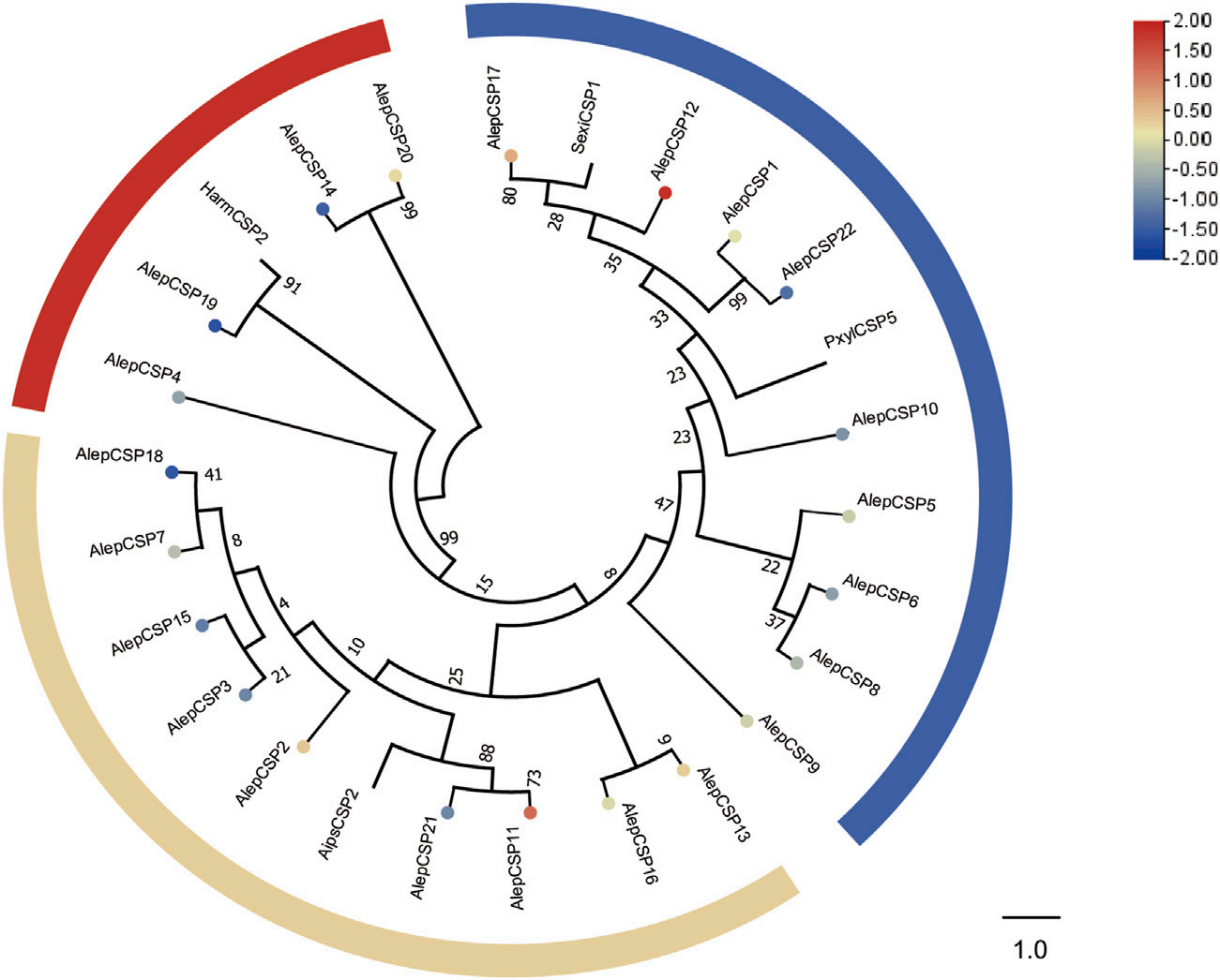
Candidate CSPs of *A. lepigone* proboscis. Phylogenetic tree of AlepCSP genes with relative lepidopteran CSPs. The neighbour-joining tree was constructed by MEGA (1000 bootstrap replicates). The tree was rooted by the conservative CSPs. The CSPs identified in phylogenetic tree were shown in round dot with different colour, which the fold changes for AlepCSP genes from transcriptome sequence analysis from AMA/AFA comparison. Scale bar at the top right indicates the degree of expression, and the color range is from blue (low expression) to red (high expression).

To establish the phylogenetic relationships, a tree was constructed using twenty-six AlepCSPs and four chemosensory proteins from other lepidopteran species, namely, HarmCSP2, AipsCSP2 (*Agrotis ipsilon* CSP2), PxylCSP5 (*Plutella xylostella*), and *MsexiCSP1* (*Manduca sexta*). Several conserved genes, such as AlepCSP19, exhibited a close relate to HarmCSP2, while both AlepCSP11 and AlepCSP21 showed a close relationship with AipsCSP2. Additionally, both AlepCSP1 and AlepCSP22 displayed a close relationship with PxylCSP5. Notably, AlepCSP5, AlepCSP6, AlepCSP8, AlepCSP9, AlepCSP10, AlepCSP12, AlepCSP17, and AlepCSP22 formed a cluster together. Furthermore, AlepCSP2, AlepCSP3, AlepCSP7, AlepCSP11, AlepCSP15, AlepCSP18, and AlepCSP21 formed another distinct cluster. The remaining AlepCSPs, including AlepCSP4, AlepCSP14, AlepCSP19, and AlepCSP20, formed a separate cluster (Figure 5). Among the 22 characterized CSPs, eight genes, namely, CSP1, CSP2, CSP11, CSP12, CSP13, CSP17, and CSP20 were found to be upregulated in the differential expression analysis. Conversely, 14 genes were identified as downregulated by DEG (Figure 5).

### 3.5 Chemosensory receptors

A total of eleven unigenes, which were associated with seven candidate ORs and four IRs, were identified through the analysis of unigene annotations. The identification of AlepOR38 was based on the full-length ORFs provided by Zhang et al. (2016), albeit with a partial fragment. AlepOR62 was discovered and characterized as a novel gene for the first time. Female specificity was observed in AlepOR3 and AlepOR4, while AlepIR41a showed male specificity (Supplementary Table S2).

**FIGURE 6:**
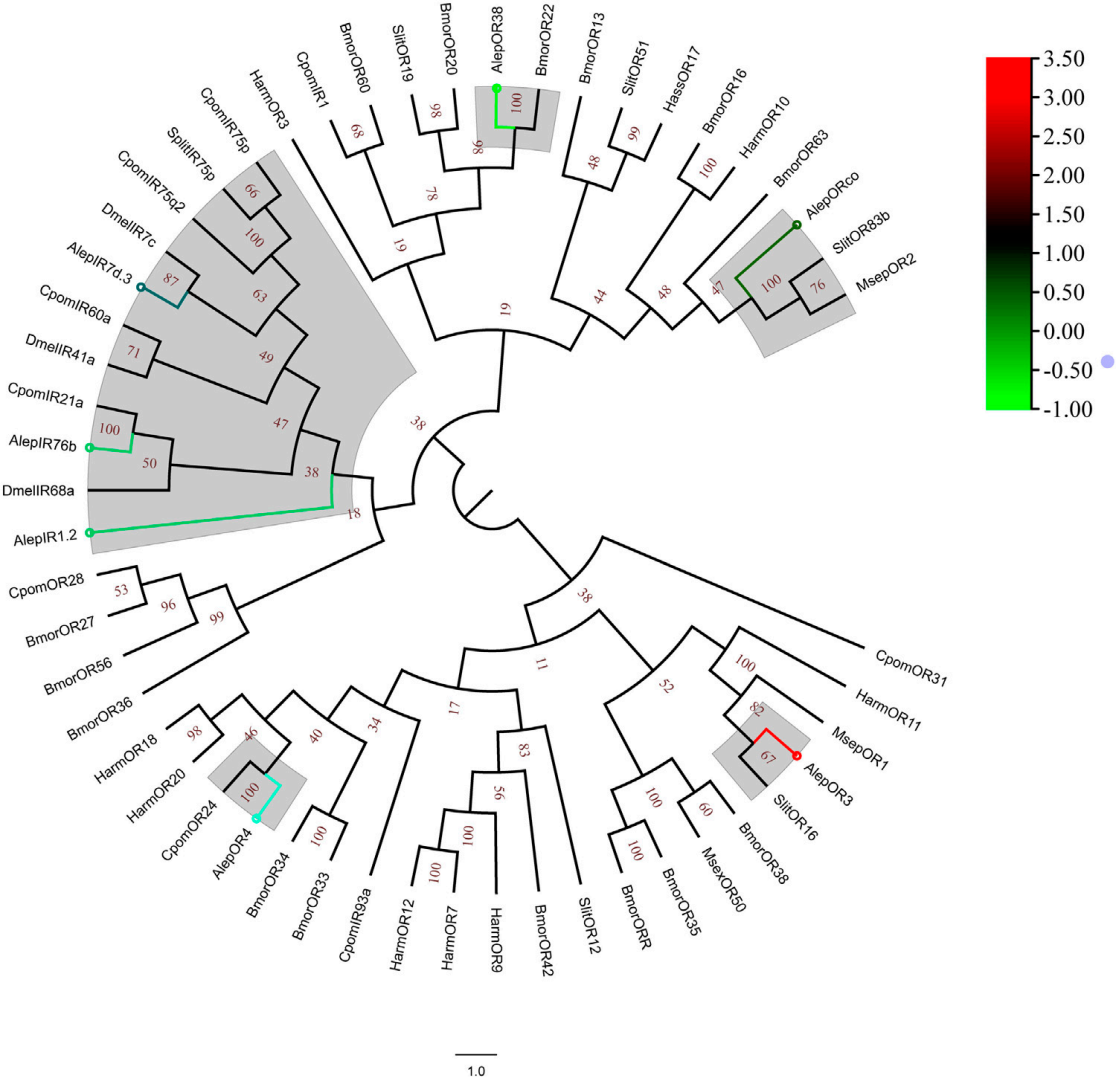
Candidate ORs of *A. lepigone* proboscis. Phylogenetic tree of AlepOR genes with relative lepidopteran ORs. The neighbour-joining tree was constructed by MEGA (1000 bootstrap replicates). The tree was rooted by the conservative ORs. The ORs identified in phylogenetic tree were shown in round dot with different colour, which the fold changes for AlepOR genes from transcriptome sequence analysis from AMA/AFA comparison. Scale bar at the top right indicates the degree of expression, and the color range is from blue (low expression) to red (high expression).

A phylogenetic tree was constructed using the amino acid sequences of AlepOR and AlepIR, as well as those from the nine others described lepidopteran insect species. AlepIR1.2, AlepIR76b, and AlepIR7d.3 exhibited a close genetic relationship, and they clustered with other lepidopteran OR and IR genes, such as *Cydia pomonella* and *S.exigua* formed a clade. AlepORco was closely related to OR2 from *Mythimna separata* (MsepOR2) and *Spodoptera litura* OR83b (SlitOR83b), and also clustered into a clade (Figure 6). Out of among the 11 identified ORs, five genes were found to be upregulated in the DEG analysis, while the remaining six genes showed downregulation (Figure 6).

### 3.6 Sensory neuron membrane proteins

**FIGURE 7:**
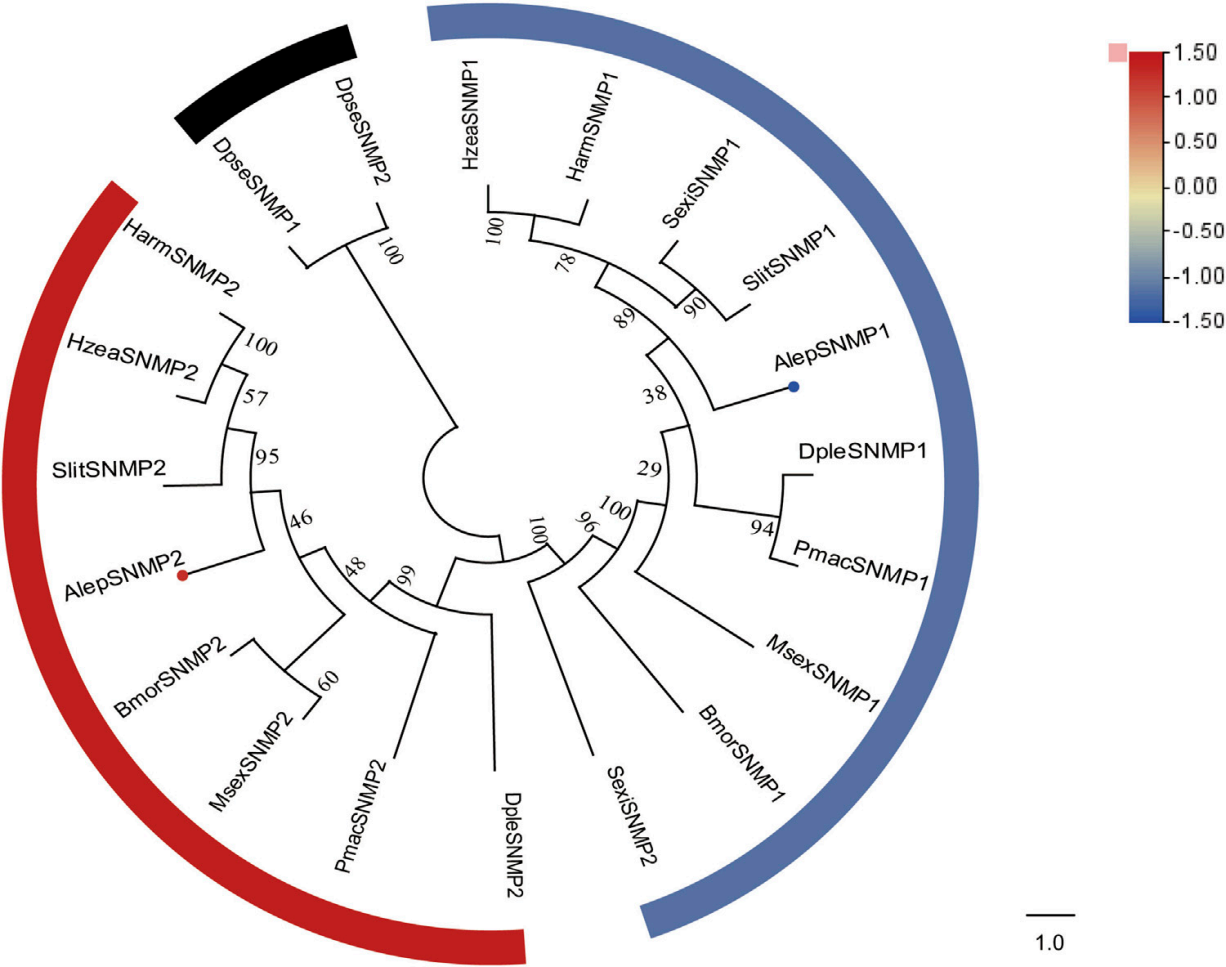
Candidate SNMPs of *A. lepigone* proboscis. Phylogenetic tree of SNMP genes with orthologous lepidopteran genes. The neighbour-joining tree was constructed by MEGA (1000 bootstrap replicates). The tree was rooted by the conservative SNMPs. The SNMPs identified in phylogenetic tree were shown in round dot with different colour, which the fold changes for SNMPs genes from transcriptome sequence analysis from AMA/AFA comparison. Scale bar at the top right indicates the degree of expression, and the color range is from blue (low expression) to red (high expression).

Two candidate sensory neuron membrane protein unigenes (SNMPs), namely, AlepSNMP1 and AlepSNMP2, were identified. AlepSNMP1 possessed a single transmembrane domain and displayed female specificity (Supplementary Table S2). The phylogenetic tree analysis revealed has a close relationship between AlepSNMP2 and SlitSNMP2 as they clustered together with HarmSNMP2 and formed a clade along with HzeaSNMP2. AlepSNMP1 exhibited a parallel genetic relationship with the AlepSNMP2 clade (Figure 7). The DEG analysis revealed that AlepSNMP1 exhibited upregulation, while AlepSNMP2 displayed downregulation (Figure 7).

### 3.7 Carboxylesterases

Based on the RNA-seq data, a total of five putative carboxylesterase genes were identified *A. lepigone* (AlepCXEs). The genes AlepCXE14, AlepCXE18, AlepCXE20, AlepCXE23, and AlepCXE28 were detected following the methodology described by Zhang et al. (2017a). All annotated genes possessed complete ORF, except for AlepCXE28 (Supplementary Table S2).

**FIGURE 8:**
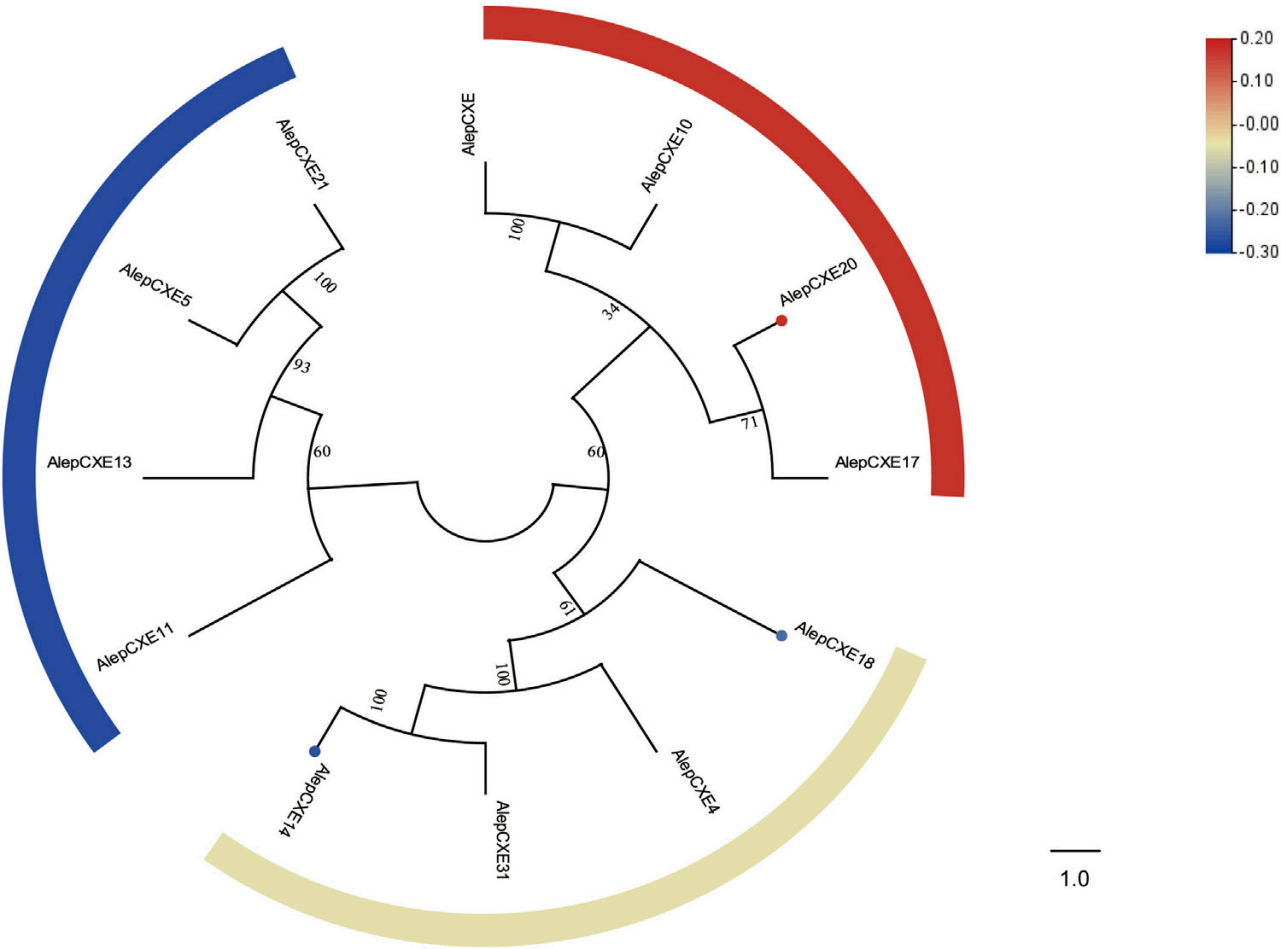
Candidate CXEs of *A. lepigone* proboscis. Phylogenetic tree of AlepCXEs genes with known lepidopteran CXEs. The neighbour-joining tree was constructed by MEGA (1000 bootstrap replicates). The tree was rooted by the conservative SNMPs. The CXEs identified in phylogenetic tree were shown in round dot with different colour, which the fold changes for CXEs genes from transcriptome sequence analysis from AMA/AFA comparison. Scale bar at the top right indicates the degree of expression, and the color range is from blue (low expression) to red (high expression).

Through phylogenetic tree analysis, it was observed that AlepCXE14 shared a close genetic relationship with AlepCXE18, both of which formed a clade along with AlepCXE4 and AlepCXE31. Additionally, AlepCXE20 together with AlepCXE17 (Figure 8). The DEG analysis further indicated that AlepCXE14 and AlepCXE18 exhibited upregulation, while the expression of other genes such as AlepCXE20, AlepCXE23 and AlepCXE28 were all downregulated (Figure 8).

### 3.8 Cytochrome P450s

The COG annotation of the transcriptomes revealed the enrichment of proteins involved in secondary metabolite biosynthesis, transport, catabolism, and lipid transport and metabolism. Subsequent analysis of these transcripts led to the identification of five potential candidate CYPs (Supplementary Table S2). Additionally, AlepCYP2 was predominantly male-biased (Supplementary Table S2).

**FIGURE 9:**
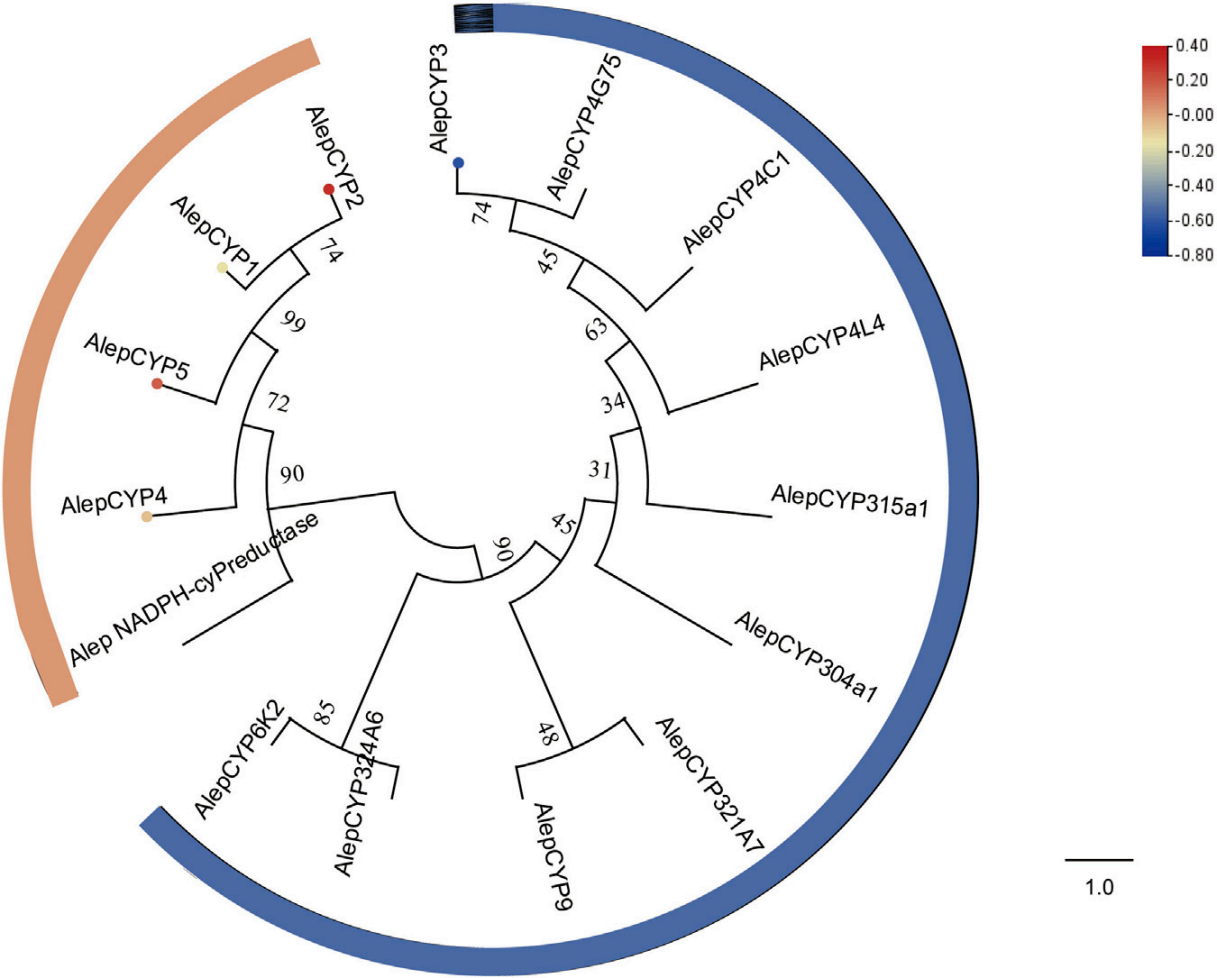
Candidate CYPs of *A. lepigone* proboscis. Phylogenetic tree of AlepCYPs and with known lepidopteran CYPs. The neighbour-joining tree was constructed by MEGA (1000 bootstrap replicates). The tree was rooted by the conservative CYPs. The CYPs identified in phylogenetic tree were shown in round dot with different colour, which the fold changes for CYPs genes from transcriptome sequence analysis from AMA/AFA comparison. Scale bar at the top right indicates the degree of expression, and the color range is from blue (low expression) to red (high expression).

The phylogenetic tree analysis demonstrated that AlepCYP1, AlepCYP2, AlepCYP4, and AlepCYP5 were genetically equidistant and closely related to AlepNADPH-CYP reductase. In contrast, AlepCYP3 exhibited the greatest genetic distance (Figure 9). DEG analysis indicated that AlepCYP2 and AlepCYP5 were upregulated, while other genes were downregulated (Figure 9).

### 3.9 Sex- and tissue-specific candidate gene expression profiles validated using qRT-PCR

**FIGURE 10:**
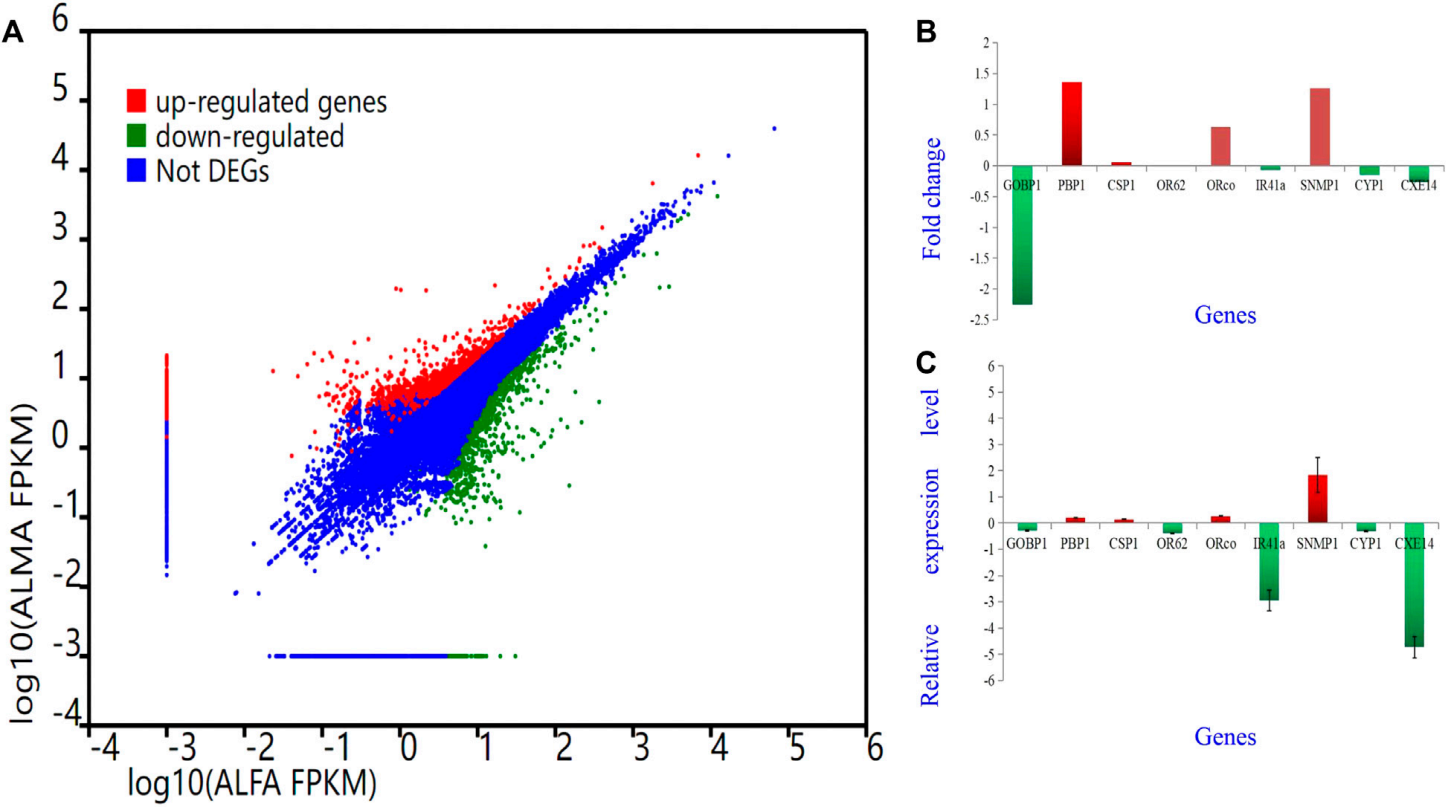
Different Gene expression analysis. Unigenes between proboscis of male adults and female adults of A. lepigone comparisons. **(A)** Up-regulated (red) and down-regulated (green) unigenes were quantified using the tag-based digital gene expression (IDEG6) tool. **(B)** The fold changes of selected eight unigenes from transcriptome sequence analysis between male and female adults’ comparison. **(C)** The relative expression levels of selected eight unigenes by qRT-PCR in AMA compared with AFA.

To validate the findings obtained from deep sequencing, a random selection of genes, namely, AlepGOBP1, AlepPBP1, AlepCSP1, AlepOR62, AlepORco, AlepIR41a, AlepSNMP1, AlepCYP1, and AlepCXE14, were quantified using gene-specific primers through qRT-PCR (Supplementary Table S1). For instance, based on transcriptome analysis, AlepSNMP1 (CL452.Contig2_All) exhibited downregulation (log_2_Ratio [ALMA/ALFA] = 1.26) and demonstrated a fold change of (1.83 ± 0.66) in the qRT-PCR experiment. The qRT-PCR results exhibited similar trends of upregulation or downregulation as observed in the deep sequencing analysis (Figures 10A–C).

**FIGURE 11:**
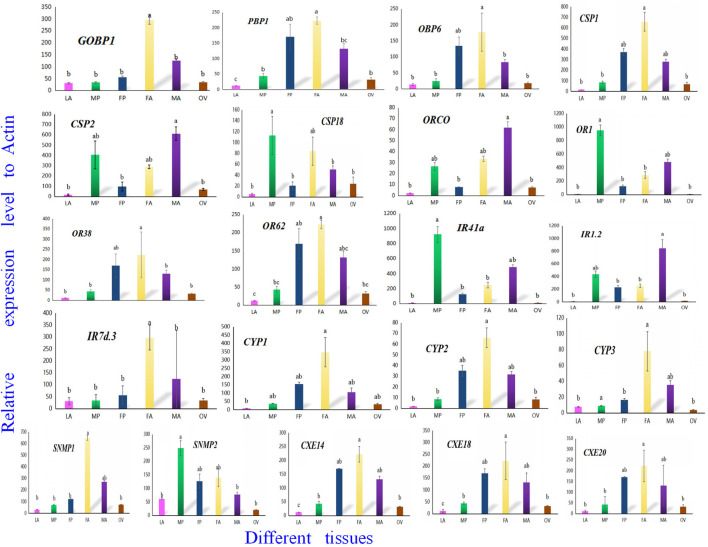
Twenty-one candidate genes were selected to validate the tissue distribution *in vitro* by qRT-PCR. qRT-PCR analysis of genes in sixth instar larval head oral appendages (maxilla, labium and antenna) (LA), male adult proboscis (MP), female adult proboscis (FP), male adult antennae (MA), female adult antennae (FA), pheromone gland-ovipositor of female adults (OV), respectively. Data was mean ± SE. Different letters on the bar indicated significant differences (one-way ANOVA analysis and Tukey’s multiple comparison, n = 3, *p* < 0.05). The housekeeping gene (β-actin) was used for normalization in all qPCR analyses.

To confirm the involvement of genes associated with chemosensory and metabolic processes in *A. lepigone* development, expression profiles in different tissues were constructed from LA, MP, FP, FA, MA, and OV by randomly selecting 21 genes, including AlepGOBP1, AlepPBP1, AlepCSP1, AlepOR62, AlepORco, AlepIR41a, AlepSNMP1, AlepCYP1, and AlepCXE14 using qRT-PCR. The results found that these 21 genes exhibited similar expression patterns in the proboscis tissues of both male and female adults Figure 11. Within the OBP family, AlepGOBP1 and AlepOBP6 were ubiquitously expressed in all larval and adult tissues, albeit at significantly higher levels. Specifically, the expression level of APBP1 was found to be the highest in female adults. However, no significant differences were observed between female adult and female pupae, while the expression level in FA was significantly higher than that in male adults. Among the chemosensory proteins (CSPs), AlepCSP1exhibited the highest expression in FA, AlepCSP2 exhibited the highest expression in MA, and AlepCSP18 exhibited the highest expression in MP. Notably, all three genes displayed the lowest expression in the larval stage (LA). Furthermore, among the chemosensory receptor genes, AlepORco and AlepIR1.2 exhibited the highest expression in MA. The expression levels of AlepOR1 and AlepIR41a found to be highest in the male proboscis (MP). Conversely, For the protein expression of AlepOR38, AlepOR62, and AlepIR7d.3 was significantly higher in the male antennae (MA) compared to other tissues. In the case of CYPs, all three genes exhibited their highest expression levels in the female antennae (FA). Among the SNMPs, AlepSNMP1 displayed the highest expression levels in the female antennae (FA), while AlepSNMP2 predominantly expressed in MP. As for CXEs, the expression levels of all three genes were highest in FA and lowest in the pheromone gland-ovipositor of female adults (OV) (Figure 11).

## 4 Discussion

Migratory moths exhibit a limited capacity to lay eggs, primarily non-viable ones, when lacking sufficient nutrition during the adult stage (Han and Gatehouse, 1991). The presence of adequate sources of nectar along their migratory route is a prerequisite for these moths to successfully reach their intended habitats and facilitate reproductive processes (Guo et al., 2018b). Initially, research predominantly focused on the host recognition or mating behavior of moth antennae, which are considered the primary olfactory organs (Christensen and Hildebrand, 1987; Chang et al., 2017; Guo et al., 2021). However, it is important to acknowledge that the mouthparts of these moths also play significant roles in ecological behavior, particularly in nectar foraging. This behavior involves the detection and mediation of floral scents through the olfactory receptors located at the tip of a moth’s proboscis (Haverkamp et al., 2016; Guo et al., 2022). The morphology of the proboscis in *A. lepigone* has been extensively examined in this study (Hu et al., 2021). Specifically, we conducted a thorough investigation of the entire structure of the proboscis in adult *A. lepigone* using electron microscopy. Our observations through electron microscopy unveiled a diverse array of sensilla present on the proboscis, suggesting the existence of a complex and comprehensive chemosensory and metabolic system within the proboscis of *A. lepigone*.

A number of previous studies have been used to study the role of chemosensory genes based on RNA sequencing (RNA-Seq) data obtained from *A. lepigone* at various developmental stages (Li et al., 2013; Zhang et al., 2016), the precise molecular mechanisms underlying chemoreception and metabolism in the proboscis remain elusive (Zhang et al., 2017b). According to our transcriptome data, we obtained 61,121 and 61,156 unigenes from the proboscis of male and female adults of *A. lepigone*, respectively. Additionally, 12,137 unigenes were found to be longer than 1, 000 nucleotides, indicating the reliability of the deep sequencing for further investigations. Analysis of homology showed that *B. mori* possessed the highest proportion of homologous genes (48.07%). The current focus on moth olfaction research has been primarily limited to domesticated silk moths, largely due to the availability of genomic data. Our deep sequencing study of *A. lepigone*, which exhibits a more complex behavior than *B. mori*, is an important advance in the study of olfaction in non-model insects.

A total of fifty-two chemosensory genes were characterized, including 19 OBPs, 22 CSPs, one Orco, six ORs, four IRs, and two SNMPs were identified in this research. Additionally, out of all the chemosensory genes above, seven OBPs, two CSPs, and one OR were identified as novel gene compared with previous report (Zhang et al., 2016; Zhang et al., 2017b). In this study, we focused on candidate gene family members of the olfactory and gustatory organs, including olfactory gene-encoding protein families and receptor families. Furthermore, 14 metabolism-related genes, including nine CYPs and five CXEs, have also been identified.

Notably, the expression patterns of most chemosensory gene in both adult male and female *A. lepigone* are similar to those in other lepidopteran insects, such as *Spodoptera littoralis* (Bengtsson et al., 2012) and *H. armigera* (Fox et al., 2001). This indicates that odor-coding capacity is similar between two sexes. In lepidopteran insects, the mRNA expression level of seven ORs and 13 ORs in *Chilo suppressalis* (Glaser et al., 2013) and *H. armigera* (Guo et al., 2018b) were reported, respectively. In our study, we firstly identified and detected the spatial gene transcription incuding13 chemosensory genes using qRT-PCR methods. These gene expression profiles in *A. lepigone* were consistent with those of other moths (Glaser et al., 2013; Guo et al., 2018a; Guo et al., 2018b). We have also identified several highly expressed metabolic genes that are crucial for the adult life-stage of *A. lepigone*, such as CYPs and five CXEs. Furthermore, we have found some genes that may potentially be relevant to key behaviors, such as pesticide resistance and detoxification metabolism. The analysis of tissue distribution has revealed that the expression patterns of three AlepCXEs (AlepCXE14, AlepCXE18, and AlepCXE28) were significantly higher in the antennae of female adults compared to other tissues. Moreover, our findings indicate that the expression of AlepCXEs is predominantly observed in the male antennae, which differs from the results reported in previous study (Zhang et al., 2017a).

In our study, the annotated genes were mainly expressed in the proboscis or antennae of *A. lepigone*. In addition, the OR1 and IR1.2 were also enriched in the *A. lepigone* pheromone gland-ovipositor (OV) of female adults. Normally, odor signals are primarily detected by cephalic organs, including the antennae, proboscises, maxillary palps, and abdominal organs comprised of wings and legs. Similar results have also been reported for *Spodoptera frugiperda* (Sun et al., 2022).

To a certain degree, the emergence of *A. lepigone* as a prominent pest in summer maize in China can be attributed to the adoption of a novel cultivation technique and the implementation of sustainable practices for managing crop residues. However, the precise mechanism through which these pests are drawn to this complex environment remains uncertain. It is widely acknowledged that the majority of insects rely on volatile odors to guide their behaviors related to food selection, mating, egg laying, and repulsion, utilizing their olfactory system by teracting with various specific pheromone constituents (Venthur et al., 2019). In this study, we conducted an analysis of the proboscis transcriptome of *A. lepigone* in order to identify gene families associated with olfaction. This analysis has provided us with additional genetic information that can be utilized in the development of targeted control genes. The exploration and characterization of crucial genes related to olfaction and metabolism in *A. lepigone* may may offer valuable insights for behavioral research and population monitoring. Furthermore, these newly discovered genes have the potential to serve as targets for enhancing future pest control techniques, including mating disruption and the use of food attractants. In addition, it would be advantageous to ascertain the distribution of chemosensory gene expression and gain insights into the functionalities of these genes, thereby establishing a novel theoretical framework for the advancement of eco-friendly pesticides and efficient pest management strategies in the future.

## Data Availability

The datasets presented in this study can be found in online repositories. The names of the repository/repositories and accession number(s) can be found below: https://ngdc.cncb.ac.cn/gsa, CRA012317.
