# Supplementary material for: Molecule characterization of chemosensory and metabolism-related genes in the proboscis of Athetis lepigone
Source: Front Physiol. 2023 Dec 22;14:1287353. doi: 10.3389/fphys.2023.1287353 (PMC10766847; doi:10.3389/fphys.2023.1287353)
Supplement: Supplementary file 1 [file Table1.docx]

**Table S1**. **Primers for real-time quantitative-PCR of candidate *OBP*s,*CSPs*,*ORs* *IRs,CYPs,SNMPs* and *CXEs*.**

| **Gene name** | **Forward primer (5' to 3')** | **Reverse primer (5' to 3')** |
| --- | --- | --- |
| ***OBPs*** |  |  |
| GOBP1 | CTGACGGAGGAGAAGATG | GTAAGCAGGTTGAAGTAGTTG |
| PBP1 | ATCCAAGATGGCAGTTCA | AACCTTCGTAAACCCTGTA |
| OBP6 | GAGATGGACGAGGACATG | CACTTGATGTAGCACTTGAG |
| ***CSPs*** |  |  |
| CSP1 | GCCGTATTCCTTCTCTGT | TTCAGTTCCTTGCCATCA |
| CSP2 | AGACATCTTGCGGTAAGT | CTTCAGGGTCGTTCTTCT |
| CSP18 | CGACCTGGATACTCTCAA | AGAAGGAACTTGTGGAAGA |
| ***ORs*** |  |  |
| AlepORco | GAATAAGGAAACGAACGAGACG | ACCCAGTACTTGATGGCAGACC |
| AlepOR1 | ATAAATGGAAAACGTGGCACG | ACCAGCAGCAAACAAACAGC |
| AlepOR38 | ATGCCGAGCTACATGTGCAC | GGATTGACGCAGCATATTGAAC |
| AlepOR62 | TTCTGCTGTGCTGGTATGGA | CGTAGATGCCCTTTTGAGCC |
| ***IRs*** |  |  |
| IR41a | AGAAGGTGACTAAGGCCAAACG | GGACAGCACAACTAGCAGGAAGA |
| IR1.2 | AGATGGATTGGGGAATACGAGA | GGCAAATAGAAGCAACGACAGA |
| IR7d.3 | TGCCATCTCATACCTATCACACA | CAATAACCACTTCAACCCCACC |
| ***CYPs*** |  |  |
| CYP1 | ATTTGAAGACGAGCGAACCG | GTCTTCACAGGGACGAAAAGT |
| CYP2 | GCCCGTAGATCACTGATGGT | ATTTGTGGCGAAGGATCTGC |
| CYP3 | CTCAGAACTTGTGGCTGGTG | AAGAGTCAGGGTGCCACTAC |
| ***SNMPs*** |  |  |
| SNMP1 | AGCTCCAATGTATGCCTCCA | TCCTTTGTTTCGCCACCATC |
| SNMP2 | GTGCCAATCCGAACAACAGA | AGTTCTTCGGAGCCAAGGAA |
| ***CXEs*** |  |  |
| CXE14 | TGATGATTGGCTAGAAGTGA | GGCGTAGGGTATGTTGTA |
| CXE18 | TTGGAATACCTTACGCTACT | ATGAAGATTGTGGACATTGG |
| CXE20 | GAGTAATCCTAGTCACCTTCA | TCCGAATGCTCTGATGTT |
| **Reference gene** |  |  |
| ***Actin*** | CACTCACTCGTGGCAACAAT | CGCCTGACGTTCATCATACT |
