# Supplementary figures and images for: Molecule characterization of chemosensory and metabolism-related genes in the proboscis of Athetis lepigone
Source: Front Physiol. 2023 Dec 22;14:1287353. doi: 10.3389/fphys.2023.1287353 (PMC10766847; doi:10.3389/fphys.2023.1287353)

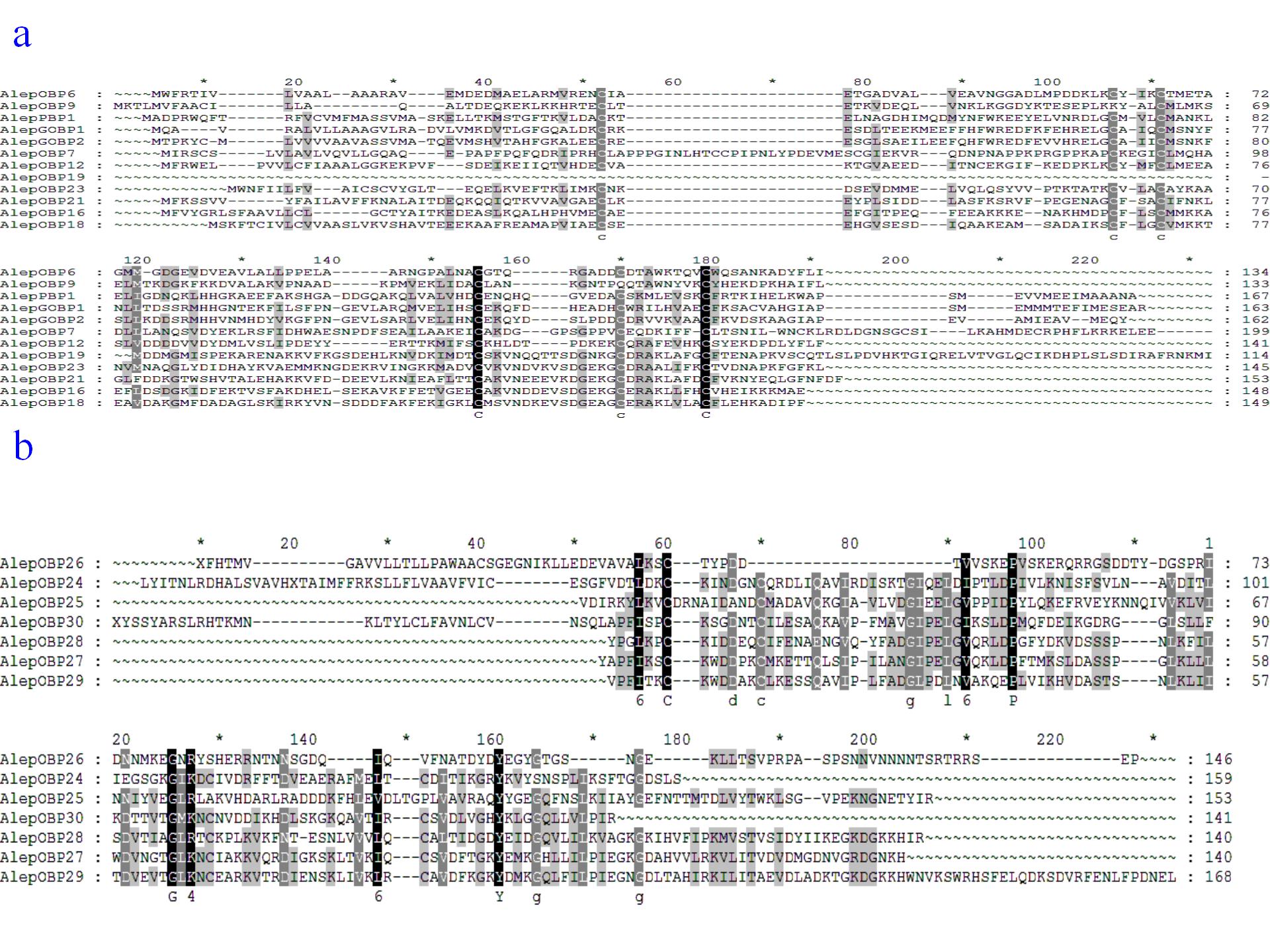

Supplement: Supplementary file 2 [file Image1.jpg]
