# Supplementary material for: Molecule characterization of chemosensory and metabolism-related genes in the proboscis of Athetis lepigone
Source: Front Physiol. 2023 Dec 22;14:1287353. doi: 10.3389/fphys.2023.1287353 (PMC10766847; doi:10.3389/fphys.2023.1287353)
Supplement: Supplementary file 3 [file DataSheet1.docx]

File S1 The amino acid Sequence of ***A. lepigone*** from proboscis and other insects from NCBI database.

**a. OBPs used in fig 4**

>AlepGOBP1

MQAVRALVLLAAAGVLRADVLVMKDVTLGFGQALDKCRKESDLTEEKMEEFFHFWREDFKFEHRELGCAIQCMSNYFNLLTDSSRMHHGNTEKFILSFPNGEVLARQMVELIHSCEKQFDHEADHCWRILHVAECFKSACVAHGIAPSMEMMMTEFIMESEAR

>AlepGOBP2

MTPKYCMLVVVVAAVASSVMATQEVMSHVTAHFGKALEECREESGLSAEILEEFQHFWREDFEVVHRELGCAIICMSNKFSLLKDDSRMHHVNMHDYVKGFPNGEVLSARLVELIHNCEKQYDSLPDDCDRVVKVAACFKVDSKAAGIAPEVAMIEAVMEQY

>AlepPBP1

MADPRWQFTRFVCVMFMASSVMASKELLTKMSTGFTKVLDACKTELNAGDHIMQDMYNFWKEEYELVNRDLGCMVLCMANKLELIGDNQKLHHGKAEEFAKSHGADDGQAKQLVALVHDCENQHQGVEDACSKMLEVSKCFRTKIHELKWAPSMEVVMEEIMAAANA

>AlepOBP6

MWFRTIVLVAALAAARAVEMDEDMAELARMVRENCIAETGADVALVEAVNGGADLMPDDKLKCYIKCTMETAGMMGDGEVDVEAVLALLPPELAARNGPALNACGTQRGADDCDTAWKTQVCWQSANKADYFLI

>AlepOBP7

MIRSCSLVLAVLVQVLLGQAQEPAPFPQFQDRIPRHCLAPPPGINLHTCCPIPNLYPDEVMESCGIEKVRQDNPNAPPKPRGPPKAPCKEGICLMQHADLLLANQSVDYEKLRSFIDHWAESNPDFSEAILAAKEICAKDGGPSGPPVCEQDKIFFCLTSNILWNCKLRDLDGNSGCSILKAHMDECRPHFLKRKELEEQNGQ

>AlepOBP9

MKTLMVFAACILLAQALTDEQKEKLKKHRTECLTETKVDEQLVNKLKGGDYKTESEPLKKYALCMLMKSELMTKDGKFKKDVALAKVPNAADKPMVEKLIDACLANKGNTPQQTAWNYVKCYHEKDPKHAIFL

>AlepOBP12

MFRWELPVVLCFIAAALGGKEKPVFSDEIKEIIQTVHDECVAKTGVAEEDITNCEKGIFKEDPKLKCYMFCLMEEASLVDDDDVVDYDMLVSLIPDEYYERTTKMIFSCKHLDTPDKEKCQRAFEVHKCSYEKDPDLYFLF

>AlepOBP16

MFVYGRLSFAAVLLCLGCTYAITKEDEASLKQALHPHVMECAEEFGITPEQFEEAKKKENAKHMDPCFLSCMMKKAEFLDSDGKIDFEKTVSFAKDHELSEKAVKFFETVGEECAKVNDDEVSDGEKGCERAKLLFHCVHEIKKKMAE

>AlepOBP18

MSKFTCIVLCVVAASLVKVSHAVTEEEKAAFREAMAPVIAECSEEHGVSESDIQAAKEAMSADAIKSCFLGCVMKKTEAVDAKGMFDADAGLSKIRKYVNSDDDFAKFEKIGKLCMSVNDKEVSDGEAGCERAKLVLACFLEHKADIPF

>AlepOBP19

MDDMGMISPEKARENAKKVFKGSDEHLKNVDKIMDTCSKVNQQTTSDGNKGCDRAKLAFGCFTENAPKVSCQTLSLPDVHKTGIQRELVTVGLQCIKDHPLSLSDIRAFRNKMIPNGKDPKCFVACLFKKIGV

>AlepOBP21

MFKSSVVYFAILAVFFKNALAITDEQKQQIQTKVVAVGAECLKEYPLSIDDLASFKSRVFPEGENAGCFSACIFNKLGLFDDKGTWSHVTALEHAKKVFDDEEVLKNIEAFLTTCAKVNEEEVKDGEKGCDRAKLAFDCFVKNYEQLGFNFDF

>AlepOBP23

MWNFIILFVAICSCVYGLTEQELKVEFTKLIMKCNKDSEVDMMELVQLQSYVVPTKTATKCVLACAYKAANVMNAQGLYDIDHAYKVAEMMKNGDEKRVINGKKMADVCVKVNDVKVSDGEKGCDRAALIFKCTVDNAPKFGFKL

>AlepOBP24

LYITNLRDHALSVAVHXTAIMFFRKSLLFLVAAVFVICESGFVDTLDKCKINDGNCQRDLIQAVIRDISKTGIQELDIPTLDPIVLKNISFSVLNAVDITLIEGSGKGIKDCIVDRFFTDVEAERAFMELTCDITIKGRYKVYSNSPLIKSFTGGDSLS

>AlepOBP25

VDIRKYLKVCDRNAIDANDCMADAVQKGIAVLVDGIEELGVPPIDPYLQKEFRVEYKNNQIVVKLVINNIYVEGLRLAKVHDARLRADDDKFHLEVDLTGPLVAVRAQYYGEGQFNSLKIIAYGEFNTTMTDLVYTWKLSGVPEKNGNETYIR

>AlepOBP26

XFHTMVGAVVLLTLLPAWAACSGEGNIKLLEDEVAVALKSCTYPDDTVVSKEPVSKERQRRGSDDTYDGSPRIDNNMKEGNRYSHERRNTNNSGDQIQVFNATDYDYEGYGTGSNGEKLLTSVPRPASPSNNVNNNNTSRTRRSEP

>AlepOBP27

YAPFIKSCKWDDPKCMKETTQLSIPILANGIPELGVQKLDPFTMKSLDASSPGLKLLLWDVNGTGLKNCIAKKVQRDIGKSKLTVKIQCSVDFTGKYEMKGHLLILPIEGKGDAHVVLRKVLITVDVDMGDNVGRDGNKH

>AlepOBP28

YPGLKPCKIDDEQCIFENAENGVQYFADGIPELGVQRLDPGFYDKVDSSSPNLKFILSDVTIAGLRTCKPLKVKFNTESNLVVVLQCALTIDGDYEIDGQVLILKVAGKGKIHVFIPKMVSTVSIDYIIKEGKDGKKHIR

>AlepOBP29

VPFITKCKWDDAKCLKESSQAVIPLFADGLPDLNVAKQEPLVIKHVDASTSNLKLIITDVEVTGLKNCEARKVTRDIENSKLIVKLRCAVDFKGKYDMKGQLFILPIEGNGDLTAHIRKILITAEVDLADKTGKDGKKHWNVKSWRHSFELQDKSDVRFENLFPDNEL

>AlepOBP30

XYSSYARSLRHTKMNKLTYLCLFAVNLCVNSQLAPFISPCKSGDNTCILESAQKAVPFMAVGIPELGIKSLDPMQFDEIKGDRGGLSLLFKDTTVTGMKNCNVDDIKHDLSKGKQAVTIRCSVDLVGHYKLGGQLLVLPIR

>MbraPBP4

MSKFGCFVLCVVAASISRVYASEEDKAAFREAIKPIIEECSKEHGVSTDDIQSAKTAGSAEGIKPCFLGCVYKKAEVFNAKGEYDVDNALLKLKKFVPDEAKYAKFAEIGKKCASVNDKAVSDGEAGCERGALLTACFLENRGEILI

>MbraPBP2

MALHRSTTMSVRLALVAIASLFITVECSQEIMKNLSMNFAKPLEDCKKEMDLPDSVTTDFYNFWKEGYEFTNRQTGCAILCLSSKLELLDQELKLHHGKAQEFAQKHGADEAMAKQLVDLIHGCTQSTPDVAADPCMKALNVAMCSKTKVHELNWAPSVELIVGEVLAEV

>MbraPBP1

MTYSKWRLSCLVCVIFVASSVMASKELITKMSSGFTKVVDQCKNELNVGEHIMQDMYNFWREEYELLNRDLGCMVMCMANKLDLIGEDQKMHHGKAADFAKSHGADDDQAKQLVGIVHDCENTHQGVEDACSRALEVAKCFRSKMHELKWAPSMEVIMEEIMTAV

>BmorPBP3

MARYNIVVAVLVLGVVGARGSSEAMRHIATGFIRVLDECKQELGLTDHILTDMYHFWKLDYSMMTRETGCAIICMSKKLDLIDGDGKLHHGNAQAYALKHGAATEVAAKLVEVIHGCEKLHESIDDQCSRVLEVAKCFRTGVHELHWAPKLDVIVGEVMTEI

>BmorPBP1

MSIQGQIALALMVYMAVGSVDASQEVMKNLSLNFGKALDECKKEVGKMTLTDAINEDFYNFWKEGYEIKNRETGCAIMCLSTKLNMLDPEGNLHHGNAMEFAKKHGADETMAQQLIDIVHGCEKSTPANDDLCIWTLGVATCFKAEIHKLNWAPSMDVAVGEILAEV

>HassPBP2

MMGSAMSSKELLTKMSEGFTKVVDACKTQLNVGDHITQDMYNFWREEYQLVNRDLGCMIMCMVAKLDLIGDDQKMHHGKAEEFAKSHGADDVLAKQLVSLIHSCETQHQAIEDHCSRVLEIAKCFRTKIHELKWAPSMEVVMEEIMTAA

>HassPBP3

MGSRHVFFAFAVLAVSVRKAEPSKDAMQYITSGFVKVLEECKHELNLNEQILADLFHFWKLEYSLLGRDTGCAIICMSKKLDLLDANGRMHHGNAAEFAKKHGAGDEVASKIVTIIHECEKKHEQDGDECLRVLEVAK

>HarmPBP3

MGSRHVFFALVVLAVSVRKAEPSKDAMQYITSGFVKVLEECKHELDLNEQILADLFHFWKLEYSLLGRDTGCAIICMSKKLDLLDANGRMHHGNAAEFAKKHGAGDEVASKIVTIIHECEKKHEQDGDECLRVLEVAKCFRTGIHELDWQPKVEVIVSEVLTEI

>SexiPBP1

MAGAKWRFVCVVFALYLTSAALGSQELMMKMTKGFTKVVDECKAELNAGEHIMQDMYNYWREDYQLINRDLGCMILCMAKKLDLMEDQKMHHGKTEEFAKSHGADDEVAKKLVSIIHECEQQHAGIADDCMRVLEISKCFRTKIHELKWAPNMEVIMEEVMTAV

>SexiPBP2

MAFCRSATMSVRVALVVAASMLVVVQASQDVMKNLAINFAKPLDDCKKEMDLPDSVTTDFYNFWKEGYELTNRQTGCAILCLSSKLEILDQELNLHHGRAQEFAMKHGADETMAKQIVDMIHTCAQSTPDVAADPCMKTLNVAKCFKLKIHELNWAPSMELIVGEVLAEV

>SexiPBP3

MGSHNVFVALVLLAVGMRVAEPSKDAMKYITSGFVKVLEECKQELNMNDHIIADLFHFWKLEYALLSRDTGCVIICMSKKLDLLDANGRMHHGNAQEFAKRHGAGDDVASKIVQIIHDCEKKHERDDDECLRVLEVAKCFRTGIHDLDWQPKVEVIVSEVLTEI

>BmorOBP3

MHVTYFVTFLSVICIVNSTPPSFVNRCKYGDSECTKESTVVAIPIFAAGLPEYGVEKLDPVTFNKVDASSPNLKFILTDVEVTGLSGCKPKQIQHGSKLELKILCQAKLNGNYELNGQVLVLPIKGKGKIHVDLKTTQINVDANYEEKLGDDGKKHWHITKWSYTFELQDKSDVVFENLFDGNEVLGQAARELIANNGNDIIKEIGSPMIKAAVARVMKNIERFFKAIPVEDLILN

>BmorOBP11

MMGYACVFVILAVLQAISAEDPPGLPPFLKDAPEKCRSPPRVKNPNECCISEPFFKEADFIECGIEKPGSERGPPDCSKQNCLLKKYNLLKNDETPDIEAIKSLLDKYIEKNPSFKSSVEKAKECLREDLPGPPQICLANRMTLCIGTVLLMECPDEKWNTTDDCKAFKDHMTECQKYFPK

>HarmOBP7

MFRFGVLSFVVLLFCMESSYALSSEEELSIKEALHPFVVECAEEYGMTEEMFEEAKKKGSAEDIDPCFMSCFLKKTGFFDDSGKFDAEKSISFAKEHITSESAIKFLEAGAGECVKINDEDVSDGENGCDRAKLLFDCLTELKKKMSE

>HassOBP19

MIRSCFVLVAVLQVLGVSAQEGPGGPPGDPRQHPILSKIPRKCWAPPPGIDIYRCCPIPKLYPDEIMEQCGIKRASGDPSEEPEKPQPGPKVPCKEGICLMQNANLLQQNNSVDYTKLRNFLDQWADTNAEFTDAILAAKKICAQDGGPAGPPVCEQDRIFFCLTSNILWNCNLRKLDGCDILQEHMDECRQYYVQDEPEE

**b. CSPs used in fig 5**

>AlepCSP14

MQIVVFLSVVCVGLVAGLHVQAGPQMTDAQLDQTLADKNTMQRHIKCALGEGPCDPVGRRLRTLAPLVLRGACPQCSMQETRQIRRTLAFVQRNYPWEWAKIVRQYG---

>AlepCSP20

MQIKYALVLCCVAATAVAQTQRPAVSDTALEDALQDKRFIQRQLKCALGEAPCDPI-GKRLKTLAPLVLRGACPQCTPQETKQIQRTLSYVQRNFPQQWAKIVRQYAG

>AlepCSP7

MKVLVVLPVLVAFAAAAAAELSPAELSMLEAFDYETLLANKELSQKLFDCMLEKGDCGEYKQVADLSMKTLQSKCAECTPAQKAKYENVLKQLKEKYEPVYNELLKKAGATQKT

>AlepCSP18

MKCIYVLSLLLAFVAVQAEDKYSTENDDLDIDAVVADLDTLKGFLGCFMDTVTCPAVPADFKKDIPEAVKTNCSKCTDAQKHIFHKFLLGLKEKLPSDYEAFKKKFDPENKHFTALEAAVASF

>AlepCSP13

MRNWLLCLCVLTVVVSCYSQGPNRYENFNTDAIIQNDRILLAYYKCVMDKGPCTRD-GKNFKRVLPETLATACGRCNPKQKTIVRTLLLGIRSKSEPRFLELLDKYSPDRSNRDALYTFLVTGN

>AlepCSP16

MIENKRSFRVSLIFTYIFLVTVLAQEKYYDRRYDYYEIDSLIQNRRLLKKYLD

CFLGKGPCTPIGKVFKQILPEAVATACKKCSPSQRRLARKAFNAFDRFFPDTYVEFVHKLDPKNKYYEAFENAITNA

>AlepCSP4

MRAVLLMCVFVYAVVAQDVNDMLNLPKYDSRYDYLDVDAIFTNKRLVRNYVDCLINAVRCTPEGKALKRILPEALRTKCVRCTERQQRTAVKVIKRLKNEYPDEWAKLSSRWDPTGDFTRYFEEFLAKENFNTIPGSGSAIPTSSPLVPPRVTTMPATVATPAPGPTEPAPAQPAIFNRFGDDDEVMMGSPSSAGMTPRPMTQATTRPTTTMRPTTTMRPITSKPLSPRPTMMTWSGAATNTQPTRFPLRPVAELPIPYSTAITLIDQIGYKIIKTTELVTDLLKHTVRAVVGR

>AlepCSP8

MKVIVAIALLCIVAVAWGKPAGTYTDKWDHINVDEVLESQRLLKAYVDCLMDRGRCTPDGKALKETLPDALEHECSKCTEKQKTGSDKVIRHLVNKRPDLWKELSTKYDPDNIYQERYKNKIEAVKQ

>AlepCSP6

MHSTIAMLLLVYLTIQSNATETSTYTTKYDGIDLDEILNNDRLLTGYVNCLLDIGPCTADGKELKQNLPDAIENDCKKCTEKQREGSERVMHYIIDNRPDDWVKLEDKYNTDGSYKLKYLASKLTEADKETNVTTSEENTKNVSKESSKE

>AlepCSP5

MKFIIAVSILFACAQAAQYSSKYDNINLDEVLKNRRLLTGYMKCALDEGPCTAEGKELKYYISDGLQTGCSKCTDRQRRGIKQVMEHLIKYEPEYWKRACDKYDPDRIYTKKYEKEVHSWH

>AlepCSP9

MKFVLLLCVMVAAVLAEDKYTDKYDNLNVDEILTNKRLLEAYVNCVLEKGKCTAE-GKELKEHLQDAIETGCKKCTEAQEKGAQKVIDYLIKNELGYWRELTDKYDPTGTWRKTYEDRAKAAGIKIPE

>AlepCSP10

MNYLVLSVVVTLAAFVAAETYTDRYDHINIDEIIDNRKLLVPYIKCTLDQGRCTPE-GRELKAHIKDAMQTSCSKCTPKQRKAARKVVKHIRAKEQDYWKQIIAKYDPNDEYKENYETFLETTD

>PxylCSP5

MKVVFLVFVLTAVVYSHPHDSHYTDKYDNIDLDEILNNKKILTSYINCCLDLGKCTPDGKELKSHIREALENKCGKCTEAQKNGTRKVMTHLINFEPDYWNQLCAKYDPEGKYKAMYEKEYKTLVH

>AlepCSP12

MKACIALCVLSVAVMALARPEDSQYTDRYDNVNLDEILSNRRLLTPYVKCLLDQGKCAPDAKELKEHIWEALENECGKCSEKQRKGTRRVISHLINNEEDYWNELTAKYDPERKFTAKYEKELKEINA

>AlepCSP1

MKAVFLLCALVVVVSARPEAQYTNKYDNVDLDEIIQNRRLLVPYIKCGLDQGKCTPD-GKELKSHIKEALETYCAKCTDVQRDGTRRVIAHLINHEPEYWRELSAK

YDRDGKFARKYEDELRTVA

>AlepCSP22

MKTVFLLCALVVVVSARPETQYTNKYDNVNLDEILQNRRLLVPYIKCCLDQGKCTPDGKELKSHIKEALETYCAKCTEIQRDGTRRVIAHLINHEPEYWTELSAKYDRDGKFAHKYEEELRTIA

>AlepCSP17

MNSFIVLCLFGLVAVSLARPDSTYTNRYDNVNLNEILSNRRLLVPYIKCILDQGKCTPEGKELKSHIKEALEEDCAKCTPTQRDGTRQVMGHLINHETDYWNQLKAKYDPQSKYASKHEQELRTLKN

>SexiCSP1

MKSFIVLCLFGLAAVAMARPDGSTYTDRYDNINLDEILGNRRLLTPYIKCILEEGKCTPDGKELKSHIREALEQNCAKCTDAQRNGTRRVLGHLINNEEESWNRLKAKYDPQSKYTVKYELELRKLKQ

>AlepCSP3

MNALLVAVFALVASSALAYDEIYDKIDVDKILGDDALFSAYINCMLDKGDCSVEHSADFRKLLPEVIATSCAKCSPIQRQNVRKTVKALSEKRPDEFAEFRTKYDPKGEYEKSFTEFVMGTD

>AlepCSP19

MKIVLVTLCLALGVLAEEQYGSANDDFDISEVLHNERLLQAYGRCLLDKGPCTAEVKTLKEKLPEALETRCAKCTEKQKQMGKALAQEVKKNHPDLWKELVAHYDPEGKYQEAWKEFLKE

>HarmCSP2

MKVVLLTLCFALGVLAQDQYESANDNFDISEVIGNDRLLHAYANCLLNKGPCTPEVKQVKEKLPEALETRCAKCTDKQKQMGKALAQEVKKNHPDIWKQLVAMYDPQGKYQQAWKDFLQE

>AlepCSP2

MKLLIILALVAAAFARPDDAHYDSKYDNFDVDELIQNERLLKAYAHCIIGDGKCTPE-GNEIKGWTPEAVQTSCGKCTEKQKVLVAKCIKAIREKLPEEFELLVKK

NDPEGKHKEGLKSFLEKYA

>AlepCSP15

MRVLIVLSCMLVVAFAAEKYNAKYDNFDVETLISNDRLLKAYINCFLDKGRCTPEGSDFKKTLPEAIETTCAKCTEKQKGNIRKVIKAIQQKHPKEWDALVKKNDPSGKNRVNFDKFIQGSS

>AipsCSP2

MKIILALCVLVAAVSAYDTRYDDFDVETLVGNVRLLKSYGHCFLGTGPCTPEGTDFKKTIPDALQSGCGKCSPKQKHLIRTVVKGFQTKTPDIWQQLVKKEDPHGEYKEIFTRFINGSD

>AlepCSP11

MKTVLVLCLLIAAVYSRPDTYDTRYDNFDVESLVENVRLLKSYAHCFLSTGPCTPEGTAFKKTIPDALQSGCSKCSPRQRHLVRVVVKGFQSKTPDLWQQLVKKEDPNGEYKATFTKFINASD

>AlepCSP21

MKTVLVLCLLVAAVCSRPETYDTRYDNFDVESLVENARLLKSYAHCFLSSGPCTPE-GSAFKKTIPDALQTGCGKCSPKQRHLIRVVVKGFQTKTPDLWQQLVKKEDPNGQYKEIFSKFLRSSD

**c. ORs an IRs used in fig 6**

>AlepORco

VKTQGLVSDLMPNIRLMQAAGHFLFNYHSENAGMSNLLRKVYASTHAILIVINFACMGINMAQYSDEVNELTANTITVLFFTHTIIKLSFFALNSKSFYRTLAVWNQSNSHPLFTESDARYHQIALTKMRRLLYFICGMTCLAVVSWITLTFFGESVRLITNKETNETLTEVAPRLPLKAWYPFNAMSGTMYMIAFGFQVYWLLFSMAIANLMDVMFCSWLIFACEQLQHLKAIMKPLMELSASLDTYRPNTAELFRASSTEKSEKIPEAVDVDIRGIYSTQQDFGMTLRGAGGRLQNFGQQNANPNGLTPKQEMLARSAIKYWVERHKHVVRLVASIGDTYGTALLFHMLVSTITLTLLAYQATKINGINVYAFSTIGYLSYTLGQVFHFCIFGNRLIEESSSVMEAAYSCQWYDGSEEAKTFVQIVCQQCQKAMSISGAKFFTVSLDLFASVLKMRRLLYFICGMTCLAV

>AlepOR3

KKGGVQNSTFEHSLYFAYPFDFMTNVNGYIVTCISHWIISYLCATWFCMFDLILSLMVFNLWGHFKILTNILDNFPRPSSKSNLTWNGSVVQAEKYSKEELKQVFETLRDCIIYHRQILHFTNTMSDVFGPMLFIYYAFHQASGCLLLLECSQMTAAALMRYLPLTLIVTQQLIQLSVIFELVGSESEKIKDAVYGVPWESMDCPNRRTVIIFLMNVQEPVHIKALGLANVGVTTMA

>AlepOR4

KQLKNASRANYRNFLLKEYFDFDSPDIDLYNFHPQFRLFFALTGVSFNNKGTWKRFIWPSFCIALSIVAMVLEKLIIWHGISLKDYAIATECFCYWVILGAIPTVSGGISTNAAKIRDLVKIMNEEFIFVCSLGPKHRKPFLEVQLIIWQLCYAWFTFVCFLSSLYMVLPIAGLTYQSLFATIDENTVRPLQFPLWLPHDDAYRSPYYELLLALEMTMVIMFDQHFAGYIYTLFHILLHYYAIMNMIIIDLSVIFEGLDESVTLLPVNDKRRRETQHILNARIRRIVTWHRSVFNAVKTVSTVFGPPLMYQVSVSSIVMCLIAFQIAVNLENGELDIMFYLLGVATVLQLWIPCHLGTMIRNKAFEVGDAVWTCGWHETRLGLMIRNDMAIIILRAQQPVTIEFTGLPPVQLETFSNCMSTSYTYFNMLXTIQQLRNLESNFKGNFIRSRYYRKCTIDKIEX

>AlepOR38

XEKMVTVCERYEDDALMAEKYKIMKINVIGYIVNVYGCCFFFVFEGIRKRFSGSHFVTVVTYYPKYEDDSMPATTVRILATLILYMMMLTMIYSADTFTMVYLIMYKYKFITLKKYFENLREEFFALIDRGEHDMATEKLADGLVEGIKMHHALIGLSSDIYKAFGTVMALQVCQSSGSAVSLLLQIALSDQLTFIATVKLMSFVLALFFLLGLFLCNAGEITYQAAGVADAIFYCGWQSCX

>MsepOR2

MMTKVKAQGLVSDLMPNIKLMQAAGHFLFNYHSENAGMSNLLRKIYASTHAILIIVHFACMGINMAQYSDEVNELTANTITVLFFTHTIIKLGFFALNSKSFYRTLAVWNQSNSHPLFTESDARYHQIALTKMRRLLYFICGMTCLSVVTWITLTFFGESVRMITSKETNETLTEVVPRLPLKAWYPFNAMSGTMYIVAFAFQVYWLLFSMAIANLMDVMFCSWLIFACEQLQHLKAIMKPLMELSASLDTYRPNTAELFRASSTEKSEKIPDAVDMDIRGIYSTQQDFGMTLRGAGGRLQNFGQQNANPNGLTPKQEMLARSAIKYWVERHKHVVRLVASIGDTYGTALLFHMLVSTITLTLLAYQATKINGINVYAFSTIGYLSYTLGQVFHFCIFGNRLIEESSSVMEAAYSCQWYDGSEEAKTFVQIVCQQCQKAMSISGAKFFTVSLDLFASVLGAVVTYFMVLVQLK

>SlitOR12

MTSLYRKFLFKKKLKQRLDERVYNKSDYDASYAPTKKVLGWVAIRMTHNISEKTTMLWDMFYWFEMMNLFLVGPSELVSMLTTAYEAKTFRDSIKVFRTMPCFGCVVLSMFKSIKMVVHRPVYENLANELREMWPEGEVSEEEHQIISAALKQLNFIVKGYYWCNNALLISFLSPPYFITLARYFGYDSPMGLHFLYWLPFDPYQSVYYEITLVLQTWHALVVIWFNVAWDMLFCLFLCHITTQFDLLARRVQRLFYVQVDNQLVSSYPMASVSKEFIQTEGERVNSYGAQYWEARHQKEITEIVLRHHSLIRLTGDVENMFSLALLINFMNSSIIICFCGFCCVLIEKWNEVAYKSFLVTALSQTWLLCWYGQKLIDSSQRLADALYGCGWYNSSKRARSAVLIMLHRAQKGIYVTTHGFSVISLASYSTIIKTAWSYFTLLLNFFKEKSVN

>SlitOR16

MNLKKFLFENESVVGISSPSEYIYIKIVRFMLVIVGSWPRREIGEPEPRYQTIMLNSLFFCVVNAALFGSISYVYMHTSELSFLEIGHMYIVILMSGIDMSRVFTLTLSQKYRDLAKEFLTKIHLFYFKDHSPYAMLTHKKVHLICHLFSLCLLSQMLTGLSLFNLIPMYTNYSSGRYASGGTQNSTFEHSLYFPYPFNTSTDFNGYVVACILHWLLSYLCSTWFCMFDLFLSIMVFHLWGHFKILINSLNNFPKPSAETHCELERGININAEKYSKEELVEVSQKLKECIDYHREIIQFTNAMSDVFGPMLFCYYVFHQTSGCLLLLECSQMTAQALIRYVPLTIILTQQLIQLSVIFELVGSESEKLKDAVYGVPWECMDASNRKVVAFFLMNVQEPVHIKALGIANVGVTSMAAILKTSMSYFTFLRSM

>CpomOR24

MKAFVSTAKTFLYKNDFEWNKEITLQNFHPQLQIFLAINGVFFNNRESKIRFILPVLSTLITLVAVAFEIFFIWHGISMNDYGFATECFCYFFILGSVGIVYSSVLLNRVKVFKLLHNMNNDFLFICNLKAEYRDTFLTGQLLIWRLCWSWIVFISFVSLLYISNTLLYLLYQSTLATQDEHMIRPLIFPMWLPEDDPYRTPNYEIFLALEVILIFVVLVTFGLYVYILFHLLLHYYNLMDVILIALDDLFDGLDESVVALNRGDPRRQAVQDELNIRMGQIVRWHLSVFDSVDDISSVYGPTLVYQVMFSSIVICLMAYQVAEQLSEGKVDYLFGILGIGACLQLWIPCYIGTLLRNKGFFVGDRCFYCGWHETPLSRLLRPDLIIFIQRTQRPVAIKFTGLPHLQLETFSSIMSNAYSLFNMLRQYK

>BmorOR35

MKLWQSIREFGLEYCDLPTTLQNVASLLRAITLNIDSRHTARIPFICYVMTVVITLSYFYVFLVSMAWFVFVRSAETRDYLAAMVVLSLGISSEIGTLKFFYTFIYIKKVQRIVREYLECDHMVVPESRFADNVLKTMRNVKKRAILYWVVVIGNGVVYVTKPLFMSGRHHMEDRYIVYGLEPMFESPNYEVAYFLMMFGLCFICYPPANVTVFLIVVVGYTEAQMIALGEEMLRIWEDAVAHYNNKYHTVGALTNSSEKNKIINQYVKFRLTEIIKMHTTNIQLLRQVEFVFRSAIAMGYVFLVLGLIAELLGGLENTYLQIPFALIQVLVDCYTGQKVMDASSLFEQAVYDCKWENFDKSNMKTVLLILQNSQKSMRLSVGGITVLGFSCMMSVMKSIYSAYATLRTTMS

>BmorOR42

MDIPKFEELLKQIKMNFWLMGIPFDNPKIQIRYYVLLLPLSLMLIEEIAFFGSRMSSENFLELTQLAPCICIGVLSVLKILALTAKRQKIYELTQNLECLHKIILNDTRKTELVRKNLVLIKFITKYFFVLNAVLIFVYNFSSPVIIAYNYIVSNEVQFVLPYAVLLPFKTDSWIPWLIVYVYSIFCGFTCVLYYATVDVLYCVMTSLVCNNFSLISFKLQKVNRNTAHLLKEVVKEQQYVLKLAEDLENIFTAPNLFNVLIGSVEICALGFNLMIGDLTQIPGCILFLSSVLLQILIMSVFGENLISESSRIAEAAFLCKWYEMDQKSKKTILTIMIRSHKPKKLTAYKFSVISYGSFSKIISTSWSYFTILRTMYTPPGTKFQDDL

>SlitOR19

MSNQMQMYKPNEMTKLLGKLNVICFICGLRNIWVEDVKLTNRFISARFCNRLLLFAEIFYIIFATTILGSLLTQKNLSEKQKTDQMMFSITLPGNIMFHYILLYRRHEIRNLLYHLAVELKEHYNDEDLEQEMIKKIKVFSISLCGLVGMVVVSYGLSAFYRVVTAGETFVTITSAWPDIHDRSTAAGLVRVFVYFWWYPFAARIMVTFLILVTMLVSICYQFKNLQSYFYSLDEIFSDGTQSQEQKEKKYENAFKLGIKMHSLTLWCKNQHQHVSKELFATEIFLFFGMLLSQLTALLAGDRNMTQLCTMFITSVTTCLALGFFMWNGGDITLEASKLSEAMYCSGWQNCYGQSSVRIRKLVANALRQAQNPVVYKTLGIVEFSYESYVRLVKMPYSAVSVFY

>HarmOR18

MEMKVDVLPEKKYKGFNETFKLCAFSLAFAFLYPNRTTALRRCITITLIVTFCGGQLFWFITYTFKCLYTLDIYNFARNMTLAVVLVLFFIKTYYVIYATSKFAPLLDKISDDLLEANNLEEEFQVLYDDHIKIAKVGEISWLLIPTIMSALFPIYAGALMTIESIQTDDYERRMVHDMELLFVEDIRSETPFFQCMFAYNCVQCVVLVPNYCGFDGSFCIATTHLRLKLKLMTLKVNKAFKYSKSRQELRMRLYDSIKDHQDALDFYVQLQNVYGPWLFAVFLLTSFMISFNLYQIYLLQRIDPKYTSFGVVGVLHIYLPCRYASDLTRVSEEIPDDLYLAQWEAWADPSITKLLMFMITRAQQEMIVTGMGLVVFNMEMFKSILQTSYSFFTLITA

>HarmOR20

MDEELEFKPFHETYRLITFSLCIAMIYPNPRTEKWRLFSIPILIATVAPVAIMIFLDMYKCWKNGDIVNIIRHSTVVGPFLGGFFKMILMYHKRVQAKQILDEFDRDHLMFNTVAETYKDIARASIRNCQIYSERLWACLVTTCVMTFPVMAIVLNIYNFMFKSEPTKYMIHDLEKPFSKEPEERFESPYFELLFVYMFYAAILYVVNFTGYDGFFGLCVNHARLKMELYCKALEEAMMADREEVYGRVIAVIREQCRMFRYVDLIQDTFNIWLGIIFIATMIQICTCLYHITEGYGFDIRYMIFVYGAVVHIYLPCRYAAKLKAMSMETSNRFYCSGWERVDDERVRKMIVFMIARAQVPNEITAFNMMAFDMELFLSILQTSYSMFTLLRS

>BmorOR27

MPSSFFLPNLENPDYPSLGPTLKGLKYWGMWQSGGIKRILYNSIHAFATFFVITQYVELWIIRNNVELALRNLSVTMLSTVCVVKAGTFVCWQKYWSGIIGFVSNLEKEQLSKNDAATQAAIVKYIKYSRRVTYFYWSLVTATVFTVILAPLVGFLSSPERELIANGTLPYPEIMSSWVPFDRSRGFGYWVTALVHTLICFYGGGVVANYDSNAVVLMSFFAGQMKLLSINCSRLFDDGNEVISNNEAMKRIKECHYHHVFSTIFNSLMSPVLFLYVIICSLMLCASAVQLTTDGTSNMQRIWISEYLMALIAQLFLYCWHSNQVLYMALEDRLGGLFEACLESGRFPSKWKTGRLVLLRKDGRPADSPAGYRPIVLLDEAGKMLERIVAARIVRHLTETAPDLSAE

>BmorOR20

MIQASKYPNSKTKELFRKIAHIAYICGLPNFWIEELNLPKSFIRVYDKIVRIFNVATYFFLGIEIAAHFTQHHLTNKQKFDLLLYSISHPILNGYGVIVSRQVGNVKKVLLDLIVNLKVKYNDPVIEEAMIKISMTYSVSFITNCVLSMLTYTFDALLMVYKKGVTFNVIITAWPDVEDTTTEASIGRIGFHIFWWLFVTRPFAVYVLVINLTTCLSHQYMNLQSYFFHLEDIFKENLSQNEKEAKYEAEYKIGVMLHANTLRCTRRCHMVWNGVMSGQIIFNISLIVIIMAQMMNSDRTLVNTFGTVLTASAILISTGFFMWNAGDVTVQASRLATAMYCSGWQNCRGKSSVSIRNMVMNTIAVAQRPLVLRGLGVIDLSYQSYLSIVKASYTVFSVIY

>HarmOR11

MHLAGNAVTGITGPMDYKYMKVLRFVLRIISGWPGKALGEKTLRIEGMGHAYYNTILSLVYLALGIAYLKKNFHRFDFLELGQLYIVLLMNMLSTSRAFTLCLSQKYREVAKIFIQKIHLFYFKEKSDFAMKIHITVHKISFISAVYLSVLLFIAACMFNLIPMYNNYSAGRFASFDNLENTTYEQAISCLYPWNFETNFNGYLAATLSGWYGTILCGSSVSMFDLFLCLMIFNLWGHFKILIYNLEHFPRPASEVVDAEGEERSGRTVGSEMYSQSELEEVAVLLRDCIQYHMLIYNFTNNMSDAFGMALFIYYSFHQITGCLLLLECSQMTAAALTRYLPLTIIMFGELVLLSIIFETIGTMSEKLKDAVYKVPWEYMDTKNRRTVLIFLIKVQEPIHVKAGGLVDVGVTTMASILKTSFSYFAFLRTF

>BmorOR13

MAPKQIDCFEINWKFWKFLGIWSENKPHRYYKYYSKIFITFFVILYDVLYTINFYFVPRQLDLIIGEMLFYLTELSVLSKVFTFIIMRHKLKIIFEILESDAFQTDTEEELKILHRAKVFIKRYWKIVALVSITANLTHISSPLLKNLIFKVELVLPVCSYSFLSESFLKTFEYPLYFYQIVGIHFHMLYNLNIDTYFLGLMILIIAQLDILNVKFRNLKSGKDHTQLNESIMGLNKNLDHYNEIERFCSLVQNIFSFTLFVQFSMASCIICVCLFSFTLSVPVEYYIFLATYMFIMIIQIMVPCWFGSRIMDKSILLSSAIYNCDWTSNSKDFKINMRLFVERANKPLSITGGKMFSLSLATFTSIMNSAYSFFTLLRYIQTRE

>BmorOR60

MVRPCRYFAIHFILLRFLGLGWWHHPHENETRNYPGLYLYYSILTQLVWVVGLVGLETIDPFVGEKDMDRFMFSLSFVITHDLTLIKLYIFYFRNVEIQDIVRTIEIDLYRYYQNDDKIRATIRISRIFTAAFLFFGWVTIGNANIYGIVQDLRWKDIVKNLNETTSKPLRTLPQPIFIPWPYQEDKHYILTFILETMGLLWTGHIVMTIDTFIASVILHMSTQFAILREAIVTAYDRTMIALSEGALQSGVLCENSNGNEENNQIFLESFYSKEHIESVLESTLLSCIRQHQLLIGCVEKFSKTYSYGFMTQLLSSMAGICVVMVQVSQGASSFKSVRLVTSLAFFFAMVIQLAIQCFTGNELTIQAERIADAVMESKWEKMPVRLRRLLLVTMMRAQRPLHLTAAGFAYIDNTCFLSILKAAYSYYAVLSQKQG

>BmorOR56

MKLLEKLEDPDRPLLGPNVKALKFWGLLLPESRSKKYFYLFMHFAVTVFTATEYIDVWFVKSDLALLLNNLKITMLATVSVLKVTTFLLWQNAWRDLIGYVSRADLEQRATSDSRKLALINGFTGYCRKITYYYWFLMYTTVAIVTVQPIFKFFSSAAYRLDVQSGNGTYLQVVSSWIPWDKNTLPGYLLASIYQTYAAIYGGGWITSFDTNAIVIMVFFRAELELLRIDCAALFDDEKSFGDMAFMRRLKECHRRHTELVKHSRLFDSCLSPIMLLYMFVCSVMLCVTAYQITIETNPMERFLMTEYLVFGVAQLFMYCWHSNDVLYASQDLSRGPYESAWWSRDVKYRKNLYILVAQFNKVIVFSAGPFTKLTVATFIRILKGAYSYYTLLSQSQMNKT

>BmorOR16

MSFNSEDLYLNRAKFVMKYLGVWVPPENENFARKFYKIFMMSLQHLFLFFQIIYIVEVWGDLEAVSQASYLLFTQACLCFKITVFQINMNKLKELLKQMNGYVFQPKNINQQNIIKVQATRIKRLLFAFMISSQLTCGMWALKPLFDDVGSRKFPFDMWMPVSPERSPHYHLGYSFQLVTICMSAYMYFGVDSVAFSSVIFGCAQIGVIKDKIMSIKPLGIYRNHKTYTKISRYNRKTLIECVKHHQAVISFTELVEDTYNSYLLFQLVGSVGIICMSALRILVVDWRSVQFFSILCYLSVMISQLFVCCWCGHELSATSEELHTILYNCAWYDQDVKFKRDLNFMMARARRPILLRAGYYISLSRQSFVSILRMSYSYFAVLDQTNK

>BmorOR33

MIYYRKCKMELNFDKIFKIAIISQKFSGTYPYTKRDKKWATHFILMHGELTIICMLFIYNIIEFDLKAADYSQMCRNMCLSFVYMVITLLYINMLYYQSKLKMLIETMKAEYELAKTMSEEEQNVILEYAKKGRWLCRAWAILTTCGMAQFFLKSIVCTIYSAIQGNFRIVQYYEVICPEVIERHRNNPVIFITLYFCTFFYSLYTSALYTSVLPLGPIFLLHGCAKLEIVRLNIKNLFDNDDYVVQERLKKTVLQMQDIYCYSHEINECFQILYEFLLKATSLVLPITIFAVIQALGRGQFIPEFFAFIFGAFMVGTTPCYYSNMLMEKSEDVRMTLYSCGWETRFDLNTRKCIILMLCRALRPVSIRTIFRSVSLTTLTDVFQQAYALFNLLNAVWN

>HassOR17

MFLRSECARSVAPHVRVLRRVGFLRGAALSSRGRAERLALRSYHALALAATSLYVLQQAVYAYQERGDMDKLSQVMFLMLCHVTCVVKQIAFHVDADRIDRLIASLDEPLLNQCAGERGALLRGTARGAARLLRTYAGCAVATCVLWIVFPVIDRIQGISFEFPFWTGFSYDHNAVFTFVLLQSFYCTNLVAIGNTSMDAFMATILDQCKTQLRILRINFESLPERARALHVESGENYDTILDKLFVDCLVHYNKITEMCTELHDVFAVPLLVQFGVGGWILCMAAYKIVSLDVLSIEFASITLFITCILIELFIFCYYGNEVTVESERVSQSLYSMEWRRARLTFRRSLVLVMERAKRPLRPAAGRVIPLSLDTFVKILKSSYSFYAVLRQTK

>HarmOR9

MLDQFDRCLKSVNLYLKFLGLYLESKDTDKTFVERTRSHRLYFAHLFSLNLEVVAQVLWVLEAVITGKSFVEITRLIPCLILCLISNFKTLSLLYYGRHNNEFIVTMRSLLLNQMQVEEKEHRFRKNLIDKHVLILTSISKKISYVIVLDLLMFALAHAFIIIPHYFKTDEVKLEMPFIAYYPFNEFDLRVYPWVYFHQVYSAVIAMIMVYGPDCFFFTCCTFIHIQFSLLNNDMERIVTEETPRYDKTKFKKLAVRHIELMRCVNLLEKIFSKSILFNALTSSVIICVTGFNVLVVDNIVMMASFTAFLIFGLMQIFLYCYYGDTIMRSSMEVSTSIYNSLWYNIPAADRKGFLIVIIRAQKPCALTADGFFKMNLSAFASILSKSWSYFALLKTMYHPE

>MsexOR50

MISIWTGIRKFGLEYCDLPTMLWNVAFLLRPLTLNIDSRHKKPIPLLFYVLTVVITSSYFYVYLISMSWFVFVRSLETKELIPAMVVLSLGISSEIGTLKFFYTFVYIDKVRKIVDEYLECDALVVPFSRFSKNLLTTLRFVKKRAIIYWLVIIGNGFAYWSKPLFMKGRHHLEDNLVIYGLEPMLESPKYEIAYFLMTAGVCFICYPPANVTILLIVVVGYTEAQMLALSEEILNIWDDANDHYNNLPSTNQNTNELKSKIINKYIKDRLKDITKSHARNINLLRQVEFVFRGAIAIGYVFLILGLIAELLGGLENTFLQIPFAFIQVAIDCFTGQRVMDANIVFETAVYDCKWENFDKTNMKTVLLLLQNSQKTLTLSAGGXTMLNFKCLMSVIKSIYSAYTTLRT

>BmorOR38

MNLSQSVNEQANEYVKMRLERISKIHSPMLPFEDIQDFRELCCIPLAVYAVTGSITASYVYAFLISLLWFLFARCTDPEDFQVAMVVFSLGISSEIGSTKFFNSIIYIKELRKLFKDYLLYDATCPAQGRLRLHLLTTLRYVKRRAIIYWLVIIGNGFIFAIKPLLVEGRHLAQDDLVLIGLEPMRQSPNYEIAYAIMTMGVCFICYPPAHVTMFLIIIVGYTEAQMLALSEELKHLWNDAIEHYEKHSRTEREADAAMKSKILNSFVNFRLVQIIKSHSTNVNLIGRVENVFRGSLAVGYVFLIVGLIAELLGGLENTYLQVPFALIQVAIDCFIGQRVNDANIDFEKAVYDCKWENFDKRNMKIVLLLLQNAQKTVSLSAGGIAKLNFSCFMSVIKSIYSAYTTLRTTMK

>HarmOR10

MAVKNTSLFLGRPKKILSAHGVWPHPNNFVILRKLYMLFVMWTQYSFLLFEIIYIADVWGDIDAVSEASYLLFTQASLCYKSTAFMVNKQSLLELLEIMDCEIFEPKSAEHEKILAAQARKIKRLCLFFLTSATTTCTLWAMIPLFDAASKRSFPFRIWMPVTPLKSPDYELGYLYQMVSIYISAFLFISVDSVAVSMIMFGCAQLEIIMDKIQKIKYVFESADSEEGRRNIIKTNNEFLVECVKQHQTVERFIQLCEDTYHANIFFQLTGTVAIICNIGLRISIVEPNSVQFFSMLNYMVTMLSQLFLYCWCGHELTIRSENLREWLYQCPWYEQDTEFKRALFIAMERMKKPIIFKAGHYISLSRPTFVAILRCSYSYFAVLNRVNTE

>CpomOR28

MVSVQKIISLAKRLEDPKHPLLGPNLKGLYVYGLWQSGSKFRNTCYNVIHFCAFLFVISQLIELWIIRHDYLEALHNLSLTALGMVCIFKAVSYVMWQSDWKKLVEGISAEEISQSDSLNDACIELKQKYTNYVRIVTYLYWNVVVSTNITMVSAPFLKYATSSEYREQISNGTEPLPQIFSSWFPFDKTTMPGYSLAIFIHILINIHGGGVIALYDSNAVAVMVFIRGQLGMLREKCKHIFDEYELVNQEIILGRIKECHRHHNFIMRHSSLFNSLLSPVMFLYVLVCSGMICCSVIQFTSEEATAAQKVWVLQYTTALVSQLFLYCWHSNEVVVECQHVDGGVYDSEWWKGDTHVRKQLAMLGGKLTHNIVFSAGPFTTLCVPTFIDVIKGSYSFFTLLTQMQE

>BmorOR36

MVFNSKKNIISLFSLLEDSRHPSVGPHLRLLSLTGIWYPNSKTNITLLKRACFYVIVLFFVSQYLKCIIKFKIDSLQLILEYAPFHMGIVKTCFFQKDYNVWQDLVSFISKTERDQIAKKDPKSIKTIQSYISRNRKITYSFWALAFIANIGVFSKPYQNNQSDVNGTVTYNHLFDGYTPFSEEPPGYYFSMGIETILGHVVSFYVLGWDTLVVSIMIFFAGQMQMSRLQCSRMINGSPERTHKNIIKCHKFHTDLIKYQKQFNSLISPVMFVYLFVSSINLSVCIVQIAEIEDDFATVLSSFIFLLACLIQLLLFYWHSNEVTVQSELVSYSTFESNWTSTQNKLQKEVALLGLTTSKTLVFTAGSFNHMTLATFISIIRASYSFYALLNSTKY

>MsepOR1

MAYTPRSLRDFLFDYEPPEGVSSPADYAYLIMMRHLLSVISSWPLKILDPLDTGAIQRRKIWVSIQRFFHMAVCLSTVVGGVMYVMLHKKSMTFFELGHLYISLLMTFVIFSRISTLCFSDEYVVVARNFLEKFHLFFYKDRSEYSMQTHKQVHRIAHLFTIYLINQMLAGLFLFNVTPMYNNYSAGNYASGGLKGNATYEHALYFSYPFNASGDLKWYILANIFHWIISYLCATWFCMHDCFLSLMVFHIWGHFKILLYNLENFPRPANRISFIPDNSNTTLTYEMYTQNEQKNVGVKLGELINYHRDIISFTDKMSEVFGPMLFAYYGFHQASGCLLLLECSQMTAAALTRYLPLTIILFQQLIQLSIIFELIGSISDRLKFAVYGLPWESMDKKNRRTVAFFLMNVQEPVHVKALGLADVGVTSMTAILKTSFSYFTFLKSM

>HarmOR7

MKIKMSKPLIFDQSIEKLGVLFRFSGMNIKNKIVTPLDTIKYRWLYTLNFLVVFSAIIGSVYYVILGIKQGKNFIEVTSVAPCLTFSILSMIKSLYHLMYEEHIQELIDLLTEHEIRENNREKCIEKEEIIANETGFLNKVINVLYVLNCSMIVVFDMTPIVMIAVKYYKTNEFEMLLPYLDVFSFIPYELKYWPFAYIHQIWSECVVLLDMAAADYLFFTCCTYIRVQFKLLQYDFERMIPDRSISKGLFFEENELRNKFTELLKWHQDIIYSSTILEIIYSKSTLFNFLSSSLVICLTGFNVTIVDDIVIIITFLTFLSMALMQVFFLCFFADLMMTASLEITNSVYNCKWYSANIKVGKQILFVQTRAQEPCKLTAAGFADVNLNAFMRVLSSAWSYFALLRTVYGAK

>SlitOR51

MEDNVAYSTFQGFRPHFDALARVGYFKIVLKPISSLKRFFHNVYRFISWSLILTYNLQHVIRVVKVRHSTNLIVDTLFILLTTLNTLGKQTAFNLRSRRIDDIISIINGPIFAASKPYHVEVLKQNALVMSRLLTLYHGAIFTCGTLWTVFPVVNRALGKEVQFTGYFPFETRSTLAFSLALAYMTVLITFQAYGNVTMDCTIVAFYAQAKIQLQMLRYNLEQLVVYSNTKKFDSIKNQYRDEGEEKTELQERLKKCVRHYNQIVRFAKEVESIFGEAMVVQFFVMAWVICMTVYKIVGLSICSAEFVSMAVYLGCMLAQLFIYCYFGTQLKVESELVNQSIYCCDWLCLSPQFRRQLLVMMQCCGRPLAPRTAYVIPMSLDTYIHVLRSSYTLFTFLNR

>HarmOR3

MTLSVLDRFYLIDDGFFSFNLKYLFFVGLWPEKTLTRNQKILYKMYEHFISFLTTTFIVLAGIGTYQHKDDLVVVFCNIDRCLVVYNFFFKTIIFFIKRNQLRDLIDEIEMSGDEVTEERKKLMANYVMFITGVTAAVIGAFSLLALFEGTMSIEAWLPSDPMESLMNQILSLEILAFCVFPGLCRAFAMQGLVCSMIMYLCDQLIHLQKELRDLTYVKETEMVMRTKFKNAIRKHIRLMGYSGRMENIFKEYFLVQNLAVTVELCLNAVMMTVVGVQQITLLITFLAYLMLALVNAYIYCYLGNELIIQSQGIALAAYESTWTSWPVDLQKDLLIVILAAQRPLKLSAGGMALLCIQTFSQALYNGYSIFAVLNDAVN

>BmorORR

MVVLSLGISSEIGTLKFFYTFIYIKKVQRIVREYLECDHMVVPGSRFADNVLKTMRNVKKRAILYWVVVIGNGVVYVTKPLFMSGRHHMEDRYIVYGLEPMFESPNYEVAYFLMMFGLCFICYPPANVTVFLIVVVGYTEAQMIALGEEMLRIWEDAVAHYNNKYHTVGALTNSSEKNKIINQYVKFRLTEIIKMHTTNIQLLRQVEFVFRSAIAMGYVFLVLGLIAELLGGLENTYLQIPFALIQVLVDCYTGQKVMDASSLFEQAVYDCKWENFDKSNMKTVLLILQNSQKSMRLSVGGITVLGFSCMMSVMKSIYSAYATLRTTMS

>HarmOR12

MEDEPLLIDKTVKNIEFLFRCTGINIKSGTKTRKDMIKSRTVYIINFLWLNIDLAGAVMWFFTGIANSKSFTELTYVAPCITLSFLGNLKSLFLILREKHVDKLIQVLRDLEINEKARPKSEETDAIIKYEHNFVTTVISVLNVLYFVLLVAFALSPVSLVALKYFTTNELELLLPFLIVYPFDPYDIRYWPWVYLRQIWSEVVVIIDICTADYLFYTFCTYIRMQFRLLKHYIERVIPEDDGGGRLTNIEQVRAEFVLLIKWHQDLISSANMLETVYTRSTLFNFVSSSVLICLTGFNVMAISDVAFVATFLSFLFMSLLQIFFLCFFGDLLMTSSTEISEAVYNCRWYLADTSLGKDLLLVQTRAQTPCKLTASDFSEVNLKAFMKILSTAWSYFALLQTLYGAPT

>BmorOR63

MKLWIRNANFTISLSLTLLRCLGFWSPDGLAGNKRLLYNCYSFVFFMFLLGIYILIQVVDMIKIWGDLPLMTGTAFLLFTNFAHATKVINIVIRKNRIQRVIQQANAVLMGVQSEEARRIVKSCDFETSIQLCLYFLLTFVTTVGWATSAEKHQLPLRAWYPYDTSKSPAYELTYIHQVAALLIAAYINVAKDSLVSSLIAQCRCRLRLVGLALASLGQDLKIDYQSQLSPAQENILNLRLKTCVLEHQTVLAAVTELQACFSKPTFAQFTVSLIIICVTAFQLVSQTGNLVRLLSMGTYLMNMIFQVFIYCYQGNKLSVESSEIAGSVYFSPWYLGSVKLRRALLIVMVRSRRVAKLTAGGFTTLSLASFMAIIKASYSLFTLLQQVKQKK

>SlitOR83b

MMTKVKAQGLVSDLMPNIKLMQAAGHFLFNYHAENGGMTGLLRKIYASTHAILITIHFACLGINMAQYSDEVNELTANTITVLFFTHTIIKLGFFALNSKSFYRTLAVWNQSNSHPLFTESDARYHQIALTKMRRLLYFICGMTVLSVVSWVTLTFFGESVRLITSKETNETLTEVAPRLPLKAWYPFNAMSGTTYIIAFAFQVYWLLFSMAIANLMDVMFCSWLIFACEQLQHLKAIMKPLMELSASLDTYRPNTAELFRASSTEKSEKIPDTVDMDIRGIYSTQQDFGMTLRGAGGRLQTFGQQNNNPNGLTPKQEMLARSAIKYWVERHKHVVRLVASIGDTYGTALLFHMLVSTITLTLLAYQATKINGINVYAFSTIGYLSYTLGQVFHFCIFGNRLIEESSSVMEAAYSCQWYDGSEEAKTFVQIVCQQCQKAMSISGAKFFTVSLDLFASVLGAVVTYFMVLVQLK

>CpomOR31

MSKDNMKDNWATTAMRNRSHYRDKNWRTTASSCCMYSTHDCGAYADACKSTMAVATTYKYMRYDSTDRVDDYAKGCRVYKYSKRGVKVTYWVSACSTSAVKAVMGKSYAGVMTYWNDYKNVHVVVMGTDYATSMYVGDVMHCGDNRSKSNTDSATRNRRDYKKNNTVYMKTTTTATSRNGNGTGVHYCYYSNMTGNRAYSCGWKHWDKHVMRTMTRAKVSTVCACDTAMSRAYSNMNAAWA

>BmorOR22

MNKNMNKNHYILKTYCDKIFLVGSGNFWYQKTESRNDKTLLYKIYSCVLFFTYGFMTVLEIMAATMGDFPDDEKRDSVTFASSHTLIMIKFISIIKNKELLKTLNRKMMMICEAHEEQTLMDEMYRIVKINVVAYCVAVYGSVTFFVFEGLRKFYDGSHFVTIVTYYPSKDDDTMLASIFRIATTLVLLVMMLSMIISVDTYTMAYLIMYKYKFITLRHYFKRLRENVDELVAAGKARLAAEKLAQGLVEGIKMHNELLSLSKDIHKAFGTVMALQLCQSSGSAVSLLLQIAVTMYLLLALFLCNAGEITYQ

>BmorOR34

MIYYRKSKMELNFDKIFRIAIISQKFSGTYPYTKRDKKWATHFILMHGELTIICMLFIYNIIEFDLKAADYSQMCRNMCLSFVYLVITLLYINMLYYQSKLKMLIETMKAEYEIAKTMSEEEQNVILEYAKKGRWLCRAWAILTTCGMAQFFLKSIIVCTIYSAIQGNFRIVQYYEVIYPEVIERHRNNPVIFITMYFCTFFYSLYTSALYTSVLPLGPIFLLHGCAKLEIVRLNIKNLFDNDDYVVQERLKKTVLQMQEIYCYSNEINECFQVIYEFLLKSSSLVLPITIFAVIQALGRGQFIPEFFAFIFGAFVVGTTPCYYSNMLMEKSEDVCMTLYSCGWETRFDLNTRKCIILMLCRALRPVSIRTIFRSVSLTTLTGVFQQAYALFNLLNAVWN

>AlepIR1.2

XIASEDYDYMRYVFFDIKTTWDELEYLKKKKKTSNFYHKLSRGVELIREGGTAFHGEYNQLYPHFKTFSDDQICKLQHVDTIPEIMTWISATKDSQWTNVMRVAGAWLTEIGLAKRLISRLRIPQPPCRASLLAERVKLGDIAPLLVLTAFGGVLSVVLLGVEIMYAKAKRNREDVGVSKRALEVDEVESVDESGAETMLAQAVVEFFKHKVVSSIIVLACWPPSDQIKFTRQISQYGMTATFSCDAAILDHIRHHYLQGVLYMAHEHDNITILEQVQNIHFLTKYKWLILGDHVPAVLRKSRYDADIAFLKWEHVASNNTRDSTAISADTSTSIHIYDAYVHPTDGISINLWAHWTSATGLVLTHERERILRRLDLKKHPLRIATPIGHYSSDRYDGTFVDFLEDESMSEEEPGVRSGYGAAMLITEKLNAQDVLIENKLWAAEISNDSMYVMVSTGEADISGAILRILYDRTYTLDYVMAIWPFRVGFTYLAERESSSNMYLEPFSPGVWWSCLAMMGILALVEWITAKSPKEKDGALYTVLTTWLQQDASAVPEGVSGRWTFTVVSVSAMLVHAYYTSAIVSALMSTGRGGPDSLKALGDSKYAIASEDYDYMRYLFFDVKTTWDELEYMKKKKMSPNFYQELKRGVELIQQGSTAFHAEYNQIYPHFKTFSDDQICKLQHVDTIPETLTWISSTKDGQWTEVMRIAGSWLLETGLGKRLVARLRIPQPPCRASLLAERVKLGDIAPLLVLTAFGGVLSVVLLGVEIMYAKAKRNREDVGVSKRALEVDEVESVD

>AlepIR7d.3

TVIWKKNESEYVNEYLKNYPGSVVLSPWSTYNDSKIRAINETVGFKQTIYFATTLNDYEIIMQLINEVIRFPIRFILVLENPVQTNAEISSFIEVTAKNDQADLILISANEAGKVSMSTFFPYSDGSCGNYTPIFLKYGDNMFPKKFSNFYQCPIRTALLEYFPYVTVEIEDGKITSVGGYDGKILMIILHQLNASLEVTSSYNNSVFGTYVNGTATGSIGDLATEKADILIPADILTEKRYTVTLPSHTYHTVDIRWVGRRQREVSDWLKCIVPDKTNFTYLHGLVYISFLIVAMLVRKCKPQMTSVTNRILFQSFIILLGQSAKFVTKSWLLNFLFVLWIWFCFFFRIDYQADLVDALQTIDLEPPFKSIEDAVTKVDGFGGVEVVIDYYRDTPLEHKYQVIPMNELRTYIWRIFKGENFLLATDVALVRSLESYVQILEKRISSTGASFYMRPGWPAAKDVDDVIFSLVEAGFIENLLSDHNNQKWVINRNHDDDVISPASLSMTKLSTLFYALGFMWIVCSIVFVIEIKHHKNXDEEIVDGIELFHYDCSA

>AlepIR76b

XDNMAVSWFCVVLLTYYVVFGEEILVEFYPSQSFLDTQYKINTKREIKSNVKPVTEYNNGTKEINWRQFNQEEIDDGEIYKRALDPVFHGHPKTREEKWNEHFINKTTTFDQIPSLVSLLHNISATYLTDCTPVILYDSQVKSKESYLVQNLFKEFPMSFIHGYIDDNGELSQPNLLHSNSDCQNYILFLSDIRLSAKILGKQPKNKIIIIARSSQWAVQEFLASVNSRMFVNLLVIGQSFKEGDDATLESPYILYTHKLYTDGLGASQPVVLNSWTHGKFSRNLDLFPVKMTGGYAGHRFVVAAANQPPYVFRRIKSDLDGGNPRVVWEGVELRIIKLLAKRNNFSIEIIEPQEPDLGPADSVIKEITMGRADIGIAGMYLTSDRVKKIDLSLPHSQDCAIFITLMSTALPRYRAILGPFHWHVWVALTFTYLFGMFPLAFSDKHTLRHLLNNSGEIENMFWYVFGTFTNCFTFLGKNSWSKTDKITTRLLIGEFSVIEMFISFRLVIDFFSNIFFFRLVLDFHNYNHKLLSGSIIAFVTLPVFPETVDTIKQLLSGFYRVGTLDRGGWEKWFLNSSDPETAKLLKKLELVPNVQAGIRNTTKAFFWPYAFLGSKAELEYIVHANFTATKSKRAVLHISNECFVPFGITIGFPNDSVYSEKMSFDISRMIQSGLIDKITNEVRFEMQRSLTGKFLAVTQGSIKMVSAQEKGLTLEDTQGMFLLLAAGFIMAGTALVSEWMGGFTRKCRITRKVDTPVSVNSRDHLIPSPKTNTESEIKIVEDTESRLNFDSRPSTATSRETLNGLVINVTEDSIDVHNSFNVGRFESRRSSSLDLDREVREIFEKDQQRRRFTAHDMESLDENGGTVSRAAFGDPIKHDKXE

>CpomIR60a

MLKIICLLSIGVNAKVNPHGPTVVSDFSSCVSEIIDKNFAQSGLLFFVDTFNVSTPVAGIRNGIIKSVHTKLKYSVKIAIPTKKDKGICVNNDKTAIEISVKSRMDHFEATSLADYFILIIEDYKDFSYIASRLIRAISWNPRALFILVYFSISNSDDQNIRHAEDMLFCLFKVNVINAVVIIPEVNNVRRANIYSWRPYAPPKYCGHYNESIRNRLIVENVCERGKIKYAKKIFESKIPSDMMGCSLKVLALERQPFISHNPLDPNIESLLINQVAKRYNLSLRYEILNSFRGEKLFDGDWTGALKELTYKKGHLLLGGIFPDDEVHEDFECSSNYLADSYTWVVPRALPKPAWLALFVIFQKTVWLTVITCFVFIALSWMVLAKLSKDPTYRTNLDHYFINTWLSNLGFCAFSRPITNSLRLFFVFINIYCILLLTAYQTKLIDVLTNPSFEYQISTVEELVESGLKFGGSEELHDIFENSTDSIDNYFLDGWIDIADIRDALRDVAIHRNFSLMCSRLELAYVSAIIPELSDQFGKYMYYAFPTNVFTVPLEAVSMKGFPFMKGFSRTLTHFEQHGVNNGVIVYFGGYLLRQRALLLNKFKIEHNSRDPLSIQTLQGGYLALMFGSVCGTFVFIVEIILNTKFVKKLKIL

>DmelIR41a

MFIDLSWSLVLSAIVGKYLNESTICIFWNDKFEFQLLHKSDYISFVGINIKSFDDNGGHYIIDTGLKKKELQNKHLFLDELVIKIIISIEVTHCETFVVFDKDIDRFVNAFNKASVYSIWRSLHNKFVFAHIANESPESRNHFFEDQPNILFVVRDHSSASSFDIKTNKFVGRKAENPSQMILVDRYLASEQRFQFGKSLFADKLNNLQGREVIIAGFDYPPYTVIKHNMSTNAQDMGVSGESDFKNVYIDGTETRIVLNFCEQFNCTIQIDSSAANDWGKVYPNMSGDGALGMLINRKADICIGAMYSWYEDYTYLDLSMYLVRSGITCLVPAPLRLTSWYLPLEPFKETLWAAILLCLCAEATGLVLAYKSEQALYVLPGYREGWWTCTSFGVCTTFKLFISQSGNSKAYSLTVRVLLFACFLNDLIITSIYGGGLASILTIPSMDEAADTVTRLRFHRLQWAANSEAWVSAIRASDEALVKDILYNFHIYSDDELLRLAQDQHMRIGFTVERLPFGHFAIGNYLGPQAIDQLVIMKDDIYFQYTVAFVPRLWPLLDKLNTLIYSWHSSGFDKYWEYRVVADNLNLKIQQQVQETMTGTKDIGPVPLGMSNFAGFIIVWILGSAIATLTFLLELSLTYILKQSNLK

>CpomIR1

AGGVGMRMTMWRLLFLVASAASLPTDWPHMAVDYFQHKHVKYVAHLSCKDAAEIKGVLRLLMNEGIRAAVGLIDQGPMNIMPLLYQYEASVGVLVDGDCINTRDILNNASESMMFDDTHFWLVMNDNCSMGFVEDTFLDLKLSVDADVVVASYCGDIYQLTDVFNFGRVQGNVLETRELGAWTSERGLEIVLQGFKYYNRWDFHNLTLRAVSVIRNSSKEFHEGMLYEPGFTVGVAAMTKISSQLLNLLKEMHNFRFNYTIVGRWIGTPERNSTKAMSNMLLWRDQDISSTCTRLFSNWLDWMDPFFPSVTELE

>DmelIR7c

MLHSAVHNVSLVYALVWAIDNYYGMATSTPLAVVQFPTSRESRRLHNDLIDAALGRSSGTGRIQFLLEDDRVEMTETDTDPPPPSGLTGRPIAIWFLDSLRSYFRLEMYLNQLGSPYKRNGFFLVIYTGLEDQPMESLKIMFRRLLNMYVLNVNVFLQRDGTVHLYTYYPYGPHHCQSSLPVYYTAFQDLAAPANGFGLTKPLFPRKLTNMHGCEMVVATFEHRPYVIIEDDPKTPGGRSIHGIEGLIFRSLAERMNFTIKLVEQKDKNRGEILPDGNFTGILKMMVDGEVNLTFVCFMYSKARSDLMLPSTSYTSFPIVLVVPSGGSISPMGRLTRPFRYIIWSCILVSLIFGFVLICLLKITALPGLRNLVLGRRNRLPFMGMWASLLGGLALYNPQRNFARYILVMWLLQTLILRAAYTGQLYLLLQDVEMRSPIKSLSEVLAKDYEFRILPALRTIFKDSMPTTNFHAVLSLEESLYRLRDEDDPGITVALLQPTVNQFDFRSGPNKRHLTVLPDPLMTAPLTFYMRPHSYFKRRIDRLIMAMMSSGIVARYRKMYMDRIKRVSKRRNLEPKPLSIWRLSGIFVCCAGLYLVALIVFILEILTTNHRRLRRAFNVINRYAA

>CpomIR21a

TCTCYKRVYPFYCFLSDVTKSAKILGKQAESKVVIIARSSQWAVQEFLAGPQSRMFINLIVIGQSFKDGDDDTLEAPYILYTHKLYTDGLGASQPVVLTSWSHGKFSRQVNLFPRKMTEGYAGHRFVVAAANQPPYIFRTIKTDADGGNPRVVWDGIEVRLLTLLSQMNNFSIEIKEPREPHLGSGESVLKEITGGRADIGVAGIYLTSDRIRDTDMSFSHSTDCAVFVTLMSTALPRYRAILGPFHWTVWLALTLTYLFGIFPLAFSDKHTLKHLLHNSGEIENMFWYVFGTFTNCFTFVGKNSWSKTTKITTRLLIGWYWLFTIIITSCYTGSIIAFVTLPVFPETIDSIQQLLDGFYRVGTLDRGGWEKWFLNSSDPKTNKLLKKLQLVGDVPSGIRNTTKTFFLLPFAFLGSRAELEYIIQSNFTKTKKSKKAQLHISNECFVPFGVSLTFPNNSLYSSKLSGDIARILQSGLMDKIENEVKWEMQRTPSGKFLSAGSGTLKLGAITEKGLTLADTQGMFLLLAAGFVLAAAALISEWMGGCSRKCRPQKKEDEPSSAHSREHLIPTPKSDVDSEIKVISDSAESRFRLNPRPDSEDSRDSLEGTIINVTKESIIIHNNYHSSNWDSRRSSSVDIDKEVQEIFEKDEKRRRINSGTVPLKDNQREATASKGAFGDHLSDH

>CpomIR75q2

MVADVIRAMQRPSAVIAMLCWSSNLKLQLYSALEGENVTQITMMQFLKAGTVPERHAQDQHIVFLADLDCPDIISYFQTSSLNKHFRSPFRWILIDSGNNDTSQSYIPNAVGNFDILVDSEVILAHHLGDGSYRLHLIYRIGNNTDWKKEFYGTWDERRRLQKQVMEGEIILRRIDLESYELPICYVLTDNDSINHLYDNVNDHIDTITKVNFPTTNHLLDFLNASRKYVFANTWGYRVNGTWNGMTGYLVREEVEIGGSPMFFTSERISIVDYISSPTPTRSKFVFRQPKLSYENNLFLLSFRASVWYSSIALLLLLVIVLFIVTIWEWKKTRGHEDKKLEADSGILRASVVDVVLLIFGAACQQGSTVELKGSLGRIVMLILFLALMFLYTSYSANIVALLQSSSSQIKTLEDLLHSRIKFGVHDTVFNKYYFSTATEPVRKAIYETKVAPSGSKPRFMPMDEGVKKMQKGLFAFHMETGVGYKFVGKYFQESEKCGLKEIQYLQVIDPWLAVRKNTPYKEMFKLGTKRIQEHGLQSRENRLLYEKRPKCSGQGGSFVSVSMVDCYPALLVLFYGAVFSVGLLFIEILTKRRNDILRKISRAKTLGVDVEDY

>CpomIR75p

SWNGIIERLIKKEGDIGTLTIFTQERLKIIDYIAMVGTTAVRFVFREPPLAYVSNIFALPFTGAVWLAVFICVLACALFLYITSKWEVTMGIHPMQLDGSWADVLILIIGAVLQQGCTLEPRRAAGRIVTLLLFIALTILYAAYSANIVVLLRAPSSSVRSLQDILNSPIKLGASDFSYNRYFFKKLNEPLRKEIYNKKIAPKGKKANFYTMKEGIEKIRKGLFAFHMELNPGYRLIQETYQEDEKCDLVEIDYINEIDPWVPGQKRSPYKDLFKINFIKIRESGIQNCIHQRLHVGKPRCLGAVNTFSSVGIMDMYSAMLATLYGMFMAPAVLLLEIAYKRLMVAREKRMQHNNSHLVY

>DmelIR68a

MRCLWILIVAFISLAMATSIPIPIANPAPLSGYEMQLKILLQKILWVANVKRCFAVITDDLHYPIYDRIFFESVGRRVIPFFVMRTNESDDLQRPSRQVELFVKAIKSSDCELNVITILNGWQVQRFLGYIYDNRSLNMQKKFVLLHDLRLFESDMIHLWSVFIDAIFLKRQLDNKYTISTIAFPGILSGVLVMKNIANWELGKGLNGRILFADKTSNLFGTSLPVAISEHVPMVLWANATKSFQGVEVEIMNALGKALNFKPVYYKPNQTENMDWTELDGGASVAYGSGNPDGYAQNGTHIDSMLVDEVAAHSARFAIGDLHLFQVYLKLVELSAPHNFECLTFLTPESSTDNSWQTFILPFSAGMWVGVLLSLFVVGTVFYAISFLNAIINGNVSSEFFRCLRPNRNVPMDPKIYRRISFRIAISRYRSSKGDRMPRDLFDGYTNCILLTYSMLLYVALPRMPRNWPLRVLTGWYWIYCILLVATYRASFTAILANPAARVTIDTLEDLLRSHIPPSTGATENRQFFLEANDEVARKVGEKMEVFGYSDDLTSRIAKGQCAYYDNEFYLRYLRVADESGSALHIMKECVLYMPVVLAMEKNSALKPRVDASIQHLAEGGLIAKWLKDAIEHLPAEALAQQEALMNIQKFWSSFVALLIGYVISMLTLLAERWHFKHIVMKHPMYDVYNPSLYYNFKRIYPQH

>CpomIR93a

KGFIAVFSIASCEDTWSLFSRAEEEELLLFALTEVDCPRLPPDSAITITYTDPGQELPQLLLDLRTTRAFNWKSAVILHDDTLNRDMVSRVVQSLTSQIDDEDVPTISVTVFKMRHEINEYLRRKEMHRVLSKLPVKHIGENFIAIVTSDVMSTMAETARDLFMSNTQAQWLYVISDTSIRNSNLSSFVNALYEGENIAYIYNITDDREDCKNGLMCYSEEMMNAFISALDSAVQEEFDVAAQVSDEEWEAIRPTKIQRRDTLLKHMQQHIAVNSVCGNCSTWQAMAANTWGSTYGGNVQADNVAAPDNETNEAIQKIELLQVAYWRPSDGLRFTDFLFPHIVHGFRGKVLPIITYNNPPWTILKANESGSISSYSGLIFDIVDQLAKNKNFTTEAHISR

>SplitIR75p

MTGISIVLFFLVTQCSVLIQSKDMENINFIKLFILNDQKPTHLIYGGLCWKKELINKLVVEMSNIGVRTSASFKPRSKYQDHAIMYLTDLDCAQSRTVLSYASSKELFQFTYRWLILVTSPQLQQSKISLLENGPVLVDSDVVLAERVGNMFKMTELHRPGPNGSMISTPRGYYNGSVVDVRAHRELYRRRRNMRGHAITMSNVIQDSNTTRLHLPREDRLKLQYDSITKACWSAAKIGFEMINATPRYIFSYRYGYKVNGQWSGMIADLYANKADMGTNCVIFRDRFDVVTYTDLVAPMRMLFIFRQPPLAYVANVFYLPFSTRVWVTIAVCTAIATVTLFLASKVEIVITKTTTQQQLDGGICDVLLLTMSAVTQQGCYIEPRRAPGRMMVFVLFTALMALYAAYSANIVVLLQAPSDSIRSLPQLANAKITLAANDVDYNHFVFNQSREPLYISIRDRVFPENGKAKLYSLADGVERIRQGLFALHSVAEPVYRQIEATFLESEKCDIATVDYLVTFDSFTPVRKGSPYLELIRVVHKQIRESGIQSAIRRRYLVSKPHCTTKMSSFSSVGLMDMRPVLILMLYGVAVSVIIVFGEIIVHKLINRYYKQKSKVQMVKTIHY

**d. SNMPs used in fig 7**

>HzeaSNMP1

MQLPKELKYAAIAGGVALFGLIFGWVLFPTILKSQLKKEMALSKKTDVRKMWEKIPFALDFKVYIFNFTNAEEVQKGATPILKEIGPYHFDEWKEKVEVEDHEEDDTITYKKRDVFYFNPEMSAPGLTGEEIVVIPHIFMLGMALTVARDKPAMLNMVGKAMNGIFDDPPDIFLRVKALDILFRGMIINCARTEFAPKATCTALKKEGVSGLVLEPNNQFRFSIFGTRNNTIDPHVITVKRGITNVMDVGQVVAVDGKTEQTIWRDTCNEFQGTDGTVFPPFVPETERIESFSTDLCRTFKPWYQKKTSYRGIKTNRYIANIGDFANDPELNCYCSKPDTCPPKGLMDLAPCMKAPMYASMPHFLDSDPQLLSKVKGLNPDVTQHGIEIDYEPITGTPMVAKQRIQFNIQLLKTDKLDLFKDLSGDIVPLFWIDEGLALNKTFVNMLKHQLFIPKRVVGVLRWWMVSFGSLGAVIGIVFHFRDHIMRLAVSGDTKVSKVTPEEEEQKDISVIGQAQEPAKVNI

>HzeaSNMP2

MCGIIGGVSTLVVGSVLVVASSVISFVFVPNIIRNIIAGEVTLLDDTIQMERFKEVPFPMNFTVRVFNMTNPAQVLTGGVPVMQEIGPYVYRLYQTREILEKDGDVVVYKMHEHFEFDADLSYPYQEDDLVTIINVPFHAVIQVAESLFPYLMSLLQMAMDEVFKEFNTPITTIRVRDLLFDGITMCKNPTGLGIIACSIIRDIADNAQNIEEKPDGSLVFSILNYKQQLPSQEYRVQRGLNDPADLGRILSYAGSPFFPQWLNLTTREPNVCMEVNGTDAGIFAPFVETERSIYAINTDICRSVELRYERDSEYEGIPTVRFAANEWLLDNDDGCFCLNVTRGINRDDGCLLRGAMELYTCVGAFLIMSYPHFLFADVRYRDSVLGMHPNEENHKIFIELEPNTGTPIRGAKRAQFNIFSRPVRNIPVTQNLRTAIVPILWIEEAIDLPHEFVDELTERLLSSLQLVDIFIPVLIAACVMVLVVGVALTARARLYRK

>HarmSNMP1

MQLPRELKYAAIAGGVALFGLIFGWVLFPTILKSQLKKEMALSKKTDVRKMWEKIPFALDFKVYIFNFTNAEEVQKGATPILKEIGPYHFDEWKEKVEVEDHEEDDTITYKKRDVFYFNPEMSAPGLTGEEIVVIPHIFMLGMALTVARDKPAMLNMVGKAMNGIFDDPPDIFLRVKALDILFRGMIINCARTEFAPKATCTALKKEGVSGLVLEPNNQFRFSIFGTRNNTIDPHVITVKRGITSVMDVGQVVAVDGKTEQTIWRDTCNEFQGTDGTVFPPFVPETERIESFSTDLCRTFKPWYQKKTSYRGIKTNRYIANIGDFANDPELNCYCAKPDTCPPKGLMDLAPCMKAPMYASMPHFLDSDPALLSKVKGLNPDVTQHGIEIDYEPITGTPMVAKQRIQFNIQLLKTDKLDLFKDLSGDIVPLFWIDEGLALNKTFVNMLKHQLFIPKRVVGVLRWWMVSFGSLGAVIGIVFHFRDHIMRLAVSGDTKVSKVTPEEEEQKDISVIGQAQEPAKVNI

>HarmSNMP2

MCGIIGGVSTLVVGSVLVVASSVISFVFVPNIIRNIIAGEVTLLDDTIQMERFKEVPFPMNFTVRVFNMTNPAQVLTGGVPVMQEIGPYVYRLYQTREILEKDGDVIVYKMHEHFEFDADLSYPNQEDDLVTIINVPFHAVIQVAESLFPYLMSLLQMAMDEVFKEFNTPITTIRVRDLLFDGITMCKHPTGLGIIACSIIRDIADNAQNIEEKPDGSLVFSILNYKQQLPSQEYRVQRGLNDPADLGRILSYAGSPFFPQWLNLTTREPNVCMEVNGTDAGIFAPFVETERSIYAINTDICRSVELRYERDSEYEGIPTVRFAANEWLLDNDDGCFCLNVTRGINRDDGCLLRGAMELYTCVGAFLIMSYPHFLFADVRYRDSVLGMHPNEENHKIFIELEPNTGTPIRGAKRAQFNIFSRPVRNIPVTQNLRTAIVPILWIEEAIDLPHEFVDELTERLLSSLQLVDIFIPVLIAACVMVLVVGVALTARARVYRK

>SexiSNMP1

MLLPKELKYAAIAGGVALFGLIFGWVLFPTILKSQLKKEMALSKKTDVRQMWEKIPFPLDFKVYIFNYTNAEEVAKGAVPILKEIGPYHFDEWKEKVEVEDHEEDDTITYKKRDVFYFNPEMSGPGLTGEETVVIPHVFMLGMALTVHREKPAMLNMVGKAMNGIFDDPPDIFLRVKAMDILFRGMMINCARTEFAPKATCTALKKEGVSGLVLEPNNQFRFSIFGTRNNSIDPHVITVKRGIKNVMDVGQVTAVDGQTVQTIWKDHCNEYQGTDGTIFPPFLTENDRLQSFSTDLCRSFKPWFQKKSSYKGIKTNRYVANIGNLAEDPELQCFCPQPDKCPPKGLMDLAPCIKAPMYASMPHFLDCDPALLSKVKGLNPDVNAHGIEIDFEPISGTPLVARQRLQFNIQLLKTDKLDLCKDLSGDIVPLFWIEEGLALNKSFVNMLKHQLFIPKRVVGVLRWWMVSFGSLGALIGVVFHFRDHIMRLAVSGDSKVSKVTPEEGEEQKDISVIGPAQEPAKINI

>SexiSNMP2

MLGKHSKLIFAVSMGFLVVAVIMAAWGFQKIVDKQIQSNVQLENNSMMFDKWLKLPMPLDFKVYVFNVTNVEDVNRGEKPILNEIGPYVYKQYRERTILGYGPNDTIKYMLRKRFEFDPVASGDLTEDDEVTVINFSYLAALLTVHDMMPSFVGMVNKALEQFFPSLEDAFLRVKVRDLFFDGIYLSCDGDNAALGLVCGKIKSDTPPTMRPAEGANGFYFSMFSHMNRSESGPYEMVRGRENVYELGNIVSYKGQKVMPMWGDKYCGQINGSDSSIFPPIKEGNVPKKLYTFEPDICRSVYVDLVGKKEIFNISAYYYEISESAFAAKSANPNNRCFCKKNWSANHDGCLLMGLLNLMPCQGAPAIASLPHFFLGSEELLEYFGSGIKPDKEKHNTYVYIDPTSGVVLSGLKRLQFNIELRQIDTVTQLKRVPTGLFPMLWLEEGATIPASIQQELRDSHKLLGYVEIARWFLLTVAIIAVVTSAVAVARANALLSWPRNSNSVSFILGPSVTHVNKGN

>SlitSNMP1

MLLPKELKYAAIAGGVAIFGLIFGWVLFPTILKSQLKKEMALSKKTDVRQMWEKIPFPLDFKVYIFNYTNAEEVAKGAVPILKEIGPYHFDEWKEKVDVEDHEEDDTITYKRRDVFYLNPELTAPGLTGEEIVVIPHVFMLGMALTVQREKPAMLNMVGKAMNGIFDDPPDIFLRVKAMDILFRGMIINCARTEFASKATCTALKKEAVSGLVLEPNNQFRFSIFGTRNNTIDPHVITVKRGIKNVMDVGQVVAVDGQTEQTIWKDTCNEYQGTDGTVFPPFLTENDRLQSFSTDLCRSFKPWYQKKSSYRGIKTNRYVANIGNLAEDPELQCFCPQPDKCPPKGLMDLAPCIKAPMYASMPHFLDCDPALLSKVKGLNPDVNAHGIEIDFEPISGTPLVARQRIQFNIQLLKTDKLDLCKDLSGDIVPLFWIEEGLALNKTFVNMLKHQLFIPKRVVGVLRWWMVSFGSLGAVIGIVFHFRDHIMRLAVSGDSKVSKVTPEEVEEQKDISVIGPAQEPAKINI

>SlitSNMP2

MCGVIGSVSTLVIGAILVIGSCIVGFLVVPNIVRNVIISEVVLNEDTIQMDRFEEIPFSLNFTVMIFNITNPETVLNGGVPFVTEVGPYIYRLYQTREILGIDGDIIRYKRHEHFVFDPVLSHPRTEEDILTIINVPYHAIIQVAETLYPNLMPLVNLAINGVFGKNNQPFVNITARELLFDGITLCKDTSLIATIVCNIIRNIAQGARNIEQLEDDSLVFSILDYKEQLPSEEYEVLRGLNDPADLGRILKYGGYNRFRHWAKNPEGGVTPCNQINGTDAGIYPPFVNREESIFAINTDICRSVELRYEYDTEYKGIPTYRYAANEWLLDNDEGCFCLNQTRGLNREDGCLLKGAMELYSCVGAFLVMSYPHFLFADNLYRNSVVGMWPDEDRHKIFVDIEPNTGTPIRGAKRAQFNIFSRPVNGVPVTQPFRTALVPILWVDESIVLPDEFVEELTGRLLHSLRLVDIFIPVIIAACGLVLVVGAGLTIRAFYVRKSVKKTESVPGDKTQPALETKPEPETQPEPLSQPEPRTENEVAK

>MsexSNMP1

MSHAVLFRTALAGKKLLNMRLARGIKYAVIGAGVALFGVLFGWVMFPAILKSQLKKEMALSKKTDVRKMWEKIPFALDFKIYLFNYTNPEEVQKGAAPIVKEVGPYYFEEWKEKVEIEDHEEDDTITYRKMDTFYFRPELSGPGLTGEETIIMPHVFMMSMAITVYRDKPSMMNMLGKAINGIFDNPSDVFMRVNAMDILFRGVIINCDRTEFAPKAACTAIKKEGAKSLIIEPNNQFRFSLFGLKNHTVDSRVVTVKRGIKNVMDVGQVVAMDGAPQLEIWKDHCNEYQGTDGTIFPPFLTQKDRLQSYSADLCRSFKPWFQKTTYYRGIKTNHYIANMGDFANDPELNCFCETPEKCPPKGLMDLTKCVKAPMYASMPHFLDADPQMLENVKGLNPDMNEHGIQIDFEPISGTPMMAKQRVQFNMELLRVEKIEIMKELPGYIVPLLWIEEGLALNKTFVKMLKNQLFIPKRIVSVIRWWLLSFGMLAALGGVIFHFKDDIMRIAIKGDSSVTKVNPEDGEQKDVSVIGQSHEPPKINM

>MsexSNMP2

MCGIIGSVVILCVGAILVVVSCLVTFIAIPSLIENFVINSVVLLEDTIQMERFEDVPFYLNFSVTIFNISNPNEVLNGGVAAVDESGPYVYRLYQGRNVSGLEDDLITYRKTEYFEFDAEASYPYTEDDIVTIINVPYHAIIQIAERNFPELMSVLPLAMNGIFGQYNNPIMNVRAGDLVIDKIPMCTRPGIIGTIACAAIREMASTADNIEVQDDGSVTFSILANKHNVASPKYEVYRGLTEPRNLGIIRHFDGLPYFNYWTDEEGEDGEVIKSRCNDINGTDSGIFPPLLNRNNNLYAINTDICRSVELRFQGEVEFEDIPAYRYSANEWFLDNDDGCFCLNTTAGITQENGCLFKGAMELYSCVGGFMVLSYPHFLYADLRYRNGILGMTPIEDDHKIFMDIEPLTGTPLKGAKRAQFNIFSRPVNGISATQNLRTTLMPIFWIEEAFELPSEFVDELTTRMIRPLHLLNILLPTLIAVCCMTLLVGIILTIRAKSKKPIVPTSAPGISQSK

>BmorSNMP1

MQLAKPLKYAAISGIVAFVGLMFGWVIFPAILKSQLKKEMALSKKTDVRKMWEKIPFALDFKIYLFNYTNAEDVQKGAVPIVKEVGPFYFEEWKEKVEVEENEGNDTINYKKIDVFLFKPELSGPGLTGEEVIVMPNIFMMAMALTVYREKPAMLNVAAKAINGIFDSPSDVFMRVKALDILFRGIIINCDRTEFAPKAACTTIKKEAPNGIVFEPNNQLRFSLFGVRNNSVDPHVVTVKRGVQNVMDVGRVVAIDGKTKMNVWRDSCNEYQGTDGTVFPPFLTHKDRLQSFSGDLCRSFKPWFQKKTSYNGIKTNRYVANIGDFANDPELQCYCDSPDKCPPKGLMDLYKCIKAPMFVSMPHYLEGDPELLKNVKGLNPNAKEHGIEIDFEPISGTPMVAKQRIQFNIQLLKSEKMDLLKDLPGTIVPLFWIEEGLSLNKTFVKMLKSQLFIPKRVVSVVCWCMISFGSLGVIAAVIFHFKGDIMHLAVAGDNSVSKIKPENDENKEVGVMGQNQEPAKVM

>BmorSNMP2

MCGIVVSIVILVVGASLVLVSTIVGFAVVPGIVENMIIDSVVLSDDSQQFERFQEVPFGLNFSVTLFNIENPAEVLEGGVPNLTERGPYIYRLRQNRIISNEEEDKLTYNRLENFEFNERASFPFTEDDIVTITNTPYHAILQVAESAFPEMASFLPMVMNGIFGQNNNAPIIDIRVGDLLFDGIPLCKDADLIGRVACNLIRNMTETSQNIETQDDGSMLFTVLAYKQNKPTAPYKVLRGLNDHTDLGRILSYNERSSLEYWVDEVDEEGESIEPSICNTIKGTDSAIFPPFVDTGNSIFALNADICRSVELRYQYDTEYEGIPTKRFSANEWFLDNEDGCYCLNVTKGITQENGCLLRGTMELFTCSGAFMVLSYPHFLYADPIYRNGVVGMNPVEDKHRIMLDIEPNTGTPVVGAKRAQFNIFIRPISGIVATDNIKTTLMPLFWVEETSRLPLNFVEEIQGRLLRPLNLLSILIPVIVAICFVILVIGILVTIRASSKRNKIKQ

>DpleSNMP1

MQLPKHLKIAGGCSAAAIFGVLFGWVIFPTILKSQLKKEMTLSKKTDVRKMWEKIPFALDFKVYLFNYTNPDEVHKGALPIVKEIGPYHFEEWKEKVEVEEHDDTDTIDYKRRDTFIFNKMLSGPGLTGEEVIVLPNVFMMGIISKVYIEKPAMLNMIGKAINSIYGNPTSIFSSVNVMDLLFRGTIINCNQNEFAPKAVCTALKKEGADKLIYEPNNQYRFSLFGKRNGTVDSQVVTVKRGMKNVMDVGKVVALDGKTEQDIWRDQCNELIGTDGTVFPPFLTEFDRLESFSTELCRTFKPWYQKKTSYNGIKTNRYIANIGDLANDPELQCYCDEPSSCPPKGLMNMAKCMGVQMFASLPHFYGCEQNIINNVKGLNPDVNEHEIVIDFEPITGTPMVAKQRVQLSMQLLKTDKIELCKNLPDVISPLFWIEEGIALNKTFVNMLKHQLFLPKRIVGVVRWLLVSSGLLGLLACLVFHFKDNIMRFAVSDPVAATKVNPEAEQKDNSILEPAKIEI

>DpleSNMP2

MCNFACGMVSLLGGLVLVAVSSFVAFGVVPGIIDGVIESEVVLINETEQFDRFEEVPFPLTFNVRLFNVSNADVVLNGGVPVVTEVGPYVYKLTQTREIEEMGEDSITYRRRDQFEFDPEASYPLTEDDRVTIANVAFHSILQMAERLFPGLMFLLNNILSGVFGEHSGPLMTVRVGDLLFEGVSICKDPGLIGLIVCSQIASIGANVRNLEVLDDGSLRFAVLKYKNDTVSEKYVVSRGLKDPRQMGIIASYNNSAFLTNWVNWMNDSGDVTPSTCNMVNGTDSGVFPPFVDRSSPVFALNTDICRSAELRYQYDSEYEGIPVARFSANEWFLDNEAGCFCLNTTTGITKEDGCLKKGAMELYSCVGAFLILSYPHFLYADFAYRNGVIGMKPFEDKHRIFVDLEPNTGTVIRGMKRAQFNVFMRPVTSITSTQNLRTTLMPIFWVEEGMELPEDYVDMLTTRMLNRLRLVDILIPVLITVCALVAVLGVVILIRVRYAKRNIAPSKSTTSITPTTYTIDN

>PmacSNMP1

MTLSPKTDVRQMWQKIPFPLDFKIYLFNYTNAEEVQKGAIPIVKEVGPYYFEEWKEKVEVEDHDDSDTITYKKLDTFYFKKELCGPGLTGEELIVLPHPFMMGTIGVVIRDKPAMLNMIGKAFNGIFDNPPDIFIRAKALDILFRGIVINCARTEFAPKALCTALKKEAANRLIFGPNNQYVFSLFGMRNGTTDSHVVTVMRGMNNVMDVGKVIAIDGKPKMEIFRDKCNEYEGTDGTVFPPFLTQSDRLESFSGDLCRSFKPWFQKESSYTGLKTNRYIANIGDYANDPDLQCYCDSPDTCPAKGMMDLVKCMGAPLYVSLPHFLDCDPKIGEAVKGLNPDVNEHEILIDFEPITGTPLVARQRVQFNIKLVKTEKIDLCKDLPDTIVPLFWIEEGLALNKTFTNMLKHQLFIPKRVVGVLRWLLVSFGLLTAMACILFHYKDNIMHFAVSNGSDSAAKIRPEETEQREISVIGEAREPPKVEM

>PmacSNMP2

MCGVICGVTTTVIGVVLVIVSTVLTFVFVPGVIEDLILNEVVLLDDTEQMERFQEIPFPLNFTIRFFNISNTEEVLAGGVPDLVEVGPYIYKSYQSREVESKEDDTISYRYMERLEFDAESSYPYTEDDVVTIVNPPYHLILQVAEKSFPDLMGIINSAMTGVFGRYSNPIADVKVRDLLLDGIRFCENPGLIASIVCSQIRTIGATSNNLVVQEDGSIIFALLISRIQPSSVYKVHRGINNPEDLGRIISLNETSRFYYWVDEEEGKPSVCNMINGTDSSIYNPFVDITKSLFAINTDICRSVELRYQYDIDYEGIPGYRFSANEWFLDNDDGCFCLNLTSGITREDGCLLRGAAELYSCVGGPMVLSYPHFLYADPIYAKGVKGMNPSVEDHRILLDIEPNTGTVLRGAKRAQFNIFLRPIISITATENFNSTLTPIVWLQESVLLPEEYVDQLKNRMLMPLNLVSILLPIVIALCSVVVVVGVVIFVRAKLLNKSPSMTTTT

>DpseSNMP1

MSTTKMHWSIVSAGVCVAVGGYCGWSNMVHKKVSVIADGSYKRVNNKVYINVTNDMIHGAIIVIGYVYKYRHKKVKHSRDGSKITYVNVHDDADASAYTDDRIVANMHMNAVRITDIGANRNSRNRTGVRIKRMRIRGKSWVHGIISIIDVKSVISNDGAVHNANKAVNDRSMVHTSVRYDGVRCINGIAKAICNIKSGSKTIRSDGSASGHKNGSGHDVYVHTGKGDMKVIKDDSHNVWNASTGTSVCNINGTDASSYRRGDSMYISADICRSVYADIYGIGYRYSIGNINDIGHDNCCVDKANVIKRKNGCYAGADTTCDAVITHMGASNYRKMIRGNDAKKHTVDVSTGTRGGKRVNMKSINRIGITNTTVMAIWVGINGMVAKKKINTKANIVHWAACGGAGVAISYYYKGRGAK

>DpseSNMP2

MLESTTPKMLHWSLIVSALGVCVAVLGGYCGWSLFPNMVHKKVEQSVILADGSEQYKRFVNLPQPLNFKVYIFNVTNPDMIQHGAIPIVEEIGPYVYKQYRHKKVKHFSRDGSKITYVQNVHFDFDADASAPYTQDDRIVALNMHMNSVLQISENDPGLALLLVHLNANLKAVFNDPRSMFVHTSVREYLFDGVRFCINPQGIAKAICNQIKESGSKTIREQSDGSLAFSFFGHKNGSGHDVYEVHTGKGDPMKVLEIQKLDDSHNLQVWLNASTEGETSVCNQINGTDASSYPPFRQRGDSMYIFSADICRSVQLFYQADIQYEGIPGYRYSIGENFINDIGPEHDNECFCVDKLANVIKRKNGCLYAGALDLTTCLDAPVILTLPHMLGASNEYRKMI

>AlepSNMP1

MALPKELKYAAIAGGVAVFGLIFGWVLFPVILKSQLKKEMALSKKTDVRKMWEQIPFPLEFKVYLFNYTNAEEVQKGAKPILKEVGPYHFDEWKEKVEIEDHEEDDTITYKRRDAFYFNQEMSAPGLTGEEIIVLPHVFMLGMAITVHRDKPAMLNVVGKALKGIFDDPADIFMRVKVLDLLFRGIIINCARTEFAPKATCTALKKEAVSGLVLEPNNQFRFSLFGTRNNSIDPHVITVQRGIQNVMDVGKVVAIDGKTEQTVWKDHCNEYQGTDGTVFPPFLTENDRIESFSTDLCRTFRPWYQKKTSYKGIKTNRYIANIGDFVNDPELQCFCPETDKCPPKGLMDLTPCMKAPMYASMPHFLDSDPALLNNVKGLNPDANQHGIEIDFEPISGTPMVAKQRIQFNIQLLKTDKLELCKDLSGDIVPLFWIDEGLALNKTFVKMLKHQLFIPKRAVGVLRWWMVSFGSLGTVIGIVYHFRDHIMRLAVSGDSKVSKVTPEEAEEKDISVIGPGQEPAKIDM

>AlepSNMP2

MCGIIGGAVTLGIGAALVLASSLIGYVWVPEIVKNVIVGEVALLDESVALERFETVPFSLNFTVRVFNVSNADAVRAGGVPVMHELGPYVYRLQQTRELVGQAGDVLRYRRHETFAFDAALSHPRTEDDLLTIINVPYHAITQVAESLYPALMPVLNLALSDVFGEYNAPFVTVPVRKLLFEGLRLCAPGAGLAGAVACDVIRGIAAGARNIAVQDDGSLLFSILDYKEQLPSEEYEVLRGTEDPADLGRILRYGESRYFSQWTNPPEGGMSVCNHINGTDAGIFPPFVDTSKSIYAINTDICRSVELRYEFDTEYQGVPTVRFAANEWLLDNNEGCFCLNQTRGLTRPDGCLLQGAMELYTCVGAFLVLTYPHFLFADVRYRDGVVGMRPIEENHKIFIDIEPNTGTPVRAAKRAQFNIFSRRVAGIPPTEQLRTALVPILWIEEAINLPEEFASELTDRLLRSLRLVDILIPVLIAFCGLILLAGVVLTVRARRRAATN

**e. CXEs used in fig 8**

>AlepCXE

MVQVRVSEGLLEGELVHNEYGGTYCSFKGIPYAQPPLGELRFKPPQPIKPWQGLRKAKEFGPVCYQFNTINPGLGNMSEDCLYLNVYTPNIKPDKPLPVMVWIHGGGFIWGSGNDDLYGPEYLIRHDVILVTLNYRLEVLGFLCLDTEDVPGNAGMKDQVAALRWIKKNIANFGGNPDNITIFGESAGAASVSYHLISPMSKGLFNRAIPQSGAATCFWAQAFEPREKALLLAKQLGFQSEDDKELYEFFKKQPVEALVFKNLQVTLGQKAYEIHFGVANEKDFGKERFFFGDQSDAIRNRTQEGVDIMTGYTRDEGLIAPSFAGPLEDIIAKASRYREMLAPKLIQIHCSIGDQLKAARKIQNFLYKDEPVSMKNHLKMFEFIGVDMFVYGVMLYAKLNSRKNKVYLYKFNCNSERNCFSNVFGVGHLVDNKTAVSHCDDLSYVFPVQLYTKKVDKNSDTFKLVDRVSKLWTNFAKYGDPTPDDSLGVKWKPYTLNEQNYLDIGNQLKAGKSPDKEENDLWEAVFQEYLPKYSALE

>AlepCXE20

MKNYLRSLSSWKLVVLISLWVAPLVPQPTAPVQLSGGMAQGSVAQDGAYLQYFGIPYATVKHRFQEAEPDPKWEGVFKANNEHIRCKQRFTSTRILGEEDCLTVNVYTPADTSDRPRPVMVFIHGGGFRDGSGSPFIYGPKYLVKHGVILVTFNYRLEVLGFLCLGIKEAPGNIGLKDQVLALKWVQQNIRAFGGDPDNVTIFGESAGGASVSYHLVSPMSKGLFKRAIMQSGSALGAWALQLEPLETASQLAKQMGHTTTDPIEIYNIFSNMTAEDLLKYRIPRKDGDIVISENVFVPCIEKKITDVSRFLPDSPYNLITQDKYNKVPVIIGFNSAEGLYFTGKENDTTLSKIDFYKALPRDLTFPTDEEKMKTANKLNELYMGGEKITKNKSSLDKLARYEGDAAITYSVLTTIDLLLQKQTEPVFAYKFNYDGLLNFAKMMSHTKSPGATHADELFYLFSTLTLPAIPEVRFIAKFTKLWTNFAKYGDPTPPSSPILPKWEPAALEDPRLLLIDKECSMEPIWDEADEAMRFWNKTYSLYRRKQ

>AlepCXE5

MSTGDKYAPGNNGMKDQVAALKWVQRNIAAFGGDPNLVTITGCSAGSISVMLHMISPMTKGLFHRGIAMSASPVNKDVLPLTHQRHLAVRQAQILNCPTDNSSVIVDCLMKKPWRELGDSLTKFWEFGDGDPVGLWGPVLEPDFGQERYLTLNPLDAIRQGTMHTVPLIVSQTTDEFFWKAFPVLQNATLLKTMNEEWESIAPISFMLPLQNRTLAVNRLKEVYLQGKPLANDKQSEKALGQLYGDSIIGFGVHRLANLMCRHSRHPVWYYEFAYIGNNSHYEDPNGKPQGAAHHDDLIYLFTLSYRYPVIPLSSPHSHVVDEMTALWYNFARYGDPNPRGDTPELGNLTWPAVLPRKRSYLHRGDQLVIRQNMFEDRFKVWEELYPIRY

>AlepCXE21

MLSRVWWLFVACVVCAASGQDADTQPSKTEDSPVTRSVSGQFRGSWMETRRGRRFQAYRGIRYAEPPVGELRFQPPKPILEYQSEVDASKEGPACPAPHSAGYYVDEDCLVINVYTPGTNSSKPLPVIFFMHPGGFYSFSGRSDVAGPHYLLDRDVVLVTINYRLGSLGFLSLGNKLAPGNNGFKDQVVALRWVQRNIAAFGGDPNLVTISGCSAGAVSVMVHMISPMSKGLFHRAIAVSGSPTSKLPSPTDRLDLAIKQARYSKCPEDNTTALYECLKTKTWKEIAGSLLGFFEFGYDPLSLWRPVVELDFGQERFLTEEPMVSIREGRMHSVPFIITQTTGEFFWKAFTVLNNQTLLDTMNAEWARIAPISFILPRNESSDKLARLRQAYLGDKPLVNDTANADGLGKLYGDSITGFPVHRMANLMCRHSAHPVYYGEFAYLGNHSHYEAGDPKRPTRTAHHDDLIYLFSLSYRFPTIDVADTPDSKMVDIMTALWYNFARYGDPKVQPDTPELSGVSWPAMTPDERNYLRLDKLFTVKQNLFEERFKVWDELYPIEY

>AlepCXE17

MLLKEAMSLFSVKWLVLWSLWAARLVRQPTDTLQLSNGPVRGSIAPDGSHRSYQNIPYASVPHRFQGPGPEPTWEKTFEAINENVRCAQSVTETWTIGRTECLSLNVYTPLNTSPDSKLPVMFFIHGGGFFQGSGNTFFYGPNYLVPKGVILVTINYRLNIQGFLCLGIKENPGNAAMKDQVAALRWVQRNIRQFGGDPDNVTIFGESAGAASVSFHLYSPMSKGIFHKAITQSGSALAAWALQFKPVYMASLLAKTMLYESQDPHELYKFFTTKSDDELILTRVPRAKGNTV

>AlepCXE13

MHLRLFLFLIVAGLVATQAPNPTIRVAHGLLQGTWKVSTKGRSFASFQGIPYARPPVGKYRFREPQPLKGWTGVWDATRFLPACLQYDPFIKKITGSEDCLYVNVFTPKMNPGANLPVVVFIHGGAFMYGEGAVYDPANLMDRDMVLVTLNYRLGPLGFLSTGDEMSPGNYGLKDQSFALHWVKNNILMFGGNPDSITLTGCSAGGASVHYHYLSPLSRGTFARGIAYSGSALTSWTHSIKPAEKAKALAAIVGCPTNTNKEMVDCLKYRPADVIVNAQIEMFEWRVHMFTPFTPVVEAPRVREPFLQEYPYHATRAGSMMNVPLITSVTSEEGLYPAAAYLEQPDILSDLDAHWNDLTANIFEYNDTLPLNLRSEVAMKIKQHYLGGKPVSQETFPQLVQALSDRLFVVDVGKTAQIHAAKSGQPTYVYRYAFRGATSLSNLMAHDDKNYGVSHADDVLRIFKYPGVDSNSPEDIAMTEGLIDMIYSFSTSGTPKLSNNGPAWTPVKPGAPELDYLDILSPTKMEMKSSTDFGQKSFWDSLGFNENENYRAYLRDEL

>AlepCXE31

MEWRTCVVLLCVTAVLADDDWLEVKIAQGPVRGRKHPNADLYEFYNIPYATAPTGVNKFKAPLPPPVWSEPFDAINKEVICPQPTFPYLDLMPKTIIQQENCLIANVYVPNTKEKNLSVLVYVHGGAFIMGYGDMIKATRYITRTKDFIMVNFNYRLGVHGFLCLGTEDVPGNVGMKDQVAALRWVQKNIAAFGGNPNDVTLAGGSAGSASVDLIMLSKLAEGLFHRVVPESGGNLAAFTVQRDPLEIAKKYAKQLNFTNVDDIYALEEFYKTAPLKLLTSDHFFDKTDSTFMFSPCVERDTGDGAFLTESPLSILKKGNYKKLPVLYGFANMEGSLRVMLYDYWKPKMNEKFSDFLPADLQFESVEEREEVADKIRKFYFGDEPIGDDNILAYVDFFSDVIFTYPMLWATKLQVEAGNNEIYLYEYSFVDDDDPVVPHTNVRGAPHCAQSMAVMDGKNMTHADDIYASPKYLKMKNVVRELWHNFLKTGKPVPKGSSLPTWPPAGADRSPHMVLAEKLELRGVLLEERTRFWDDIYQRYYRDAIPPPKP

>AlepCXE14

MEWRTCVVLLCVTAVLADDDWLEVKTAQGPVRGRKDPKADLYAFYNIPYATAPTGVNKFKAPLPPPVWLEPFDATNKEVICPQPTFPYLDLMPKTVVQLENCLIANVFVPNTKKKNLSVLVYVHGGAFIMGYGDMIKATQYITRTKDFIMVNFNYRLGVHGFLCLGTEDVPGNAGMKDQVAALRWVQKNIASFGGNPDDVTLAGGSAGSASVDLLMLSKSAEGLFHRVVPESGGNLAAFTVQRDPAEIAKRYARQLNFNNVEEIYALEDFYKTAPLELLTSDPFFDKTDSTFMFSPCVERDTGDGAFLTESPLSILKNGNYKKLPVLYGFANMEGLLRVMLYDYWKPKMNEKFSDFLPADLQFQSVEEREEVADKIRKFYFGDEPIGDANILAYVDFFSDVIFTYPMLWATKLQVEAGNNQIYLYEYSFVEDDDPVVPHTNVRGANHCAQSMAVMDGKNMTHADDIYASPKYLKMKNIVREIWHNFLKTGKPVPEGSSLPTWPPAGADRSPHMVLAEKLELRGVLLEKRTRFWDDIYQRYYRDAVPPPKPPPKGREDL

>AlepCXE4

MLWYGLLVLCSVTISTQNESDDWKLLQTAQGPVRGRKDPEGMYVFYNIPYATAPTGENKFKAPLPPPNWTEPFEAIDKGVICPQALVPLLHVETNTMQEDCLRANVYVPDTDEKNLSVMVYIHGGAFQIGFGKFITVKNLMKTKKLIVVNFNYRLGIHGFLCLGTKDVPGNAGMKDMVAMLRWVNKNIVSFGGNPNDVTIVGSSAGAAAADLLILSKSSKGLFHKVIIESASNLAEFTVQLDPLANAKMHAKTLNFTNVDDINSLEMFYKSASYDILTLDAFLERKDSTFVFVPCIERDSESTDEVFLVDSPINILKKGAYEKVPMLYGFANMEGLFRIQQFDTWSIEMNNKFSDFLPADLIFNSDQEKEEVAKMIKEFYFHDKPVGPDTVLGYVDYFTDILMAYPSLRSIKLNIAGGNHEIYLYEYSFVDEDTPFVPHTKIRGANHCAQTQAVADGVIFLNPDERNASRELMEMKKIIREMWHNFITTGKPVPEGSALPSWPALGANQSLYMSLGQKVELRDDLLKDRIDFWSSIYDKYYREPVPPPPSS

>AlepCXE18

MKCGKRIVLFTLFAMNLVDQPAPEVTIAQGTLSGKISTDGSFFEYVGIPYATTNSSTRFKAPLPPPSWEGVYKAVDEIYQCPQSSLFGIIGSEDCLKINVYVPALAKKPLPVMVYIHGGAFVLGSGGKFLYAPDFLVKHDVILVTFNYRLGALGFLCLGIKEAPGNAGIKDQIAALRWVKKNIAAFGGDPDNVTVFGQSAGATSASLLLASNATEGLFHKVIIQSGSSTSSWAINRQPLWVASLIAKELGYDVEDPKEIYEIFSKIPYEKLLKARPKKPLGMYFDTQLLNYPCVEREIEGEEAVITDFPYNIFNKNPKNIPVMYGTTNREGIFLIPDDTEESLAARNEKYIFASDLQFPSEELAANVSKMAREFYFGRKNISFEVQNTIINLNTQLYFEAPAILEAETLIKNTESNIFNYYFNYGGGRNFLKTISGFKNEPGACHSDEILYLFKGNIWPFPISRDDQKLIDWMTQMWTNFAKYGDPSPTNNLPVKWEPSKKGNMTFLYIDQELKMGPIPNPEANRLWKNIYEKYRT

>AlepCXE10

MVQVRVSEGLLEGELVHNEYGGTYCSFKGIPYAQPPLGELRFKPPQPIKPWQGLRKAKEFGPVCYQFNTINPGLGNMSEDCLYLNVYTPNIKPDKPLPVMVWIHGGGFIWGSGNDDLYGPEYLIRHDVILVTLNYRLEVLGFLCLDTEDVPGNAGMKDQVAALRWIKKNIANFGGNPDNITIFGESAGAASVSYHLISPMSKGLFNRAIPQSGAATCFWAQAFEPREKALLLAKQLGFQSEDDKELYEFFKKQPVEALVFKNLQVTLGQKAYEIHFGVANEKDFGKERFFFGDQSDAIRNRTQEGVDIMTGYTRDEGLIAPSFAGPLEDIIAKASRYREMLAPKLIQIHCSIGDQLKAARKIQNFLYKDEPVSMKNHLKMFEFIGVDMFVYGVMLYAKLNSRKNKVYLYKFNCNSERNCFSNVFGVGHLVDNKTAVSHCDDLSYVFPVQLYTKKVDKNSDTFKLVDRVSKLWTNFAKYGDPTPDDSLGVKWKPYTLNEQNYLDIGNQLKAGKSPDKEENDLWEAVFQEYLPKYSALE

>AlepCXE11

MMEEPVVAIEQGKIKGKVLKNFDDFEYCSFKGIPYAKPPIGELRFSVPQPPDSWQGIRDGTKDCNICAQFDKEEQAIKGDEDCLYLNVYTPKSFTTGDKSFPVMVFFHGGGFLFGNGTDDLSHGPDYLIHKKVVIVSINYRLGILGFLSLNIKEAPGNMGLRDQVQALKWVQKNIAHFGGDPKSVTIFGISAGAASVEYLLLSPMAKGLFHKAIAQSGSSLLHWAHEHHDNIRFLASRIPLQQGITVRDNYHLLQYLKSLPTRDLITGSMIALSAIQPRGGLYFGFVPTVEQPGDWEPFLNKLPYELLLKGEFHKVPYMTGFCTREGLLMISHKKPTLDKFIEDKVFVDYLPFCLDASEKNNAELRLKTNYLEAEKVYQDQDAYAIDFFSDVDFIGGIYVAATLIAKHNTPVFMYEFSYDGGLNYIKKCFNIDRPGACHGDDGGYIVKSDKLKGPISDMDTVIRNTMNQMWVNFARCGDPSPRLDFLVTTKWEPIPDTGIACLVIDRKMKMKYEIY

**f. CYPs used in fig 9**

>AlepCYP1

SSAVQCAVKYGRVCAARYEFYIRVLRGSSRCLFFVAMSQPLIANEVLAFIKHAIDYMDEV

SILQICKSNFNEEEISSAKLLLFQALGKVDQMQSRRRDGGQRSVQDIITMMKSTDPDDVP

AFVAKDLHKLPPVTFDHVDVTRLLKDITLLKQSQVEMQHQLEVSNNTISDLRAEVVLLRN

AISASRSPTDAANVNTRRGAQNASICSFESAGSNASPAAENACVATCAAAVPASTPVETT

TCVGSVTPKRAYAAIVAAAKKPVEPLNSQVKIADKEKGGKRSLPKPKQVLKRQAPVKDTGDSDGFIKVEKRKKKKPPCRNQCGTALAGPNTLLRPAVPTTLLYVSRLHHSTKVEEIVEYV

RVKTNWTLRVERLEPRHNTNFKSFVVRVPTHHLEKFLKEEFWP

>AlepCYP2

TTLGKLSQMPSRRRDGTERSIQDIVTLMKETDPDDVPEFVAKDLHKLPPVTFDHVDVTRL

LKDITLLKQSQVEMQHQLEVSNNTISDLRAEVVLLRNAISASRSPIDAAKVNTRRGAQNA

SICSFESAGSNASPAAENACVATCAAAVPASTPVETTTCVGSVTPKRAYAAIVAAAKKPV

EPLNSQVKIADKEKGGKRSLPKPKQVLKRQAPVKDTGDSDGFIKVEKRKKKKPPCRNQCGTALAGPNMLLRPAVPTTLLYVSRLHHSTKVEEIVEYVRVKTNWTLRVERLEPRHNTNFKSFVVRVPTHHLEKFLKEEFWPKGVVYAGFVAAYMTPVSETRX

>AlepCYP3

YANRQTFHTQMPLWVYLACVLVLCACAWLWRVERRHSSKFRGLPTYLVLPFIGVFQHVERLARICDNITSPLLAKLGPCYFLVTQDPEDIKTITNAFIEKPYYYNFGRIWLGDGLVTAPG

HIWKHNIKKLAGTFTSSVVDGFLGVFNAQAHKLVASLKTEVGKEPCDVIKKYLAYTTLEA

ICQTALGVSEISESIVTTKYYEAFNRCLELMTSRGLNILLHPDSIYRLTPAHREMTKCVA

>AlepCYP4

AMSRLACLLAVAGAASAQLTLDGIRCGQLVCQLEEYCSPDTNRCAPCNVVCNKTSHNYDAGLCIKECQGYLLDLRYLRRSEDTHPPGDLSGVQRQAQTALIISAVALAILVLVLIVICRG

KFSWRYLKQKFQPAKNRVKQYPSDITHHNPHAEMPKPKHELKLEIRNPEPAKRPQQPLNA

RDLETRTSQTEKSQGATTPKTISTALSNRHPAEDTTLDFSYDNMGMNVTPPEQPATSHKF

XGLVSFGGETVRFIEVPSNSTAASEISXQFVVKRTRFFSGFCDSSLQX

>AlepCYP5

XSNYQEEEISSAKQLLFQALGKADLMQSRRRGGGQRCVQDIITLMKSTDPDDVPVFVAKE

LHKLPPVTFDHVDVTRLLKDITVLKSNLAEVQSKLEVSNNTIGTLRTEVELLRNAFAESR

APSTSSKKTRRGKQNASIGSFESASSTMSPAANSACVALCPAAVVASSPGEVAPHVGAST

SERTYSAIVAAQKPAVLQTTRRGGKIKQDKLG

>AlepCYP324A6

MSFLQKKNPGDWLIDVYKKFNGAPYVGIWVFWRRGLVVNDSEIARRVLVKDADVFRNRNLASGRTDPMGALNLFTVDDPLWSSLRRRLTAVFTGSKLRGWQELYQSKVADLVYRIDSDNEKGITIDLRSIFADFSTDLVGESSFGIQCHATRDSTGPLREMTKEFEKYSLWRGLAWSSIFFLPEAVDVFRFSFWPKHTVQYFRRVFKQMVAERGGFERERDGKRDLLDALLKIKHDAIKNNEEMDLDILISNAMIFLQGGYETSSTGLVFIITELAHHPKEQQILYEELVKAKEALGGVEFDAENLSKIPYLEAVIKETLRKYPVMGWIDRVALKEYKIDENLTIPAKTVVYVNAIGMHKDPKYFPEPEKFKPERFLPENAADIVPYTYMPFGEGPRMCIGKRIGQNTLRYALAAVVLNFELLPLPQYPAPNDIPIEKRGMFYVPGIPLSVQFKRRK

>AlepCYP315a1

MYRSKRIPLLKLFKFNKWLSSDASISTQLSIEVMPRPKALPLLGTKLEFIAAGSGTKLHEYVDSRHKQLGPIYCEKLGGNADLVFVSEPTLIRTLLLNLEGKYPVHILPEPWELYEKLYGSKRGLFFMNGDEWLENRRVMNKYLLKENSENCFEAPVKKTVQNLIQKWKIEIGNGTYVPNLESEFYRLSIDVIIAVMLGTSSSLKPDIHYETLLAMFAETVKKIFQTTTKLYGLPVKMCQKWNLKVWRDFKESVDISLSLAHKIVNEMLLYSKDGNGLINALVEENVKPEDIKRIIGDFVIAAGDTTSYTSIWTLFLLSRNKNAIQEIRLRNSYINYVIRESMRLYPVAPFLTRILPKECNFGSYKLDKGTPIIASIYTSGRDERNYSKADEYLPYRWDRNDARKKDLVNHVSSASLPFALGARSCIGKKLAMLQMTQLISQIAHNFEIECLNKDEIKANTSQVLVPSQDIKLALSLQKPNIGQ

>AlepCYP4G75

MSYTAAESVVSTSTWAATSLFYVLLVPAVILWYAYWRMSRRHLYELAAKLHGPPGLPLLGNALEFTGGSHDIFRNVIEKSIPYDGESVVKIWIGPRLLVFLYDPRDVELILSSHTHIDKADEYRFFKPWLGDGLLISTGQKWRSHRKLIAPTFHLNVLKSFIDLFNANSRAVVNKLKKEAGEFDCHDYMSECTVEILLETAMGVSKSTQDQSGFEYAMAVMKMCDILHLRHTKIWLRPDLLFKLTDYAKKQTKLLDVIHGLTKKVIRRKKEEFNSGKRPTILQDYNTNTEETIKTTSVEGLSFGQSAGLKDDLDVDDADVGQKKRLAFLDLLLESSQSGVVISDEEIKEQVDTIMFEGHDTTAAGSSFFLSMMGIHQDIQDKVIDELDKIFGDSDRPATFQDTLEMKYLERCLMETLRMFPPVPIIARHLKQDITLPSCGKQVPAGTTVVVATYKLHRRPDVYPNPTKFDPDNFLPERSANRHYYAFVPFSAGPRSCVGRKYAMLKLKIILSTILRNFRVHSDLKESDFKLQADIILKRAEGFKVRLEPRKTTKAC

>AlepCYP4C1

MLFEVLILGVVALVAWLLFFKEEENPLDQLPGPPRLPIIGNSWELFQSPPDKLLDVLVNYTKQFGDRYVFKVMGLRVLHISGPADIEVVLAHSRNIKKSAPYDFLRDWLGNGLLLSTGADWHKRRKILTPTFHFNILKNFATVMEEKTQDMVQMLKKKNGEDVSLMPMVSDFTLFTICETAMGTKLDRDQTSATLDYKNAVLNIGLQMLRRITRIWMHNDLVYKNTQEGKEFGKTLSIARSFADNVIMDRKAQRAQNKGGEEVDTDGIGTKRRLAMLDLLLEAEEKGQIDIEGIRDEVNTFMFEGHDTTAIAITFGLMLLADYEDVQDQIYEEVQNVLCGAEHVTMSHLADLKYLEAVIKEILRLYPSVPFIGRDIVEDFKLGDILVKKGTSVDVHIYELHHREDLFPEAEKFKPERFLVNEMRHPYAYVPFSAGPRNCIGQRFAMQEMKTTISEICRNFKIVPKEKGYRPRLMADLVLRPVDPIYVKFVPRT

>AlepCYP321A7

MLFLPLCLLLTVITLGWYLLGRYNENYWKTRGVKFYSKNKVLGPFWDFFVSKQALFEIFGELYKEHRDQPVIGVGQLTTPSLFVIDPKNVQHVLSGDFQAFHHRGIESLEGEQLTDNITFMNGPRWKLMRKNMTPLFTANKLKNMYYIMDKCAQDFVTYLKDNPSTWKGDFFNSLMMYNNAAVCGAIFGIGSESIFDSPFLKLAKNLSQTNWKNQFKFLMFSLSPKLTSMLGIQLFKEYEDFFIGSINEVIKKRQQENVKKHDFADICVSLQKNGIMRDEQTGYEMEPTTGLLSAQAMFFFSAGVEPCANSIFSTLTLLCRHPEILEKVHKEIDEHFEKYNNKITYDVICEMEYVDKVLSEGLRRFPPIGMLTRQCCQDTVLPAGNVRVAKGTKIFTPIYDIHNDPAIYPNPEVFDPERFSKNKGPNDDVYMPFGMGNRTCIGARYAKLQVLAGMVHVLRNFTIKSNEANVDIVFAHDPLNVKLGNVDIQLIPRNIK

>AlepCYP6K2

MLVLLSCIVLLFSFLHFYRKWRKVKSFWAERGVPHYPPHPIVGSLTFLQRQNPAVWMKQVYNEFKSPYVGMWAFWRPGLVINCPELARRVLVKDHEVFKNRFLSSGKSDPIGGLNIFTVNDPTWSFLRRRLTVLFTAARLRSLNNLQTVKAKDLVQRIKDELNKEESTNLRDVCSDFTTDVIGEAAFGMTSEAVKTGDSVMRRVTRELVKFNLHRGLCWSSIFFFPELVDIFRFSLFPKDTLEELRHIFRTIIEQRGGFDKEIKESRDLLDALLKIRQEAAAENEEISEDLLLAQAAIFLLGGFDTSGVTLTWALYELAWNPLCQERLYQELLEAKQKNGGKNLDATTLSELTYINCVIKEALRKFPSMGWLDRIASQDYVIDENLTIPKGTVVYVNAVGMQQDPKHFPDPLVYDPDRFLPENERNITPYTFLPFGEGPRGCIGKRFGYQTVRCGLASIILNYEVRALPNMPRPNDVHIEKNGLFLGPDKKLSVEFILRNQ

>AlepCYP9

MIILLIWVAVLIAVLVLYLRQIYSKFHRYGVKHFRPVPLLGNMARVLFRYNHFIDDVLDLYRSFPEERFVGRFEFINELVVVRDIELVKKITVKDFEHFLDHRSIFSTSDSFFSRNLFSLKGQEWKDMRSTLSPAFTSSKIRVMVPFMVEVGDQMMSSLSKKIKESKSGYIDIECKDLTTRYANDVIASCAFGLKVDSHNDVDNEFYAMGKLSSTFKFRQMLMFFIISNAPKLAKVLKLDFLSNMSKQFFRKLVMETMENRELKNIIRPDMIHLLMEAKKGKLTHDDSKTNDEAAGFATVEESSIGQKEINRVWTDDDLIAQAVLFFIAGFETVSSGMSFLLYELALNPDVQDRLAKEIKENDVKNGGKFDFNSIQNMQYLDMVVSELLRMWPPGIALDRICTKDYNLGKPNDKAEKDFIVRKGTGLWIPTIGFHRDPQFFPNPDKFDPERFSEENKHNISSFAYMPFGVGPRNCIGSRFALCEMKVMAYQILLHMEVSPCERTTIPAKLDKETFNIRLEGGHWLRFRPRK

>AlepCYP4L4

MILLLVSVVLVLALLVSWISLVRESRRFNVQGPSPLPLVGNAHLFVVKSSEFLNLVHKLMLKYGLVFRVHFFSTPYVIICHSKYVEPLISSTEHITKGRSYSFLTCWLGQGLLTATGQRWKSHRKFLTPAFHFNILQNFLPVFCKNQRVLTEKLRAMADGRPVDMFPIIALAALDNVNESIMGVSMNAQNQSESEYVKSIEELSTIVTMRMQIPFFGEDAIFNLLPYKTKQDNALKVLHGQTNKVINARRQELKKANITTLNDSSDIGIKNKHAFLDLLLLAEIDGKKIDDEHVREEVDTFMFEGHDTTTSGIVYTLHCLSKRRDVQEKIYEELKTIFGSEMHRDPTYHELNQMKYLELVIKESMRLFPPVPLIERRIMRDCEVGELKLVKGTSVVINIFHIQRQPDLYEDPLEFRPERFEAPPKNPFSWLAFSAGPRNCIGQKFAMMELKITISEIVKNFFILPSSQEPELSADLVLRSKNGVNVKFMPRK

>AlepCYP304a1

MIAAIAAVLFIPYIFVYFYRNAYKRPDNFPPGPPSLPVYGAFWIVLAQLGFKEFIAAIHRFTDLSTAFKKLGEKYKTKVVGCFLGPVPSVIVNDSKLIKELLNREEFDGRMDIILFRLRSFWKKLGIFFTDGYFWHVQRRFSLRYLRDYGFGRRCDILESVLAGEIKEMIDLGASGPKYPAEMQIVKGDLVYMPYFFSVPFINGMMQVFSRSTFPRSEYHNLWDLARGTVLFQRSSTDLGGSLSLTPWLKDVLPNYSGFNSLVKGNQHLLDFFRKLIDETMETHDETYDRHFLDMYITKMKEEMKQKGRSTYSVDQLILTCTDYTFPASSAVQFVLAMLVERLLLQPEVQDKIHEELDRVVGRDRLPTLDDRRNCHYLEASIREIMRYDTTVPLAVPHRAMKTTEIGGYVVPEGTLITPNLTMLHTDKEIWGDPENFRPERFLTNGQLDLSLDKSLPFGAGRRLCAGETYARQSMFQVFAGFMQAFKVSTADGKPLLKPAQRIQGIITTIPEFWVKVTVRK

>Alep NADPH-cyPreductase

MSDSAQDVLKDAAAGAAAAAAAGGSLFSTFDIIVLVILLGGTIWWLYNSKKENKKDEILLSKYSIQAAGSIQVTENSFINKLKSSGRSLVVFYGSQTGTGEEFAGRLAKEGIRYKMKGMVADPEECDMEELMKLQEIPNSLAVFCMATYGEGDPTDNSMEFYEWLKNGEPDLTGLNYAVFGLGNKTYEHYNAVAIYLDKRLEELGATRVYELGLGDDDANIEDDFITWKDKFWPTVCEKFNIESTGEEELIRQFRLVTHGPDDIQPNNVFTGEIARLHSLQVQRPPYDAKNPFLAQITVNRELHKGGDRSCIHVELDISDSKMRYEAGDHVAVYPINDSSLVDRLGQLTGANLDEIFSLINTDQESSKKHPFPCPTSYRTALTHYVEITALPRTHILRELVEYCSDEEDKKKLMLMATNSQEGKALYQSFIAEACRNIVHILEDIPSCKPPLDHLCELLPRLQPRYYSISSSPKMYPETVHITAVVVQYKTPTGRLNKGVTTTWLADNKPEPGKPLPRVPVYIRKSQFRLPLQTQTPIIMVGPGTGLAPFRGFLQERAHARANGKEVGDSILYFGCRHRDQDYIYQEELEKYEQNGDVKLNLAFSRDQKEKVYVTHLIERDMDLLWDIIGNRNGHFYICGDAKNMAVDVRNIVLKTIQEKGKRTESEAVQFIKKLESMKKYSADVWS
